# Identification of inhibitors of the *Salmonella* FraB deglycase, a drug target

**DOI:** 10.1002/2211-5463.70001

**Published:** 2025-02-13

**Authors:** Jamison D. Law, Yuan Gao, Sravya Kovvali, Pankajavalli Thirugnanasambantham, Vicki H. Wysocki, Brian M. M. Ahmer, Venkat Gopalan

**Affiliations:** ^1^ Department of Chemistry and Biochemistry The Ohio State University Columbus OH USA; ^2^ Native Mass Spectrometry Guided Structural Biology Center The Ohio State University Columbus OH USA; ^3^ Department of Microbiology The Ohio State University Columbus OH USA; ^4^ Center for RNA Biology The Ohio State University Columbus OH USA; ^5^ Department of Microbial Infection and Immunity The Ohio State University Columbus OH USA

**Keywords:** drug discovery, high‐throughput screening, *Salmonella* FraB

## Abstract

Nontyphoidal *Salmonella* is one of the most prevalent causes of human foodborne illnesses worldwide, with no narrow‐spectrum antibiotics or vaccines available. Here, we seek to address this gap. During the host inflammatory response, *Salmonella* metabolizes fructose‐asparagine as a nutrient using proteins encoded in the *fra* operon. Deletion of *fraB* leads to a build‐up of 6‐phosphofructose‐aspartate, the substrate of FraB, and intoxicates *Salmonella*. Because *fra* genes are absent in mammals and most members of the human gut microbiome, FraB inhibitors are expected to have limited off‐target effects and offer prospects as potential therapeutics. To identify such inhibitors, we conducted a high‐throughput screening of small‐molecule libraries using a FraB activity‐based biochemical assay. We screened 131,165 compounds and identified 126 hits that could be obtained commercially for further characterization. When tested at 25 μm inhibitor in the presence of 1 mm 6‐phosphofructose‐aspartate, FraB activity was reduced ~ 30–100% by 65 compounds. Guided by preliminary cell‐based data, we further characterized six compounds (one triazolidine, two thiadiazolidines, and three triazolothiadiazoles) and found them to exhibit IC_50_ values from ~ 3 to 100 μm and *K*_I_ (inhibitor constant) values from ~ 1 to 29 μm. Native mass spectrometry revealed that all three triazolothiadiazoles were capable of binding FraB; we also obtained evidence that one of the triazolothiadiazoles binds FraB even in the presence of substrate. The recurrence of multiple pharmacophores bolsters prospects for farming more hits from compound libraries and for designing therapeutics against nontyphoidal *Salmonella*.

## Graphical abstract

A high‐throughput screen was used to identify inhibitors of *Salmonella* FraB, a drug target. Characterization of top hits (identified after an additional counter screen) revealed that some triazolidines, thiadiazolidines, and triazolothiadiazoles are mixed‐type inhibitors of FraB. Native mass spectrometry showed binding of three triazolothiadiazoles to FraB, and that one of the triazolothiadiazoles binds FraB even in the presence of substrate.

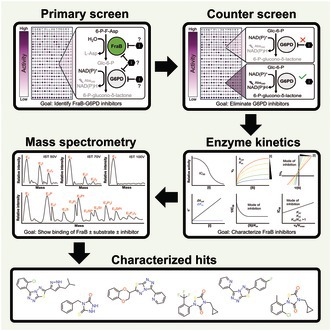

**Abbreviations**
6‐P‐F‐Asp: 6‐phosphofructose‐aspartate
F‐Asn: fructose‐asparagine
G6PD: glucose‐6‐phosphate dehydrogenase
HTS: high‐throughput screening

Diarrheal diseases pose a global economic and health burden as evident from estimates of mortality (1.5 million), prevalence (99 million), and incidence (6.6 billion) reported for 2019 [1]. Of 13 recognized etiological agents for diarrhea, *Salmonella* is ranked as the eighth leading cause of death following Rotavirus, *Shigella*, *Campylobacter*, Norovirus, *Cryptosporidium*, Cholera, and Adenovirus [1]. Among foodborne illnesses, nontyphoidal *Salmonella* is the leading cause of death and disability (adjusted for life years) [2]. Salmonellae are Gram‐negative, facultative anaerobes belonging to the Enterobacteriaceae family and consist of three species and 10 subspecies that collectively encompass over 2,600 serovars [3, 4]. Subspecies *enterica* primarily resides in mammals and accounts for 99% of salmonellosis in humans and other warm‐blooded animals [5].

*Salmonella* serovars can be classified as typhoidal or nontyphoidal [6, 7, 8]. Unlike typhoidal serovars, which are host‐restricted to humans and higher primates, nontyphoidal serovars are widely disseminated in animals, comestibles, and the environment, and can be either host‐adapted or have a broad host range [9, 10, 11]. The typhoidal serovars cause an invasive systemic febrile illness while the nontyphoidal serovars cause self‐limiting gastroenteritis marked by inflammatory diarrhea, fever, and abdominal cramps. Both the typhoidal and nontyphoidal serovars have been implicated in carcinogenesis of the gastrointestinal tract [6, 12, 13, 14, 15, 16]. Although some vaccines are now under development, none have been licensed thus far to protect against the nontyphoidal serovars [17, 18, 19]. Use of broad‐spectrum antibiotics is pursued only for patients who are very young or old, have underlying co‐morbidities, or develop invasive infections [20, 21, 22]. For uncomplicated cases, the use of broad‐spectrum antibiotics is disfavored because they delay convalescence, promote cooperative virulence, and prolong transmissibility [23, 24, 25, 26, 27, 28]. Instead, oral rehydration therapy is recommended but accessibility to clean drinking water is a crippling issue in developing countries as reflected in their higher‐than‐global annual average deaths from diarrheal diseases [1, 29].

Currently, there are no narrow‐spectrum drugs for treating *Salmonella*‐mediated enterocolitis. Widespread use of antibiotics in animal husbandry has also given rise to multiple drug‐resistant strains of *Salmonella* [30]. Companion animals (e.g., dogs and cats) may serve as reservoirs for drug‐resistant *Salmonella* with zoonotic potential [30, 31]. Both the CDC and WHO recognize drug‐resistant, nontyphoidal *Salmonella* as a serious threat for which novel effective therapeutics are needed [32, 33]. The dearth of *Salmonella* therapeutics could be attributed in part to the presence of redundant metabolic pathways and the versatile use of multiple nutrients by this pathogen [34, 35]. However, some catabolic routes include toxic intermediates that could be exploited as a vulnerability [36]. Here, we describe how we leveraged such an Achilles heel in *Salmonella* to address the dire need for narrow‐spectrum drugs. Specifically, we report the identification of small molecules that could serve as lead compounds for design of drugs that potentiate the killing of *Salmonella* by blocking the metabolism of fructose‐asparagine (F‐Asn), an Amadori compound [36, 37, 38, 39].

F‐Asn is present in some human foods and is formed by an Amadori rearrangement following the condensation of glucose and asparagine, a process that is facilitated by heating or drying [40]. Utilization of F‐Asn involves FraE (asparaginase), FraA (transporter), FraD (kinase), and FraB (deglycase), all encoded by the *fra* locus [37]. Once in the periplasm, F‐Asn is first deamidated to fructose‐aspartate (F‐Asp) by FraE (Fig. 1A). Following transport into the cytoplasm by FraA, F‐Asp is phosphorylated by FraD to form 6‐phosphofructose‐aspartate (6‐P‐F‐Asp), which is subsequently hydrolyzed by FraB to form glucose‐6‐phosphate (Glc‐6‐P) and l‐aspartate (l‐Asp) (Fig. 1A). Deletion of FraB results in accumulation of 6‐P‐F‐Asp and culminates in either a bacteriostatic phenotype *in vitro* or a bactericidal outcome in mice [38]. This phenotype is ameliorated by deletion of FraD, a clear indication that the toxic metabolite is 6‐P‐F‐Asp, a sugar phosphate [36, 38]. Since few species in the human microbiome have the *fra* locus and can utilize F‐Asn, FraB could be targeted for treating nontyphoidal *Salmonella* given the expectation of minimal perturbations to the host microbiota [41, 42]. Therefore, we conducted a high‐throughput screening (HTS) of small‐molecule libraries to identify inhibitors of *Salmonella* FraB.

**Fig. 1:**
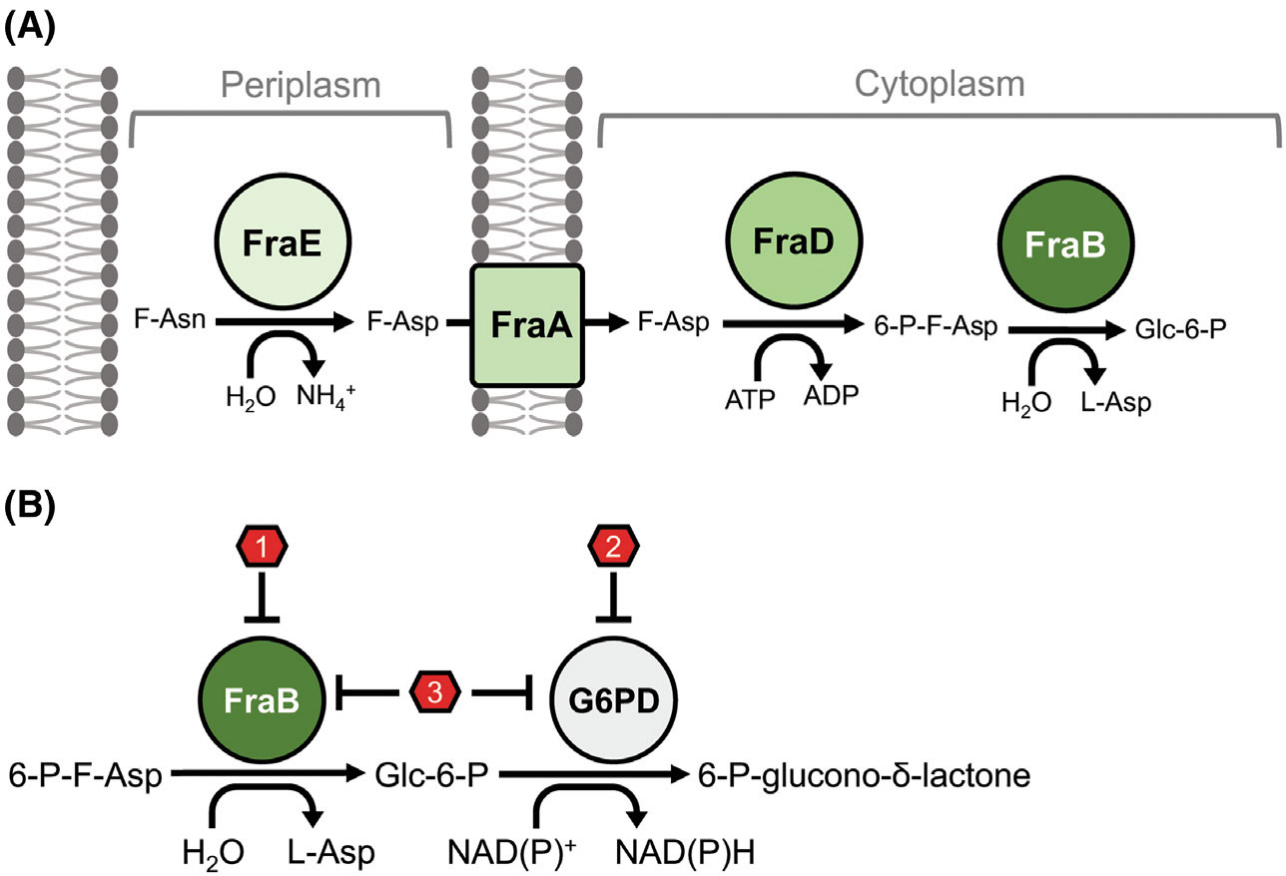
(A) Location and function of proteins encoded in the *fra* operon. In the periplasm, fructose‐asparagine (F‐Asn) is converted by FraE to fructose‐aspartate (F‐Asp), which is subsequently imported by FraA into the cytoplasm where it is phosphorylated by FraD, a kinase. The resultant 6‐P‐F‐Asp is then cleaved by FraB, a deglycase (the focus of this study), into l‐aspartate (l‐Asp) and glucose‐6‐phosphate (Glc‐6‐P). (B) Basis of the FraB‐G6PD coupled assay where activity is measured based on the conversion of NAD(P)^+^ to NAD(P)H [that absorbs at 340 nm, unlike NAD(P)^+^]; in this study, we used NAD^+^ and not NAD(P)^+^. Possible losses in activity may be due to a compound (indicated as a red hexagon) inhibiting only FraB (1), only G6PD (2), or both enzymes (3).

Our HTS sought to screen thousands of compounds as potential lead inhibitors of FraB and aid subsequent drug discovery. Because of the precedent in different HTS initiatives that many hits prove to be artifacts, we used filters in our biochemical assays to identify *bona fide* chemotypes that warrant further study. From a screen of 131,165 compounds, we identified at least three potential lead pharmacophores to inhibit FraB.

## Materials and methods

### Overexpression and purification of FraB

For protein overexpression based on the pET system, we used either *Escherichia coli* Rosetta(DE3) or SixPack cells, with the former grown in LB media supplemented with 35 μg·mL^−1^ kanamycin and 35 μg·mL^−1^ chloramphenicol and the latter grown in Dynamite media supplemented with 35 μg·mL^−1^ kanamycin [43, 44]. We elaborate our rationale for the switch in overexpression hosts and growth media. Lipinszki *et al*. [44] demonstrated that SixPack cells afford superior cell growth and amounts of heterologous protein compared with Rosetta2(DE3)pLysS. Taylor *et al*. [43] formulated Dynamite media to increase cell culture density by 10‐fold without incurring a proportionate increase in cost. Therefore, SixPack cells and Dynamite media were utilized in combination to maximize yields of FraB from a single culture and to obviate the need for multiple purifications. FraB purified from Rosetta(DE3) was used for the HTS and cherry picks, while FraB purified from SixPack cells was used for all experiments that followed after. For transformation, 50 μL of electrocompetent cells was mixed with either 10–100 pg (for SixPack) or 10–100 ng (for Rosetta) of pET‐33b–His_6_‐FraB, which encodes a His_6_‐FraB wild‐type (325 aa) variant, as described previously [39]. Electroporation was performed using a MicroPulser (Bio‐Rad, Hercules, CA, USA) programmed for a 5‐ms pulse at 2.5 kV. Transformed cells were diluted in 500 μL of LB media (no antibiotics) and incubated at 37 °C and 250 r.p.m. for 1 h. Following outgrowth, 200 μL of cells were spread on LB agar plates and incubated at 37 °C for 12–16 h. For the IC_50_ and *K*_I_ experiments, ‘heat‐shock’ SixPack cells were prepared as described by Yang *et al*. [45], except that the cells were grown in non‐autoclaved Dynamite media, and used for transformation as follows. About 100 ng of pET‐33b–His_6_‐FraB was mixed with 1× KCM buffer to a final volume of 25 μL, mixed with 25 μL of cells, and incubated on ice for 30 min. Cells were then diluted with 300 μL of SOC media, 100 μL of cells was directly plated on SOB agar containing 35 μg·mL^−1^ kanamycin, and the plate was incubated at 37 °C for 14 h.

To test for small‐scale overexpression, three well‐isolated colonies from each transformation were individually transferred to 1 mL of growth media (+100 mm glucose) and grown for 12–16 h at 37 °C with shaking at 250 r.p.m. These overnight cultures were used at a 1 : 100 dilution to seed fresh growth media and cultured using tube/flask : volume ratios that were no more than 1 : 4. The optical density (OD) was then measured at 600 nm using a WPA Biowave cell density meter (VWR, Radnor, PA, USA). When the small‐scale (5‐mL) cultures reached an OD_600_ ~ 0.6–0.8, they were induced with 1 mm IPTG for 2 h at 37 °C; in the case of SixPack cells, induction was with 10 μm IPTG at 20 °C for 18 h. Cells (3 mL) were harvested at 15,000 ***g*** for 2 min, resuspended with 500 μL of buffer A [50 mm Tris–HCl pH 7.5 (23 °C), 150 mm NaCl, 1 mm DTT], and sonicated with a Vibra‐Cell Ultrasonic Processor (GEX130) using a small bit (Vibra‐Cell 630‐0422) for a total of 60 s: 2‐s pulse with a 5‐s off‐interval and at a 50% amplitude. Lysed cells were clarified at 15,000 ***g*** and 4 °C for 5 min, and cellular extracts subject to SDS‐PAGE [15% (w/v) polyacrylamide] at 200 V for 45 min before visualizing with a mixture of glacial acetic acid, methanol, and water (1 : 3 : 6) with (for staining) or without (for destaining) 3 mm Coomassie Brilliant Blue R‐250. Small‐scale tests informed choice of colonies for large‐scale (500 mL) expression. Cells were harvested at 2,500 ***g*** and 4 °C for 15 min and the pellets (6 g per 250 mL) were stored at −80 °C until further use.

Purification of His_6_‐FraB was carried out as follows. A cell pellet (6 g) was resuspended with 25 mL of buffer A+ [buffer A + SIGMAFAST Protease Inhibitor Cocktail Tablet (S8830‐20TAB, 1003491512; Sigma‐Aldrich, St. Louis, MO, USA)] and chilled in an ice‐water bath (0 °C) for 10 min. Using a large‐bit probe (Vibra‐Cell 630‐0435 P), cells were lysed with a Vibra‐Cell Ultrasonic Processor (GEX130) for 2 min: 2‐s pulse with a 5‐s off‐interval and at a 50% amplitude. After a 5‐min rest, cells were mixed by inversion and re‐lysed as done before. Cells debris was pelleted by centrifuging at 30,000 ***g*** and 4 °C for 15 min in a SS‐34 rotor using the Sorvall RC 5B Plus centrifuge and the lysate supernatant was transferred to a new tube. The cell lysis pellet was resuspended (with 25 mL of buffer A+), re‐sonicated, and centrifuged as before. The pooled lysate supernatant (50 mL) was nutated with 2.5 mm MgCl_2_, 0.5 mm CaCl_2_, and 1 μg·mL^−1^ DNase I (Roche, Indianapolis, IN, USA) at 20 °C for 20 min and then clarified through a 0.22‐μm syringe filter (Millipore Sigma, St. Louis, MO, USA). Using a peristaltic pump at 1 mL·min^−1^, the filtered sample was applied to a 5‐mL HisTrap HP column (GE Healthcare, now Cytiva, Marlborough, MA, USA) and the flow‐through was collected. The column was then washed with 100 mL of buffer A and then with 100 mL of 10% buffer B (buffer A + 50 mm imidazole) prior to attachment to an AKTA FPLC (GE Healthcare). A linear gradient of 10–100% buffer B (100 mL total) was used to elute protein from the column in 1.5‐mL fractions, which were subsequently analyzed by SDS‐PAGE as described above. Fractions containing pure protein were pooled (50 mL total) and concentrated using a 3,000‐Da MWCO centrifugal filter (Millipore Sigma). Removal of the His_6_ tag was not possible due to the repeated inability of the Tobacco Etch Virus protease to cleave its recognition sequence in the context of the FraB version that is 325 instead of 331 amino acids (data not shown). Recombinant FraB (~ 200 mg·L^−1^) was established to be nearly homogeneous (Fig. S1). By comparison, around 20 mg·L^−1^ of recombinant FraB would be typically obtained from Rosetta(DE3) cultures grown in LB media. A NanoDrop 2000C Spectrophotometer (Thermo Scientific, Waltham, MA, USA) was used to measure the absorbance (280–320 nm) of purified His_6_‐FraB. Expasy ProtParam (https://web.expasy.org/protparam/) was used to determine the extinction coefficient, and molecular weight of His_6_‐FraB that, along with the corrected absorbance, was used to determine the concentration of the purified protein using the Beer–Lambert law. In all instances, the concentration refers to monomeric His_6_‐FraB.

### FraB assay

The substrate for FraB, 6‐P‐F‐Asp, was synthesized as previously described [38, 46]. FraB activity was monitored as described previously, with the following changes [39]. The final reaction (30 μL) consisted of 25 mm HEPES pH 8 (at 20 °C), 5 mm MgCl_2_, 0.1 mm ethylene glycol tetraacetic acid (EGTA), 0.5 mm NAD^+^, 1 mU G6PD from *Leuconostoc mesenteroides* (#LS003981; Worthington Biochemicals, Lakewood, NJ, USA), 100 nm His_6_‐FraB, and 0–3 mm 6‐P‐F‐Asp. The reaction mixture without substrate (29 μL) was added to one well of a 384‐well polystyrene white μClear® microplate (#781095; Greiner Bio‐One, Monroe, NC, USA) and incubated at 37 °C for 2 min, prior to the addition of 1 μL substrate to initiate the reaction. NADH generation was monitored using either absorbance (340 nm) or fluorescence (350 nm excitation, 450 nm emission) and with either an INFINITE M1000 PRO (Tecan, Mannedorf, Switzerland) or Synergy H1 (Biotek, Winooski, VT, USA) plate reader. Absorbance/fluorescence was plotted against time and slopes were obtained by linear regression analyses. These slopes were converted into initial velocities by dividing them by either the absorbance extinction coefficient (ε_340_ = 6,220 m ^−1^·cm^−1^) or the molar fluorescence determined based on standard curves obtained in different plate readers (23,646 RFUs·μm ^−1^, Tecan; 285,108 RFUs·μm ^−1^, Biotek; Fig. S2). These initial velocities (*v*_o_) in turn were plotted against [6‐P‐F‐Asp] to obtain Michaelis–Menten curves that were fit to the formula *v*_o_ = (*V*_max_ × [S])/(*K*_m_ + [S]) by Kaleidagraph (Version 4.5.3; Synergy Software, Reading, PA, USA). The activity of recombinant FraB (Fig. S3) was similar to that as reported previously [39].

### Screening small‐molecule libraries at the ICCB‐Longwood facility at Harvard University

Before engaging in the HTS campaign, the quality of the FraB‐G6PD assay was assessed using the *Z*′ factor, a dimensionless statistical characteristic that describes the signal dynamic range and data variation associated with an assay [47]. *Z*′ was calculated by *Z*′ = 1 − [3σ_c+_ + 3σ_c−_/|μ_c−_ − μ_c+_|], with σ and μ being the standard deviations and sample means of the positive (c+) and negative (c−) controls. Unlike the original description [47], we define the positive and negative controls as giving the minimum (complete inhibition) and maximum (no inhibition) signal, respectively. We first prepared two master mixes: (A) consisting of 1× FraB buffer [25 mm HEPES (pH 8 at 20 °C), 5 mm MgCl_2_, 0.1 mm EGTA] and 1 μm FraB; and (B) consisting of 1× FraB buffer, 1 mm NAD^+^, 400 μm 6‐P‐F‐Asp, and 26.6 mU·μL^−1^ G6PD, which were prepared. To serve as the positive control (no activity, a potent inhibitor), 15 μL of 1× FraB buffer was dispensed into the wells of column 24 of a 384‐well plate (3701; Corning, Corning, NY, USA) while 15 μL of master mix A was dispensed into all other wells. A robot (see below) then facilitated the addition of either 100% (v/v) dimethyl sulfoxide (DMSO) to wells in column 23 (negative control) and 24 (positive control) or 150 μm (typical concentration) compound to all other wells (see below). The absorbance of the plate was then read to detect compounds with inherent absorbances at 340 nm. Following incubation at 24 °C, 15 μL of master mix B was added to all wells. To ensure even mixing, plates were centrifuged 235 ***g*** for 10 s using a Heraeus Multifuge X3F Centrifuge and the TX‐750 rotor (Thermo Scientific). After a 30‐min incubation at 24 °C, Abs_340_ was measured to determine FraB activity.

Buffers and master mixes were dispensed using a small tube plastic tip‐dispensing cassette (#24073290; Thermo Scientific) attached to a Multidrop Combi (Thermo Scientific), which was programmed to HIGH speed, 0.0/0 mm dispense offset, 16.00 mm dispense height, 2 mL predispense, and Greek key style of direction. Prior to each use, the dispensing cassettes (different for each of the two master mixes) were washed with 20 mL (10 tubing volumes) of water, 20 mL of 70% (v/v) ethanol, and 20 mL water and then followed by a priming rinse with 1× FraB buffer. An Epson E2C2515‐UL Scara robot with a 300‐nL pin array (V&P Scientific, San Diego, CA, USA) was used to facilitate transfer of compounds, while an Epson C3‐A601S 6‐axis robot was used to deliver 384‐well plates to and from deck. After every library transfer, the pin array was successively washed twice each with phosphate‐buffered saline (PBS), 100% (v/v) methanol, and 20% (v/v) methanol (with sonication) before a final 10‐s high‐pressure air‐drying step. Each transfer, including the washing and drying steps, took ~ 1 min. To ensure even mixing, plates were centrifuged using the Heraeus Multifuge X3F Centrifuge (Thermo Scientific, Waltham, MA, USA) as described above. Endpoint absorbance measurements were read with an EnVision 2103 Multilabel Reader (PerkinElmer, Waltham, MA, USA).

Hit identification relied on a decrease in Abs_340_ in the presence of a compound compared with the negative control (full FraB‐G6PD activity). Therefore, compounds with high inherent Abs_340_ values exceeding that of the negative control were discounted from further consideration. Inhibitors were classified as strong, moderate, or weak if their absorbance value was 0–25%, 25–50%, or 50–75% of the mean of the negative controls, respectively. To narrow down an initial list of ‘cherry‐picked’ compounds, we undertook the following steps. First, we determined for each compound a *Z*‐score [*Z* = (*x* − μ)/σ] where *x*, μ, and σ correspond to the test value, mean of the negative controls, and standard deviation of the negative controls, respectively. Second, we calculated the *Z*‐score for the positive controls to set the threshold for the lowest possible *Z*‐score. Third, we divided the test *Z*‐score by the positive control *Z*‐score and multiplied by 100. Compounds with %*Z*‐scores of 0–20, 20–40, 40–60, 60–80, and 80–100 were classified as null, weak, moderate, strong, and very strong inhibitors, respectively.

### Ascertaining the effect of DMSO on FraB‐G6PD activity

In‐house testing of compounds is facilitated by pipettes where accuracy becomes a concern when handling small volumes (300 nL). Pipetting larger volumes of compounds (where accuracy is higher) is preferable, but increasing (DMSO) may adversely affect enzyme activity. Therefore, we assessed the effect of DMSO on FraB activity as follows. The final reaction (30 μL) consisted of 25 mm HEPES pH 8.0 at 20 °C, 5 mm MgCl_2_, 0.1 mm EGTA, 0.5 mm NAD^+^, 1 mU G6PD from *L. mesenteroides* (Worthington Biochemical, code: ZFL, catalog # LS003981), 100 nm His_6_‐FraB, 1 mm 6‐P‐F‐Asp, and 0–50% (v/v) DMSO. The reaction mixture, lacking DMSO and substrate, (14 μL) was added to 24 wells of a 384‐well polystyrene white μClear® microplate (Item No.: 781095; Greiner Bio‐One) and 15 μL of either water or 5, 10, 20, 40, 60, 80, or 100% (v/v) DMSO was then added to three of the wells. The plate was then incubated at 20 °C for 20 min, prior to the addition of substrate (1 μL), whereafter the fluorescence (350 nm excitation, 450 nm emission; read from bottom of plate) of NADH was monitored for 10 min at 37 °C using an INFINITE M1000 PRO (Tecan) plate reader. The fluorescence (RFUs) of NADH was plotted against time for each sample, and the average and standard deviation were determined from three repeats for each concentration of DMSO tested. Each average and standard deviation were divided by the average of the negative control (water) and multiplied by 100 to obtain the relative average activity and standard deviation, which was then plotted against DMSO % (v/v) concentration. FraB retained > 90% activity with 10% (v/v) DMSO, the highest concentration used in any of our assays (Fig. S4).

### Screening of cherry‐picked compounds

For the G6PD assay, master mix A (500 μm Glc‐6‐P in 1.25× FraB buffer) and master mix B (1× FraB buffer, 1 mm NAD^+^, and 66 mU·μL^−1^ G6PD) were prepared. To a 384‐well plate, 12 μL of either 1.25× FraB buffer (positive control) or master mix A and 3 μL of either 100% (v/v) DMSO (negative control) or 1.5 mm of the cherry‐picked compound was added. The plate was incubated at 20 °C for 30 min prior to and following the addition of 15 μL of master mix B. Endpoint fluorescence measurements were taken using the Synergy H1 (Biotek) plate reader. For the FraB‐G6PD assay, master mix A (1.25 μm FraB in 1.25× FraB buffer) and master mix B (1× FraB buffer, 1 mm NAD^+^, 400 μm 6‐P‐F‐Asp, and 66 mU·μL^−1^ G6PD) were prepared. The remainder of the assay was carried out as described above.

### Screening of purchased compounds

Commercially available compounds were ordered from the corresponding vendors and resuspended in 100% (v/v) DMSO to yield 10, 20, 25, 40, 50, or 100 mm stocks (depending on solubility). Ambinter compounds were stored as aliquots under desiccated conditions and protected from light (with aluminum foil covering) at −80 °C. For G6PD assays, 26 μL of either 1.15× FraB buffer (positive control) or master mix (0.038 mU·μL^−1^ G6PD in 1.15× FraB buffer) and 3 μL of either 100% (v/v) DMSO (controls) or 2.5 mm compound were added to a well of a 384‐well plate. After a 20‐min incubation at 20 °C, 1 μL of either water (positive control) or 1.5 mm Glc‐6‐P was added to initiate the reaction. The final reactions consisted of 1× FraB buffer, 1 mU G6PD, 50 μm Glc‐6‐P (the amount typically generated over 20 min in the FraB‐G6PD assay), and 250 μm compound. G6PD activity was continuously monitored using fluorescence (350 nm excitation and 450 nm emission; read from bottom) using the INFINITE M1000 PRO (Tecan) plate reader. For FraB‐G6PD assays, 26 μL of either 1.15× FraB buffer (positive control) or master mix (0.038 mU·μL^−1^ G6PD, 115 nm FraB, and 1.15× FraB buffer) and 3 μL of either 100% (v/v) DMSO (controls) or 2.5 mm compound were added to a well of a 384‐well plate. After a 20‐min incubation at 20 °C, 1 μL of either water (positive control) or 6 mm 6‐P‐F‐Asp was added to begin the reaction. The final reactions consisted of 1× FraB buffer, 100 nm FraB, 1 mU G6PD, 200 μm 6‐P‐F‐Asp, and 250 μm compound. FraB‐G6PD activity was continuously monitored using fluorescence (as described above). FraB‐G6PD assays were repeated as described above using 25 μm compound with either 0.2 or 1 mm 6‐P‐F‐Asp. The average slopes of the activity curves for each assay were used to determine percent inhibition relative to the negative control.

Compounds obtained from vendors were given activity scores according to the formula *Z* = 1 − ([μ_t_/μ_c_] × 100) where μ_t_ and μ_c_ refer to the average slopes (RFU_450_ versus time) of test compounds (t) and negative controls (c). Relative standard error (SE) was calculated using SE = ((σ/√*n*) × 100)/μ_c_, where σ is the standard deviation and *n* is the sample size. A compound was classified as a *bona fide* hit if it had an activity score of < 20 for the G6PD assay and > 30 for the FraB‐G6PD assay.

### Characterization of top hits

For determining the IC_50_ value of each compound, 26 μL of master mix was added to two rows (48 wells) of a 384‐well Greiner plate followed by 3 μL of inhibitor to four wells per concentration (12 concentrations total) using a single‐channel pipette. After mixing with a 12‐channel pipette, the plate was incubated at 20 °C for 20 min. To initiate the reaction, a 12‐channel pipette was used to add and mix 1 μL of 7.5 mm 6‐P‐F‐Asp to all 48 wells. The final reaction (30 μL) consisted of 25 mm HEPES pH 8.0 at 20 °C, 5 mm MgCl_2_, 0.1 mm EGTA, 0.5 mm NAD^+^, 1 mU G6PD, 100 nm His_6_‐FraB, 0.25 mm 6‐P‐F‐Asp, and varying concentrations of inhibitor (10% v/v DMSO). NADH fluorescence was monitored every 10 s for 10 min total on a SPARK® multi‐mode multiplate reader (Tecan), as described above, and plotted against time. Slopes of the linear range of each curve were converted to initial velocities, as described above, that were then plotted against (inhibitor). Kaleidagraph was used to fit a sigmoidal curve to each plot using the following formula in order to determine the [IC_50_]: % inhibition = 100/1 + (IC_50_/[I]), where I is [inhibitor] [48].

To determine the *K*_I_ of each inhibitor, 29 μL of master mix was added to two rows of a 384‐well Greiner plate, using a single‐channel pipette, and incubated at 20 °C for 20 min. A 12‐channel pipette was then used to transfer and mix 1 μL of varying [6‐P‐F‐Asp] to all 48 wells. The final reaction (30 μL) consisted of 25 mm HEPES pH 8.0 at 20 °C, 5 mm MgCl_2_, 0.1 mm EGTA, 0.5 mm NAD^+^, 1 mU G6PD, 100 nm His_6_‐FraB, varying (6‐P‐F‐Asp) (between 8 and 12 concentrations), and varying [inhibitor] (between three and five concentrations) in 10% (v/v) DMSO. NADH fluorescence was monitored every 10 s for 10 min total on a SPARK® multi‐mode multiplate reader (Tecan), as described above, and plotted against time. Slopes of the linear range of each curve were converted to initial velocities, as described above, that were then plotted against [6‐P‐F‐Asp]. A hyperbolic curve was then fitted to the data to obtain the *k*_cat_ and *K*_m_ values according to the formula: *v*_o_ = (*V*_max_ × [S])/(*K*_m_ + [S]).

To determine the correlation between the observed and expected initial velocities (Fig. S5A), these values were exported from Kaleidagraph, plotted against one another, and a curve was fit using the formula: *y* = *mx* + *b*. The differences between observed and expected initial velocities (residuals) were then depicted as box‐and‐whisker plots (Fig. S5B). Lineweaver‐Burk plots were generated by graphing 1/*v*_o_ versus 1/[S] and curves were fit to the data with Kaleidagraph using the formula: 1*/v*_o_ = *(K_m_/V*_max_).(1/[S]) + (1/*V*_max_). The *k*_cat_ and *K*_m_ parameters were then converted into α′ values by dividing the *k*_cat_ of the control (no inhibitor) by either itself (α′= 1) or the *k*_cat_ of test samples with variable [inhibitor]. Those α′ values were plotted against [inhibitor] and *K*_I_ values were determined from the equation α = 1 + [I]/*K*_I_.

The initial velocities used to generate the Michaelis–Menten plots were also employed to obtain the IC_50_ vs [S]/*K*_m_ plots [49]. For a given [S], initial velocities were converted into relative activities and plotted versus [inhibitor]. Each IC_50_ was determined as above and plotted against its corresponding [S]/*K*_m_ value, calculated by dividing the test [substrate] by the *K*_m_ (~ 40 μm). The IC_50_ values determined from the IC_50_ vs [S]/*K*_m_ plots were then used to plot 1/IC_50_ versus *v*_o_/*V*_max_. A curve was fit to the data using the formula: *y* = *mx* + *b*. The *Y*‐intercept of the 1/IC_50_ versus *v*_o_/*V*_max_ plot is equal to 1/*K*_IC_, and the slope, for mixed inhibitors, is equal to 1/*K*_IU_ – 1/*K*_IC_. *K*_IU_/*K*_IC_ ratios of 1, < 1, and > 1 correspond to noncompetitive, mixed‐type uncompetitive, and mixed‐type competitive modes of inhibition, respectively [50]. Data analyses were performed as described above (see Tables S1–S7 for curve‐fit errors and goodness‐of‐fit values).

### Native MS studies

For MS analysis, FraB was buffer exchanged into 200 mm ammonium acetate (AmAc) (Sigma‐Aldrich) or 66.7 mm ethylenediaminediacetic acid (EDDA) (Sigma‐Aldrich) using micro Bio‐spin P6 spin columns (Bio‐Rad); AmAc and EDDA concentrations were chosen to ensure the same ionic strength. The pH was adjusted to 7.5 by adding ammonium hydroxide solution (Sigma‐Aldrich). 6‐P‐F‐Asp was diluted with water, while three different inhibitors [3470:A09, 3469:F02, and 3469:N17, all purchased from either ChemDiv (San Diego, CA, USA) or Ambinter (Orléans, France)] were diluted in DMSO. The final concentration of DMSO used in the native MS experiments was 4.8% (v/v) or 1% (v/v) for the compounds obtained from ChemDiv or Ambinter, respectively.

For the characterization of FraB–inhibitor complexes, the protein was diluted into 200 mm AmAc and then mixed with inhibitors to final concentrations of 3 μm FraB and 120 μm inhibitor. After adding the inhibitors, the FraB–inhibitor complex was incubated for 20 min at 20 °C before injecting into the mass spectrometry (MS). For the characterization of FraB–inhibitor–substrate complexes, FraB was diluted into 66.7 mm EDDA, mixed with inhibitors, incubated at 20 °C for 20 min, and subsequently mixed with 6‐P‐F‐Asp. The final concentrations were as follows: 3 μm FraB (monomer concentration), 120 μm inhibitor, and 600 μm 6‐P‐F‐Asp.

Native MS experiments were performed on Thermo Q Exactive ultra high mass range (UHMR) Orbitrap [51, 52]. Samples were introduced into the MS instrument by nano‐electrospray ionization, which using glass capillary tips pulled in‐house using a P‐97 micropipette puller (Sutter Instruments, Novato, CA, USA). Each sample (3–5 μL) was loaded into the tips, and the electrospray voltage was set between 0.6 and 1.0 kV. The mass spectrometer was operated in positive mode using the following tuning settings: Capillary temperature: 200 °C detector optimization: low *m/z*; ion transfer target: high *m/z*; in‐source trapping (IST): 50 V, except when applying higher IST to disrupt the FraB–inhibitor interactions; HCD trap gas flow: 5; Resolution: 12 500. Mass spectra were collected after the spray stabilized (typically < 5 min) and then examined using  XCalibur 4.1 (Thermo Scientific), and deconvolved using UniDec software [53].

## Results

### HTS assay design and validation

To identify inhibitors of FraB that could be exploited as narrow‐spectrum antibiotics against *Salmonella*, we performed a HTS of small molecules at the ICCB‐Longwood Screening Facility, Harvard University. Since FraB does not generate a product that either absorbs or fluoresces light, we employed a previously described FraB‐G6PD coupled assay [39] that affords an NADH (Abs_340_)‐based measurement as a proxy for FraB activity (Fig. 1B, Fig. S6). Such a coupled assay has the caveat that hits will need to be filtered for their ability to selectively inhibit FraB and not G6PD (Fig. 1B, inhibitor class 1).

To determine assay robustness and reproducibility, we screened in duplicate the plates 3769–3772 from the Asinex1 library. For each plate, we compared the Abs_340_ values from the first (*X*‐axis) and second (*Y*‐axis) screen, both before and after substrate addition, and performed a linear regression analysis to assess agreement between replicates (Fig. S7). Such an analysis revealed positive correlations for plates 3769, 3770, 3771, and 3772 with slopes of 1.01, 0.93, 1.03, and 0.64, respectively (presubstrate addition), and slopes of 1.0, 0.95, 1.03, and 0.67, respectively (postsubstrate addition), and *R* ^2^ ≥ 0.86 (Fig. S7).

We then calculated our assay's *Z*′, a dimensionless parameter that can vary between 0 and 1 and describes the signal dynamic range and data variation [47]. We obtained *Z*′ values ranging from 0.77 to 0.87 for all eight independent sets of positive and negative controls. Encouraged by the high *Z*′ values of our assays (*Z*′ > 0.5 reflects an excellent assay) and considering the practical limitation of the large amount of in‐house synthesized substrate needed for technical replicates, we performed the remainder of the HTS in singlicate. Moreover, our rationale was that the overarching goal of the primary screen was to identify as many positives as possible that could be subsequently triaged.

We describe two aspects of the HTS assay, as they are instructive while examining the final outcomes. First, roughly 20% of the 131,165 compounds tested had Abs_340_ values that exceeded the average negative control value prior to addition of substrate (Fig. S8). Although both absorbance‐ and fluorescence‐based methods for performing HTS are known to be susceptible to compound interference due to quenching, inner filter effects, and autofluorescence [54], we had not recognized the full scope of this limitation. Future HTS projects would benefit from a compilation of the UV–Vis spectra of all compound libraries before a specific assay is chosen. For our in‐house post‐HTS studies, however, we shifted to a fluorescence‐based readout. Second, for screening the compound libraries at the ICCB‐Longwood Screening Facility as well as the cherry picks (a subset of compounds provided *gratis* for further testing), we used 1000 mU of G6PD to ensure a robust coupled assay. We later realized that even 1 mU (1000‐fold lower amount) of G6PD was sufficient for rapid turnover of the small amount of Glc‐6‐P generated by FraB. Nonetheless, the use of 1000 mU G6PD in our HTS may have been a fortuitous choice. A lower amount of G6PD in the initial HTS would have led to ‘hits’ that would have ultimately turned out to be false positives. By holding the untargeted ‘coupled (G6PD)’ enzyme in excess over the targeted enzyme (FraB), the sensitivity of our assay was increased and the likelihood of detecting G6PD inhibitors (false positives) was minimized.

### Identification of hits

**Fig. 2:**
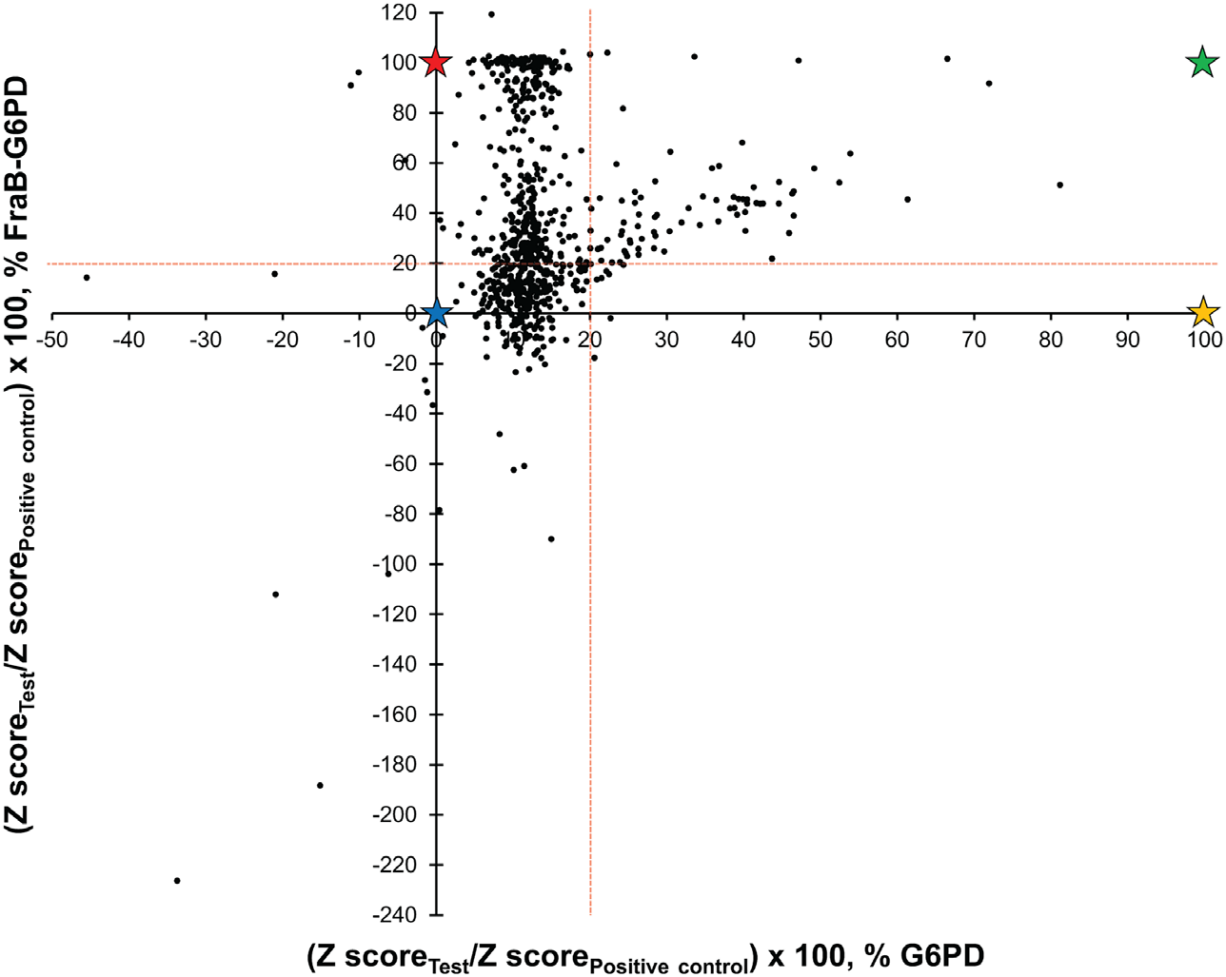
Volcano plot showing the % *Z*‐score of positive control for the FraB‐G6PD and G6PD assays with the cherry‐picked compounds. The red‐colored dotted lines indicate the cutoffs for a compound to be considered an inhibitor in either assay. The star symbols indicate theoretical/expected data for compounds that completely inhibit FraB (red), G6PD (orange), both FraB and G6PD (green), and neither FraB nor G6PD (blue). Negative values arise due to the inherent fluorescence of compounds that exceed the fluorescence of the negative control. The positive controls for the G6PD and FraB‐G6PD assays lack substrate (Glc‐6‐P) and enzyme (FraB), respectively.

**Fig. 3:**
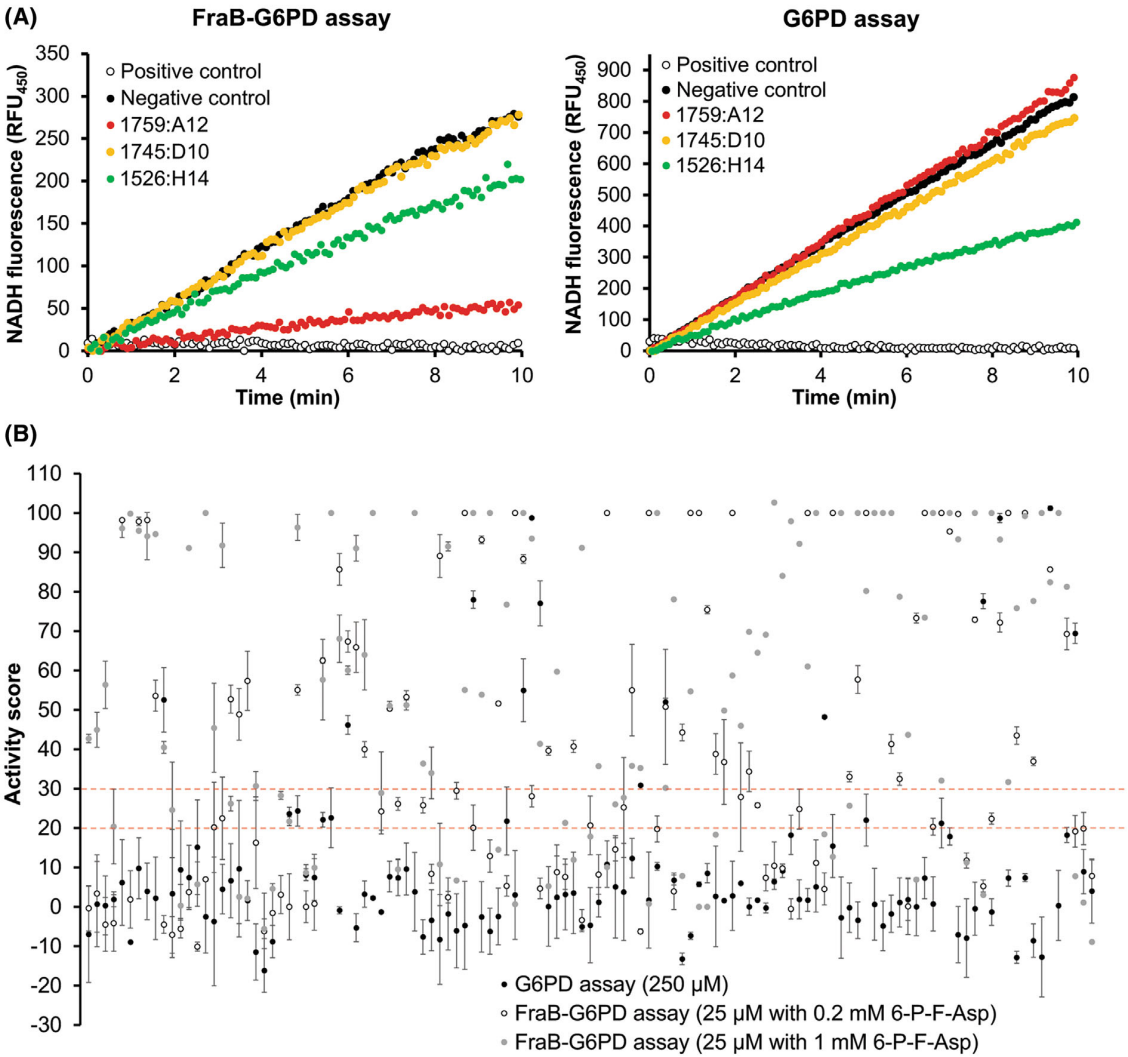
(A) NADH fluorescence (350 nm excitation, 450 nm emission) generated by G6PD and FraB‐G6PD activity over time in the presence of either 1759:A12 (FraB inhibitor), 1745:D10 (inhibitor for neither enzyme), and 1526:H14 (G6PD inhibitor). The positive control lacks the appropriate substrate (S) and inhibitors (I), whereas the negative controls contain the appropriate substrate without inhibitors. Although the coupled assay indicates inhibition by both 1759:A12 and 1526:H14, the latter needs to be discounted as a hit given its ability to inhibit G6PD. (B) Activity scores for the G6PD (black circles) and FraB‐G6PD (white circles) assay in the presence of 250 μm (for G6PD) or 25 μm (for FraB‐G6PD, with 0.2 or 1 mm 6‐P‐F‐Asp) compounds obtained commercially. Error bars indicate standard deviation (*n* = 3).

Even after discounting one‐fifth of the 131,165 compounds that exhibited high Abs_340_ values, we identified 97 strong, 171 moderate, and 644 weak inhibitors of FraB‐G6PD (see Materials and methods for the scoring criteria). These top 912 hits (~ 0.7% of total compounds tested) were cherry‐picked and obtained from the screening facility for further testing at our home institution (OSU). Since the HTS utilized a G6PD‐based coupled assay, it is possible that a subset of the initial winners targeted G6PD (Fig. 1B, inhibitor 2 or 3). Therefore, cherry‐picked compounds (at 150 μm) were first tested against G6PD in a counter screen. This filtering process was guided by the *Z*‐scores, which measure the relationship of a raw score to the mean of a group of values. We compared the *Z*‐score of a test compound to that of the *Z*‐score of the positive controls (i.e., 100% inhibition; assays in which FraB was intentionally omitted); the same analysis was performed for the G6PD assay. A volcano plot was then used to segregate winners, that is, FraB‐specific inhibitors with minimal or no effect on G6PD activity (Fig. 2, see Fig. 3A for representative examples). After eliminating G6PD inhibitors, 174 inhibitors of FraB were identified; of these, 126 were commercially available and purchased, five of which we found to be insoluble.

### Winnowing down the hits

**Fig. 4:**
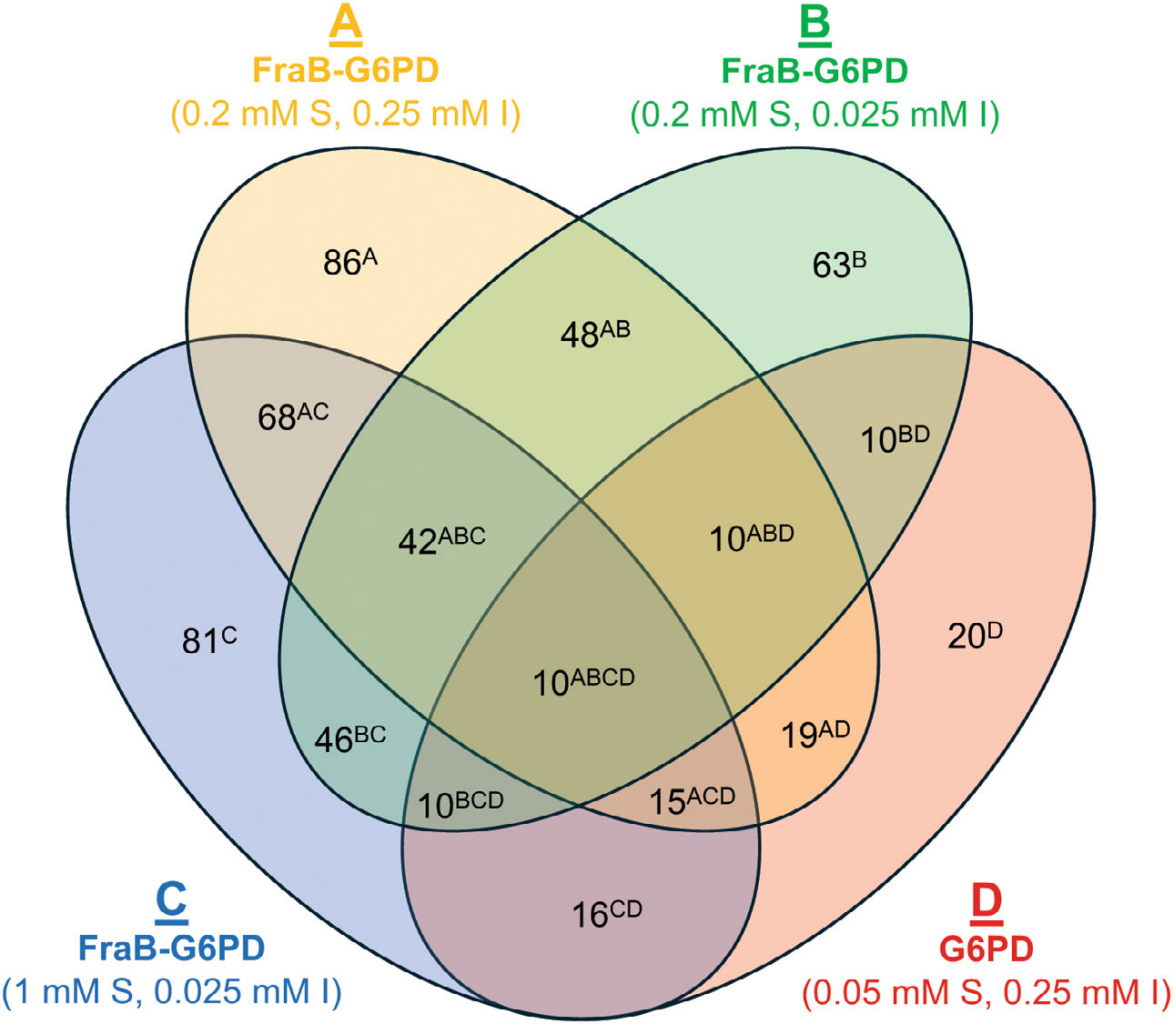
Hit overlap summary of compounds that either inhibit G6PD by more than 20%, using 0.05 mm Glc‐6‐P and 0.25 mm inhibitor (D, red oval), or FraB‐G6PD by more than 30% using either 0.2 mm 6‐P‐F‐Asp and 0.25 mm inhibitor (A, orange oval), 0.2 mm 6‐P‐F‐Asp and 0.025 mm inhibitor (B, green oval), or 1 mm 6‐P‐F‐Asp and 0.025 mm inhibitor (C, blue oval). The number of hits that are specific to FraB for one or more assays can be determined by subtracting hits that are also common with G6PD. For example, hits common in assays ‘A’, ‘B’, and ‘D’ (e.g., 10^ABD^) can be subtracted from hits common in assays ‘A’ and ‘B’ (e.g., 48^AB^) to arrive at the number of FraB‐specific hits common in assays ‘A’ and ‘B’ (e.g., 38).

We first tested the 121 commercially obtained compounds (Table S8), each at 250 μm against G6PD in the presence of 50 μm Glc‐6‐P. Despite eliminating G6PD inhibitors from the initial 912 cherry picks, 20 compounds were found to inhibit G6PD by > 20% (Figs 3B and 4, 20^D^) likely due to differences in the amount of G6PD used here (1 mU) versus the amount used for testing the cherry picks (1000 mU). Compounds were then tested against FraB‐G6PD at 250 μm with 0.2 mm 6‐P‐F‐Asp [five‐fold over the *K*_m_ (~ 40 μm)]. From this experiment, 67 compounds were found to inhibit G6PD by < 20% and FraB‐G6PD by > 30% (Fig. 4, 86^A^ – 19^AD^). When re‐tested at 25 μm and 0.2 mm 6‐P‐F‐Asp, 53 compounds were identified as inhibiting G6PD by < 20% and FraB‐G6PD by > 30% (Fig. 4, 63^B^ – 10^BD^). Because three compounds (not among the 126), identified from a previous cell‐based HTS, were found to inhibit the growth of *Salmonella* and were shown to be uncompetitive inhibitors of FraB [55], we re‐screened the 121 compounds at 25 μm with 1 mm 6‐P‐F‐Asp (25‐fold over the *K*_m_). Here, our objective was to identify potential uncompetitive inhibitors that may have been missed while testing at 0.2 mm 6‐P‐F‐Asp. Indeed, 65 compounds were identified that inhibited FraB‐G6PD by more than 30% and G6PD by < 20% (Fig. 4, 81^C^ – 16^CD^).

There is an incomplete overlap in hits when comparing FraB‐G6PD assays under different concentrations of substrate or inhibitor. Of the FraB‐specific hits, only 38 (i.e., 48^AB^ – 10^ABD^), 36 (i.e., 46^BC^ – 10^BCD^), 53 (i.e., 68^AC^ – 15^ACD^), and 32 (i.e., 42^ABC^ – 10^ABCD^) compounds were common between FraB‐G6PD assays A (0.2 mm 6‐P‐F‐Asp and 0.25 mm compound) and B (0.2 mm 6‐P‐F‐Asp and 0.025 mm compound), B and C (1 mm 6‐P‐F‐Asp and 0.025 mm compound), A and C, and among A, B, and C, respectively (Fig. 4). This analysis supports the value of iterative testing, as such efforts led to identification of hits that we missed while testing them at the lower substrate concentration.

The abovementioned lack of overlap may also arise from other technical issues, such as compound aggregation, solubility issues, changes in effective concentration due to adsorption on plastic, or differences in modes of inhibition. Also, batch‐to‐batch differences in substrate purity may impact FraB activity and compound efficacy. The *k*_cat_, *K*_m_, and *k*_cat_/*K*_m_ values of 63 ± 10 min^−1^, 0.116 ± 0.012 mm, and 543 min^−1^ mm ^−1^ were previously reported for FraB [39]. However, newer batches of substrate (synthesized and purified by Jason West, Mitton‐Fry Laboratory, OSU) gave *k*_cat_, *K*_m_, and *k*_cat_/*K*_m_ values of 57 ± 0.7 min^−1^, 0.042 ± 0.007 mm, and 1,357 min^−1^ mm ^−1^. Finally, because we initially tested hits at 0.25 mm, a high concentration that could have promoted aggregation, we could have missed some G6PD inhibitors. Despite all these caveats, we finally had 90 FraB‐specific hits among the three FraB‐G6PD assays (after discounting 20 compounds that inhibit G6PD and 11 compounds that inhibit neither FraB nor G6PD).

There are likely several contributing factors that led to the identification of only 90 hits from screening ~ 131,000 compounds (Fig. 4, Fig. S9). One possibility is that FraB may be more intractable than some other targets because it does not offer an accessible binding pocket. Another contributing factor is that the chemical space in the libraries tested may have been a sparse sampling of the chemotypes that are capable of specific interactions with binding sites in FraB. Our triage process led to loss of viable candidates at three different stages, and it is instructive to consider each. In the first stage, roughly 20% (~ 26,000) of the compounds were untestable due to their high absorbances, an issue that could have been ameliorated by using fluorescence‐ or luminescence‐based assays. In the second stage, only 174 compounds out of the 912 cherry picks from the primary screen were initially found to be specific for FraB. In the third stage, only 90 out of 121 of the purchased compounds proved to reproducibly inhibit FraB. Differences in G6PD amounts used for screening the cherry picks (1000 mU) vs the purchased compounds (1 mU) may explain the persistence of some G6PD inhibitors (*n* = 20).

Multiple freeze–thaw cycles of the compound libraries at the screening facility may have led to solvent evaporation and precipitation, which in turn could affect the concentration and potential degradation/transformation obligatory for generation of the ‘active’ component [56, 57]. Compounds were stored under desiccated conditions at −20 °C at the screening facility whereas they were stored under non‐desiccated conditions at −20 °C at OSU. Given that DMSO is highly hygroscopic, these different storage conditions may have affected the extent of hydration and thus the rate of degradation and precipitation [58]. Metal contaminants that were introduced during compound synthesis and not chelated by EGTA may also have generated false positives [59], if different compound batches had variations in this regard.

### Characterization of top hits

Interestingly, none of the 121 compounds registered as actives against *Salmonella* wild‐type in a cell‐based growth assay (this study; Sabag‐Daigle, A. & Ahmer, B. M. M., unpublished results). We sought to examine the possibility that some of the hits in the biochemical HTS were not picked up in the cell‐based assays due to efficient efflux mechanisms in *Salmonella*. Because TolC is an outer membrane protein channel that is essential for the ability of seven of the 10 inner membrane drug‐efflux transporters to expel structurally diverse molecules [60], we generated a *Salmonella tolC* mutant and tested the 121 compounds that we purchased (Sabag‐Daigle, A. & Ahmer, B. M. M., unpublished results). The tests were conducted in parallel using the *tolC* mutant in a wild‐type or *fra* island mutant background; the latter lacks the entire *fra* locus and is incapable of catabolizing F‐Asn and forming 6‐P‐F‐Asp. Thus, any inhibitor specific for FraB (and which was being effluxed in the wild‐type) will inhibit the *tolC* mutant in a wild‐type but not the *fra* island mutant background. A caveat is that efflux of compounds may also be achieved via TolC‐independent drug‐efflux pumps.

**Fig. 5:**
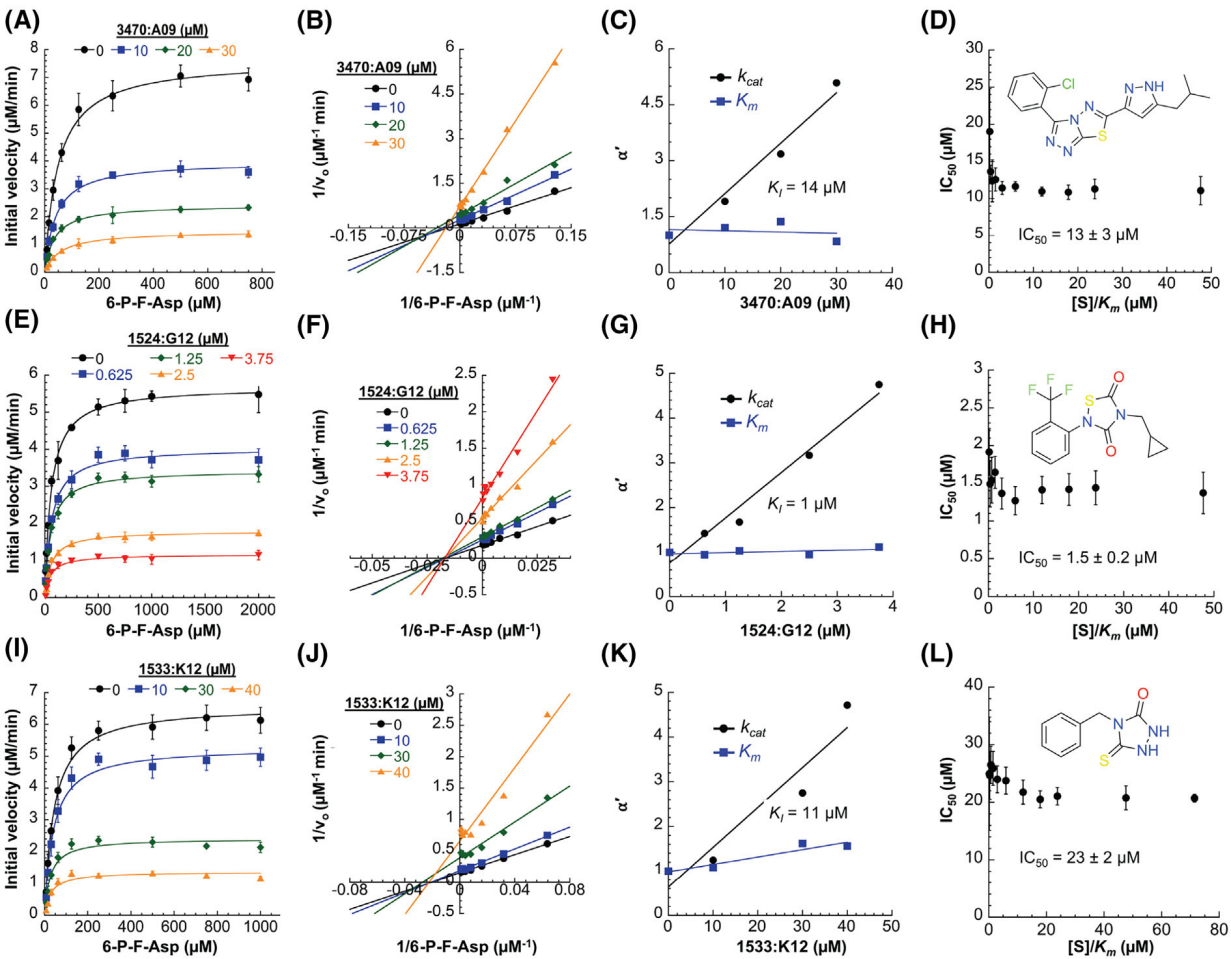
Characterization of the inhibition of *Salmonella* FraB by 3470:A09 (A–D), 1524:G12 (F–H), and 1533:K12 (I–L). Michaelis–Menten (A, E, I), Lineweaver‐Burk (B, F, J), α′ vs [I] (C, G, K), and IC_50_ vs [S]/*K*_m_ (D, H, L) plots from a single trial are shown here. Data from a second independent trial for each of these inhibitors are shown in Fig. S11. Each Michaelis–Menten plot depicts the average and standard deviation from triplicate or quadruplicate measurements at the indicated substrate concentration. In panels C, G, and K, α′= 1 + [I]/*K*_I_′. Averages and standard errors for the *K*_I_ values determined from two separate trials are listed in Table 1. In the IC_50_ vs [S]/*K*_m_ plots, the mean and standard deviation values determined by taking all the IC_50_ values are listed. Curve‐fit data for all the plots are listed in Tables S1–S6.

Twelve compounds, including three triazolothiadiazoles (i.e., 3470:A09, 3469:F02, 3469:N17), were initially shown to inhibit the growth of *Salmonella tolC* in a *fra*‐dependent manner; after multiple attempts with stocks from different suppliers, we were unable to replicate this early finding (data not shown). Nonetheless, owing to the recurrence of the triazolothiadiazole chemotype in the biochemical assays (at least nine molecules), we characterized the inhibition of FraB by 3470:A09, 3469:N17, and 3469:F02. Given the first round of in‐house screening, some of the pilot studies were performed with compounds obtained from ChemDiv; for the final characterization reported here, the compounds were purchased from Ambinter (as they were no longer available from ChemDiv). We found that these three triazolothiadiazoles were inhibitors with IC_50_ values ranging from 15.4 to 100 μm, in the presence of 0.25 mm 6‐P‐F‐Asp, and *K*_I_ values ranging from 10.5 to 28.5 μm (Fig. 5, Table 1, Figs S5 and S10–S12, see Tables S1–S6 for primary data and curve‐fit parameters). In addition to the triazolothiadiazoles, we sought to characterize the inhibition potential of compounds from two other prominent chemotypes of the 121 hits tested—cyclopropyl‐substituted 1,2,4‐thiadiazolidine‐3,5‐dione (1524:E12, 1524:G12) and 1,2,4‐triazolidine‐3‐one (1533:K12) variants. The cyclopropyl‐substituted 1,2,4‐thiadiazolidine‐3,5‐dione variants had IC_50_ values ranging from 2.8 to 4.3 μm and *K*_I_ values ranging from 1 to 4 μm; the 1,2,4‐triazolidine‐3‐one variant had an IC_50_ of 27.6 μm and a *K*_I_ of 12 μm (Fig. 5, Figs S5 and S10–S12).

**Table 1:** Summary data for hits characterized in this study.

**IUPAC name**	**PubChem compound ID**	**Source ID** ^a^	**Vendor**	**Vendor reagent ID**	**IC_50_ (μm)** ^b^	***K*_I_ (μm)** ^c^, ^d^
2‐(2‐chloro‐6‐methylphenyl)‐4‐(cyclopropylmethyl)‐1,2,4‐thiadiazolidine‐3,5‐dione	2810370	1524:E12	Ambinter	Amb2740107	4.3 ± 0	4 ± 0
4‐(cyclopropylmethyl)‐2‐[2‐(trifluoromethyl)phenyl]‐1,2,4‐thiadiazolidine‐3,5‐dione	2810371	1524:G12	Ambinter	Amb19665875	2.8 ± 0.4	1 ± 0
4‐benzyl‐5‐sulfanylidene‐1,2,4‐triazolidin‐3‐one	2366666	1533:K12	Ambinter	Amb20635649	27.6 ± 3.9	12 ± 1
3‐(2‐chlorophenyl)‐6‐[5‐(2‐methylpropyl)‐1H‐pyrazol‐3‐yl]‐[1,2,4]triazolo[3,4‐b][1,3,4]thiadiazole	16421618	3470:A09	Ambinter	Amb821229	15.4 ± 0.4	10.5 ± 3.5
6‐(4‐fluorophenyl)‐3‐pyridin‐2‐yl‐[1,2,4]triazolo[3,4‐b][1,3,4]thiadiazole	16764760	3469:N17	Ambinter	Amb1098473	100 ± 13	28.5 ± 3.5
6‐(2,3‐dihydro‐1,4‐benzodioxin‐3‐yl)‐3‐pyridin‐2‐yl‐[1,2,4]triazolo[3,4‐b][1,3,4]thiadiazole	17598362	3469:F02	Ambinter	Amb830196	42 ± 2.6	28.5 ± 3.5

^a^ ICCB‐Longwood designation including the source plate ID and the well ID.

^b^ Concentration that inhibits activity by 50% in the presence of 0.25 mm 6‐P‐F‐Asp; the mean standard error determined from two measurements is listed. Reported values reflect IC_50_ data in Fig. S5.

^c^ The inhibitor constants were determined from Michaelis–Menten analysis of FraB at different inhibitor concentrations (see Fig. 5, Fig. S6); the average and standard error determined from two measurements are listed.

^d^ See text for additional details on the mode of inhibition.

Examining changes in the apparent *k*_cat_ and *K*_m_ (α′) as a function of [inhibitor] is instructive in revealing the type of reversible inhibition. Consistent with the Lineweaver‐Burk plots, the six compounds exhibit mixed inhibition characteristics (Fig. 5, Figs S11 and S12). Mixed‐type inhibitors can bind to both E and ES. However, depending on whether the mixed‐type inhibitor binds with higher affinity to either E or ES, these inhibitors may either increase or decrease the *K*_m_ in addition to eliciting an adverse effect on *k*_cat_; they can also bind to E and ES with the same affinity. With the six hits tested, changes to the *K*_m_ are minimal and in fact not the same extent in both trials but the *k*_cat_ changes (i.e., *k*_cat_ decreases by α′, where α′= 1 + [I]/*K*_I_) are significant and reproducible. While all of them could be classified as mixed‐type, 1524:E12 and 1524:G12 are pure noncompetitive inhibitors (no change in *K*_m_). To further corroborate the mode of inhibition, we examined IC_50_ versus [S]/*K*_m_ [49] and 1/IC_50_ versus *v*_o_/*V*_max_ [50].

For a noncompetitive (mixed‐type) inhibitor, the IC_50_ values are not expected to vary as a function of [S]/*K*_m_. Indeed, 1524:E12 and 1524:G12 exhibit this trend (Fig. 5, Fig. S11). The IC_50_ values should decrease with increasing [S]/*K*_m_ for an uncompetitive inhibitor and vice‐versa for a competitive inhibitor. We observed a modest decrease with 3470:A09, 3469:N17, 1533:K12 and a slight increase with 3469:F02 (Fig. 5, Fig. S11). With these four inhibitors, the change in IC_50_ values is evident only at low [S], that is, from 0.2 to 1.6× the [S]/*K*_m_ and not between 1.6 and 75× (Fig. 5, Fig. S11).

Another approach to determining the mode of inhibition leverages the following relationships [50]:

$$v_{0}=\frac{V_{\max}\left[S\right]}{K_{m}\left(1+\frac{\left[I\right]}{K_{\mathrm{IC}}}\right)+\left[S\right]\left(1+\frac{\left[I\right]}{K_{\mathrm{IU}}}\right)}$$

$$\frac{1}{{\mathrm{IC}}_{50}}=\frac{1}{K_{\mathrm{IC}}}+\frac{v_{0}}{V_{\max}}\left(\frac{1}{K_{\mathrm{IU}}}-\frac{1}{K_{\mathrm{IC}}}\right)$$

Here, *K*_IU_ and *K*_IC_ are the uncompetitive and competitive inhibition constants, reflecting the two types of inhibition possible. When *K*_IU_ = *K*_IC_, it is an instance of noncompetitive inhibition. When 1/IC_50_ is plotted as a function of *v*_o_/*V*_max_, there should be no change in the case of noncompetitive and either negative or positive slopes with mixed‐uncompetitive or mixed‐competitive inhibitors, respectively. Our data revealed weakly positive (3470:A09, 3469:N17, 1533:K12), weakly negative (3469:F02), and no (1524:E12, 1524:G12) slopes in plots of 1/IC_50_ vs. *v*_o_/*V*_max_ (Fig. S12). The average (±SE) *K*_IU_/*K*_IC_ ratios for 1524:E12, 1524:G12, 1533:K12, 3470:A09, 3469:N17, and 3469:F02 are 0.95 ± 0.05, 0.9 ± 0.1, 0.75 ± 0.05, 0.7 + 0.1, 0.7 ± 0.1, and 1.35 ± 0.25, respectively. Taken together, our data suggest that all six compounds act predominantly as noncompetitive. While there are shades of mixed‐type uncompetitive (1533:K12, 3470:A09, 3469:N17) and mixed‐type competitive (3469:F02) characteristics, such a classification would be easier to justify if the 1/IC_50_ vs. *v*_o_/*V*_max_ trends were more pronounced (as exemplified in Ref. [52]). Caution with this sub‐classification is warranted given that the effects on *K*_m_ are modest with these last four compounds, a feature that is reflected in the α′ versus [I] plots and *K*_IU_/*K*_IC_ ratios (Fig. 5, Figs S11 and S12).

We were also intrigued by the finding that there were no competitive inhibitors of FraB among our top hits. Therefore, we conducted a Tanimoto search to identify structurally similar compounds in the ICCB‐Longwood libraries to 6‐P‐F‐Asp. Indeed, none of the compounds exhibited a strong structural resemblance to 6‐P‐F‐Asp, a finding that may account for the lack of competitive inhibitors in the small‐molecule libraries that we screened (data not shown). Nonetheless, we recognize that some of the compounds, not subject to further characterization by kinetic studies, from the 90 *bona fides* may be competitive inhibitors of FraB.

### Native MS analysis of FraB and FraB–inhibitor complexes

**Fig. 6:**
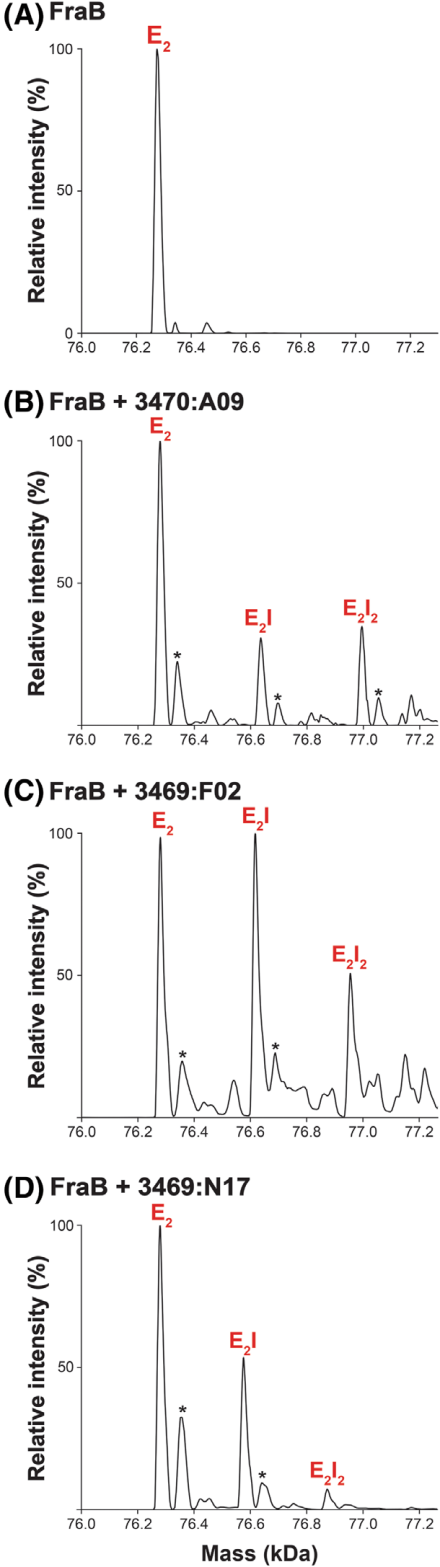
Deconvolved native mass spectra for FraB without (A) and with inhibitor 3470:A09 (B), 3469:F02 (C), and 3469:N17 (D) bound. E_2_, FraB dimer alone; E_2_I or E_2_I_2_, FraB dimer bound to one or two inhibitor copies, respectively. Asterisks (*) denote DMSO adducts (see Fig. S13 for additional information). Data were collected under IST 50 V. The primary native MS spectra corresponding to these data are in Fig. S14. These studies were performed with inhibitors purchased from ChemDiv; similar results were obtained when the compounds were from Ambinter (see Fig. S15).

To complement our kinetic analyses, we employed native MS to verify binding of the three triazolothiadiazoles (3470:A09, 3469:F02, and 3469:N17) to FraB. Native MS preserves the protein's native‐like structure and retains noncovalent interactions, enabling intact protein–protein and protein–ligand complexes to be transferred into the gas phase and characterized [61, 62]. We used native MS to characterize recombinant FraB ± DMSO (alone) or FraB ± inhibitors (in DMSO) (Fig. 6, Figs S13 and S14); while there were some expected DMSO adducts, the FraB‐bound species observed by nMS are within 2 Da of the theoretical masses (see Table S9 for a complete list of theoretical and experimental masses). The nMS data revealed the binding of the three inhibitors to FraB with distinct peaks corresponding to E_2_I and E_2_I_2_ (i.e., FraB dimer bound to one or two inhibitor copies, respectively) being observed (Fig. 6). The data were also similar regardless of whether the three triazolothiadiazoles were sourced from ChemDiv or Ambinter (Fig. 6, Figs S14–S16).

To understand the type of interaction (i.e., covalent *vs* noncovalent) between FraB and the three triazolothiadiazoles (3470:A09, 3469:F02, and 3469:N17), we activated the individual FraB–inhibitor (EI) complexes by increasing the IST voltage in the front end of the instrument when running the native MS experiment. We observed a strong decrease in E_2_I and E_2_I_2_ with increasing IST voltage, indicative of the noncovalent nature of FraB‐triazolothiadiazole inhibitor interactions (Fig. S17).

**Fig. 7:**
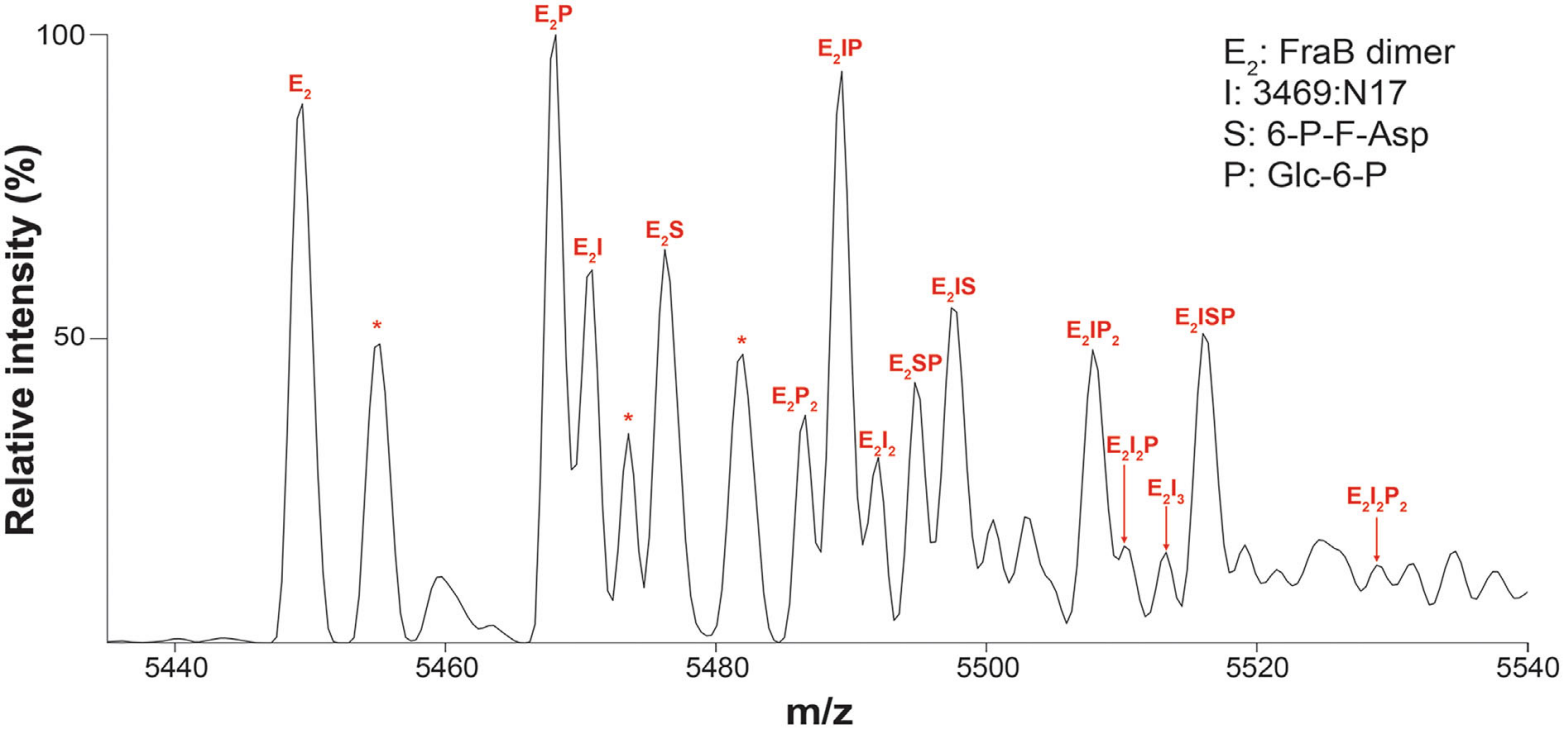
Native mass spectra for FraB bound to 6‐P‐F‐Asp and the 3469:N17 inhibitor. FraB, 6‐P‐F‐Asp, and 3469:N17 were used at final concentrations of 3 μm (monomer concentration), 600 μm, and 120 μm, respectively. Spectra were zoomed into the 14+ charge state. Asterisks (*) denote DMSO adducts (see Fig. S13). S – substrate (6‐P‐F‐Asp); P – product (Glc‐6‐P), one of the two products generated by FraB. These studies were performed with N17 purchased from ChemDiv; similar results were obtained when the compound was obtained from Ambinter (see Fig. S18).

To gain additional insights into the inhibition of FraB by triazolothiadiazoles, we tested the enzyme‐inhibitor‐substrate (EIS) complex, using 3469:N17 as an example, by adding 6‐P‐F‐Asp to the FraB‐3469:N17 complex before the native MS experiment. Our observation of masses corresponding to EIS complexes reveal the concomitant binding of substrate and inhibitor to FraB (Fig. 7, Fig. S18). As FraB functions as a dimer and has two active sites, the identification of E_2_IP_2_, E_2_I_2_P, E_2_ISP, and E_2_I_2_P_2_, where ‘P’ is product (Glc‐6‐P), suggests the binding of the inhibitor to functional FraB, albeit not at the active site. The enzyme–inhibitor–product complexes observed reflect incomplete inhibition of FraB despite the concentration of 3469:N17 (4× the *K*_I_) tested in this experiment. Regardless, these findings confirm that functional ES and EIS complexes in solution are indeed trapped in the gas phase during the nMS experiment. Moreover, we note that we detected FraB bound to Glc‐6‐P more often than l‐Asp, the second product of deglycation, an observation that likely reflects the weaker affinity of FraB to l‐Asp. Importantly, these native MS data confirm that 3469:N17, a triazolothiadiazole, can bind either free enzyme or the enzyme‐substrate complex, consistent with the mixed mode of inhibition discerned from our kinetic studies. The presence of EPI and ESPI complexes (Fig. 7) also illustrates how a continuum of states during catalysis can be detected by native MS.

Our studies with cyclopropyl‐substituted 1,2,4‐thiadiazolidine‐3,5‐dione and 1,2,4‐triazolidine‐3‐one variants (1524:E12, 1524:G12, and 1533:K12, all from Ambinter) revealed that these compounds bound the FraB dimer in stoichiometric ratios that ranged from 1 to 8 (not shown). The presence of 5 Cys residues per FraB monomer coupled to the thiol reactivity of these compounds (with one of them needing a tautomerization from a thione to thiol) explains this pattern[63, 64], but characterization of the sites of modification and covalent/noncovalent nature of each binding site is beyond the scope of the present study and is therefore not discussed here.

## Discussion

### Identification of *Salmonella* FraB inhibitors

From our biochemical‐based HTS, we identified nine triazolothiadiazoles that inhibit the activity of FraB *in vitro*, with at least six being mixed‐type inhibitors (Fig. 5, Figs S11 and S12). The recurrence of the triazolothiadiazole pharmacophore among our many hits suggests that it is a *bona fide* lead and that we have a starting point for at least one key chemotype that can be subject to structure‐based design, virtual screening, fragment screening, and farming for additional compounds with this core from commercial sources (‘analog by catalog’, ongoing). Assessment of the inhibitory potency of these new compounds in biochemical and cell‐based assays will permit structure–activity correlates to be drawn.

To check whether any of our top hits had been identified in previous HTS campaigns, we searched bioassay records in PubChem: 3469:N17 was reported to inhibit dCTP pyrophosphatase 1; 3470:A09 was found to inhibit deubiquitinases USP17, USP28, and USP30 [65]; and no targets were identified for 3469:F02. Triazolothiadiazoles have been evaluated for anti‐inflammatory, antibacterial, anticancer, and antifungal activities [66, 67, 68, 69]; such initiatives were inspired by the fact that these compounds are cyclic analogs of thio‐semicarbazides and biguanides, whose diverse biological activities are the basis for many drugs. Almajan *et al*. [66] examined a series of triazolothiadiazoles for their antibacterial potency against four gram‐negative (*Salmonella* was not tested) and five gram‐positive bacteria, and found these compounds to be less effective than ampicillin and to have low to moderate activity (50–100 mm, MIC). Another investigation that focused on heparanase inhibitors as anticancer drugs found that specific triazolothiadiazoles alleviated tumor (myeloma) growth in mice [67]; importantly, these compounds were not toxic to mice. Specific triazolothiadiazoles were also found to inhibit urease and thereby inhibit a pathogenic bacterium and fungus [69]. While our findings, coupled to those from earlier studies, may suggest a surprisingly broad class of biological targets for these compact and planar triazolothiadiazoles, the various electron donating/withdrawing substituents and structural differences suggest that non‐specific interactions could be minimized once we have a firm understanding of the structure–activity relationships in potent FraB inhibitors.

### Use of nMS to study inhibition of FraB by triazolothiadiazoles

The unambiguous identification of enzyme‐inhibitor species by native MS provides strong support for the selection of moderately tight binders from the HTS effort (Fig. 6). Importantly, the ability to observe distinct species of FraB in complex with substrate, product, and/or inhibitor in varying stoichiometries (Fig. 7) highlights the value of native MS to obtain mechanistic insights into inhibition of enzymes. Moreover, our ongoing studies to use MS/MS bottom‐up proteomics and top‐down electron capture dissociation MS for mapping the substrate/inhibitor‐binding sites in FraB (Gao, Y. & Wysocki, V. H., unpublished data) are expected to shed light on the binding sites of the different pharmacophores identified here but are outside the scope of this paper.

### Lack of overlaps between assays

There is a surprising lack of overlap in the hits obtained in the biochemical HTS (this study) and a cell‐based HTS campaign that we recently reported [55]. We offer some explanations for this discordance and group these reasons in two categories. In considering why cell‐based HTS identified winners not observed in the biochemical assay, it is possible that cellular metabolites (absent in our *in vitro* assay) may help facilitate the binding of select compounds to FraB inside the cell. Moreover, exposure of compounds to FraB posttranslationally (after folding) versus co‐translationally (during folding) may lead to different conformations and exposed surfaces that are conducive for binding a compound intracellularly but not *in vitro*. Lastly, compounds may have been subject to intracellular modifications that transform them into an inhibition‐competent/inhibition‐incompetent form, and such molecules will be scored as winners in only one format.

Different factors might account for why hits from the biochemical HTS did not surface in the cell‐based assay. Compound efficacy may be influenced by the pH of the bacterial growth medium, which will change over time in cell‐based assays. Additionally, prolonged (20 h) exposure to 37 °C in cell‐based assays may have led to compound degradation compared with *in vitro* assays with shorter durations (20 min). Drug‐efflux pump systems in *Salmonella* may exclude otherwise effective compounds from the cytoplasm or the test compound(s) did not possess ideal physicochemical properties for permeability. Finally, the redox status inside the cell may affect FraB structure or nullify potency of thiol‐reactive hits and lead to a discord with the *in vitro* findings.

### Limitations and future directions

While we are encouraged by the finding that the triazolothiadiazole scaffold offers a strong inhibitor candidate for development of an anti‐*Salmonella* therapeutic, we recognize that there are some obvious routes by which antibiotic resistance could arise when FraB is targeted (e.g., FraD, the kinase that generates 6‐P‐F‐Asp). However, we have proposed that targeting multiple metabolic pathways [36], each of which involves the generation of a different toxic sugar phosphate, will present a viable strategy to decrease the probability of antibiotic resistance from arising, to increase specificity, and to lower the effective concentration of each inhibitor that is required to disrupt a given pathway. Furthermore, co‐administration of inhibitors with probiotic strains of *Salmonella* lacking the *fra* operon may help to minimize resistance from arising given the competition for all nutrients except for F‐Asn (i.e., *Salmonella* strains lacking SPI1 SPI2 and the *fra* island [70]).

## Conflict of interest

The authors declare no conflict of interest.

## Author contributions

JDL designed and performed most of the experimental procedures (exceptions are noted below) and prepared the first draft of the entire manuscript. JDL and SK performed the initial screening of the small‐molecule libraries at Harvard, under the supervision of BMMA and VG. PT synthesized the substrate for the initial HTS and determined in pilot studies the IC_50_ and *K*_I_ values for some of the hits (data not shown). YG designed and performed all the native MS experiments. All authors edited and approved the final version of the manuscript.

## Supporting information


**Fig. S1.** SDS‐PAGE analysis of purified His_6_‐FraB wild‐type using a 15% (w/v) polyacrylamide gel.
**Fig. S2.** Standard curves for NADH relative florescence units (RFUs) versus concentration (μm) using either the Tecan Infinite M1000Pro, Tecan SPARK, or Synergy H1 Hybrid Reader instrument.
**Fig. S3.** Representative Michaelis–Menten plot showing the change in initial velocity of FraB‐G6PD as a function of 6‐P‐F‐Asp concentration with curve‐fit errors.
**Fig. S4.** Relative activity of FraB‐G6PD in the presence of 0‐50% (v/v) DMSO.
**Fig. S5.** Correlation plots (A) and residuals (B) between expected and observed initial velocities of FraB from two trials.
**Fig. S6.** Workflow for HTS of the small‐molecule libraries with a depiction of expected outcomes.
**Fig. S7.** Correlation plots showing the absorbances (from two independent trials) of wells containing either nothing (empty), G6PD (positive control), FraB and G6PD (negative control), or FraB and G6PD plus compounds from plates 3769–3772 of the Asinex1 library (test) before (left graphs) and after (right graphs) substrate addition.
**Fig. S8.** Representative HTS data for a subset of compounds screened at the ICCB‐Longwood Screening Facility showing the absorbance of test wells either before (green filled circles) or after (red filled circles) addition of substrate.
**Fig. S9.** Summary of outcomes at different stages of our HTS campaign.
**Fig. S10.** Determination of the IC_50_ values for six different inhibitors.
**Fig. S11.** Characterization of the inhibition of *Salmonella* FraB by 3470:A09 (A–D), 1524:G12 (F–H), 1533:K12 (I–L), 3469:N17 (M–P and Y–AB), 3469:F02 (Q–T and AC–AF), and 1524:E12 (U–X and AG–AJ).
**Fig. S12.** Determination of the *K*_IU_, *K*_IC_, and *K*_IU_/*K*_IC_ values for six different inhibitors.
**Fig. S13.** Primary (left) and deconvolved (right) native MS spectra for FraB in the absence (A) or presence of 4.8% (v/v) DMSO (B).
**Fig. S14.** Primary native MS spectra corresponding to the deconvolved spectra shown in Fig. 6 for FraB either alone (A) or bound to inhibitor 3470:A09 (B), 3469:F02 (C), and 3469:N17 (D).
**Fig. S15.** Deconvolved native mass spectra for FraB without (A) and with inhibitor 3470:A09 (B), 3469:F02 (C), and 3469:N17 (D) bound.
**Fig. S16.** Primary native MS spectra corresponding to the deconvolved spectra shown in Fig. S15 for FraB either alone (A) or bound to inhibitor 3470:A09 (B), 3469:F02 (C), and 3469:N17 (D).
**Fig. S17.** Deconvolved native mass spectra for FraB with the bound to 3470:A09 (A), 3469:F02 (B), and 3469:N17 (C) using in‐source trapping (IST) voltages of 50, 70 and 100 V.
**Fig. S18.** Native mass spectra for FraB bound to 6‐P‐F‐Asp and the 3469:N17 inhibitor.
**Table S1.** Curve‐fit parameters for IC_50_ plots shown in Fig. S10.
**Table S2.** Curve‐fit parameters for Michaelis–Menten graphs shown in Fig. 5 and Fig. S11.
**Table S3.** Curve‐fit parameters for “observed *v*_o_” versus “expected *v*_o_” plots shown in Fig. S5.
**Table S4.** Curve‐fit parameters for Lineweaver‐Burk plots shown in Fig. 5 and Fig. S11.
**Table S5.** Curve‐fit parameters for plots of α′ versus [I] shown in Fig. 5 and Fig. S11.
**Table S6.** Curve‐fit parameters for IC_50_ versus [S]/*K*_m_ plots shown in Fig. 5 and Fig. S11.
**Table S7.** Curve‐fit parameters for 1/IC_50_ versus *v*_o_/*V*_max_ plots featured in Fig. S12.
**Table S8.** List of commercially purchased compounds that are shaded red if they inhibit FraB‐G6PD by more than 30% using either 0.2 mm substrate and 0.25 mm inhibitor (A), 0.2 mm substrate and 0.025 mm inhibitor (B), or 1 mm substrate and 0.025 mm inhibitor (C) or inhibit G6PD by more than 20%, using 0.05 mm G‐6‐P and 0.25 mm inhibitor (D), as depicted in Fig. 4.
**Table S9.** Theoretical and experimental masses of FraB, FraB‐inhibitor complexes, and FraB‐inhibitor‐substrate complexes measured by nMS.

## Data Availability

Data are available upon request from Venkat Gopalan at gopalan.5@osu.edu.
