# Supplementary material for: Identification of inhibitors of the Salmonella FraB deglycase, a drug target
Source: FEBS Open Bio. 2025 Feb 13;15(5):773–92. doi: 10.1002/2211-5463.70001 (PMC12051030; doi:10.1002/2211-5463.70001)
Supplement: Supplementary file 1 — Fig. S1. SDS‐PAGE analysis of purified His6‐FraB wild‐type using a 15% (w/v) polyacrylamide gel. Fig. S2. Standard curves for NADH relative florescence units (RFUs) versus concentration (μm) using either the Tecan Infinite M1000Pro, Tecan SPARK, or Synergy H1 Hybrid Reader instrument. Fig. S3. Representative Michaelis–Menten plot showing the change in initial velocity of FraB‐G6PD as a function of 6‐P‐F‐Asp concentration with curve‐fit errors. Fig. S4. Relative activity of FraB‐G6PD in the presence of 0‐50% (v/v) DMSO. Fig. S5. Correlation plots (A) and residuals (B) between expected and observed initial velocities of FraB from two trials. Fig. S6. Workflow for HTS of the small‐molecule libraries with a depiction of expected outcomes. Fig. S7. Correlation plots showing the absorbances (from two independent trials) of wells containing either nothing (empty), G6PD (positive control), FraB and G6PD (negative control), or FraB and G6PD plus compounds from plates 3769–3772 of the Asinex1 library (test) before (left graphs) and after (right graphs) substrate addition. Fig. S8. Representative HTS data for a subset of compounds screened at the ICCB‐Longwood Screening Facility showing the absorbance of test wells either before (green filled circles) or after (red filled circles) addition of substrate. Fig. S9. Summary of outcomes at different stages of our HTS campaign. Fig. S10. Determination of the IC50 values for six different inhibitors. Fig. S11. Characterization of the inhibition of Salmonella FraB by 3470:A09 (A–D), 1524:G12 (F–H), 1533:K12 (I–L), 3469:N17 (M–P and Y–AB), 3469:F02 (Q–T and AC–AF), and 1524:E12 (U–X and AG–AJ). Fig. S12. Determination of the K IU, K IC, and K IU/K IC values for six different inhibitors. Fig. S13. Primary (left) and deconvolved (right) native MS spectra for FraB in the absence (A) or presence of 4.8% (v/v) DMSO (B). Fig. S14. Primary native MS spectra corresponding to the deconvolved spectra shown in Fig. 6 for FraB either alone (A) or [file FEB4-15-773-s001.pdf]

Identification of inhibitors of the *Salmonella* FraB deglycase, a drug target

Jamison D. Law[1], Yuan Gao[1,3,¶], Sravya Kovvali[2,¶],

Pankajavalli Thirugnanasambantham[1,¶], Vicki H. Wysocki[1,3,5],

Brian M. M. Ahmer[4], and Venkat Gopalan[1,5,*]

[1]Department of Chemistry and Biochemistry,

[2]Department of Microbiology,

[3]Native Mass Spectrometry Guided Structural Biology Center,

[4]Department of Microbial Infection and Immunity,

[5]Center for RNA Biology,

The Ohio State University, Columbus, OH 43210

¶ Co-second authors

* Correspondence to:

Venkat Gopalan

Department of Chemistry and Biochemistry

The Ohio State University

484 West 12th Avenue

Columbus, OH 43210

Tel: 614-292-1332

Email: gopalan.5@osu.edu

SUPPLEMENTARY TABLES

Table S1: Curve-fit parameters for IC_{50} plots shown in **Figure S10**.

Inhibitor	Trial	R	R squared	Chi Squared	Y max	Y max error	IC_{50}	IC_{50} error
1524:G12	1	0.999	0.998	37.5	103.3	1.38	3.24	0.08
	2	0.997	0.995	73.1	95.4	2.23	2.39	0.12
1524:E12	1	0.995	0.989	87.8	98.1	2.56	4.28	0.26
	2	0.995	0.990	79.7	95.7	2.37	4.29	0.24
3470:A09	1	0.998	0.996	32.9	99.2	1.70	15.84	0.67
	2	0.999	0.997	25.9	99.8	1.45	14.99	0.49
3469:N17	1	0.993	0.985	165.9	95.2	3.50	113.10	8.73
	2	0.995	0.989	79.0	97.9	2.50	86.68	5.47
3469:F02	1	0.996	0.993	62.9	98.4	2.29	44.45	2.95
	2	0.996	0.993	71.1	96.9	2.41	39.27	2.67
1533:K12	1	0.998	0.996	105.0	107.0	1.87	31.49	0.63
	2	0.999	0.998	28.1	99.3	1.75	23.67	0.29

Table S2: Curve-fit parameters for Michaelis-Menten graphs shown in **Figure 5** and **Figure S11**.

Compound	Trial	Inhibitor (μM)	R	R squared	Chi Squared	V_{max}	V_{max} error	K_m	K_m error
1524:E12	1	0	0.99534	0.99071	0.58386	6.5209	0.11849	40.928	3.9403
		2.5	0.99832	0.99665	0.093511	4.4919	0.047448	41.043	2.2958
		5	0.99281	0.98568	0.22448	3.1667	0.072092	37.025	4.5364
		10	0.99105	0.98219	0.092746	1.8028	0.04672	38.685	5.3586
	2	0	0.99341	0.98686	0.42798	4.656	0.097711	33.388	3.8334
		2.5	0.99164	0.98334	0.30753	3.406	0.082488	32.612	4.3374
		5	0.99255	0.98516	0.14752	2.5	0.058101	35.858	4.5079
		7.5	0.9873	0.97476	0.11697	1.6887	0.052206	37.662	6.2496
		10	0.99485	0.98974	0.027898	1.2817	0.029377	71.454	7.9342
3470:A09	1	0	0.9953	0.99063	0.17414	5.3249	0.074404	45.04	3.3955
		10	0.99631	0.99263	0.087304	4.0969	0.055041	55.601	3.8621
		20	0.98928	0.97868	0.093965	2.7851	0.053146	38.791	4.1228
		40	0.97536	0.95134	0.070283	1.4392	0.049939	58.437	10.382
	2	0	0.99682	0.99365	0.39464	7.6304	0.19293	48.424	4.7205
		10	0.99529	0.9906	0.16202	3.978	0.11576	40.159	4.6947
		20	0.99753	0.99506	0.029455	2.4156	0.047373	35.352	2.8635
		30	0.98458	0.9694	0.079852	1.4785	0.092962	57.831	13.471
3469:N17	1	0	0.99372	0.98748	0.52217	5.2188	0.11393	44.48	5.0779
		20	0.99486	0.98974	0.18971	3.5407	0.067205	39.875	4.0263
		40	0.99265	0.98535	0.10911	2.1788	0.049543	34.195	4.2378
		80	0.9905	0.98108	0.072836	1.5378	0.041903	41.182	5.9393
	2	0	0.99788	0.99577	0.18524	5.414	0.070275	52.47	3.4705
		20	0.9989	0.9978	0.039732	3.6013	0.030333	37.044	1.6791
		40	0.989	0.97813	0.22211	2.4591	0.073266	41.45	6.53
		80	0.97704	0.9546	0.19039	1.6245	0.074686	64.203	14.558

Table S2 (continued)

Compound	Trial	Inhibitor (μM)	R	R squared	Chi Squared	V_{max}	V_{max} error	K_m	K_m error
3469:F02	1	0	0.998	0.99601	0.15311	5.346	0.072628	42.061	2.7821
		10	0.99634	0.99269	0.21266	4.6218	0.083764	38.281	3.4334
		20	0.99379	0.98761	0.20325	3.378	0.087047	49.377	6.0245
		40	0.99329	0.98663	0.13612	2.5862	0.074169	57.395	7.5881
		60	0.98323	0.96673	0.13976	1.7154	0.070416	44.722	8.8436
	2	0	0.9814	0.96314	0.5278	5.7137	0.18191	32.086	5.0368
		25	0.97377	0.94824	0.44549	4.3838	0.16444	29.88	5.6361
		50	0.9537	0.90954	0.24686	2.5971	0.11773	24.806	5.9698
		150	0.95454	0.91114	0.024207	0.83859	0.036942	25.062	5.8432
1524:G12	1	0	0.99811	0.99622	0.12365	5.6267	0.061463	56.185	3.0935
		0.625	0.99329	0.98663	0.22398	3.9542	0.082907	56.733	5.9863
		1.25	0.99731	0.99462	0.066807	3.4606	0.045213	56.375	3.7104
		2.5	0.99034	0.98077	0.075731	1.8527	0.0499	65.504	8.6731
		3.75	0.98569	0.97159	0.046908	1.1972	0.037655	54.887	8.7336
	2	0	0.9923	0.98465	0.60609	6.978	0.18889	40.803	4.9248
		0.625	0.99473	0.98949	0.19985	4.9272	0.10731	39.28	3.843
		1.25	0.99592	0.99185	0.11003	4.1803	0.078368	37.052	3.1558
		1.5	0.99237	0.9848	0.10627	3.0181	0.075657	34.633	3.9966
		2	0.99586	0.99174	0.039819	2.5445	0.046973	36.555	3.0741
1533:K12	1	0	0.99357	0.98718	0.53595	5.2191	0.1116	35.393	4.0921
		5	0.99711	0.99424	0.14673	4.2436	0.058893	36.983	2.7556
		15	0.99604	0.9921	0.12539	3.3116	0.054449	37.002	3.266
		25	0.97763	0.95576	0.20786	1.8472	0.062948	19.351	3.9639
		30	0.97251	0.94577	0.25265	1.5454	0.082135	49.134	13.393
	2	0	0.99529	0.99061	0.33359	6.595	0.14283	43.592	4.1557
		10	0.9906	0.98128	0.43107	5.2895	0.15885	40.397	5.4198
		30	0.97286	0.94646	0.23152	2.4135	0.10502	26.856	5.6576
		40	0.9603	0.92218	0.11854	1.3625	0.075823	27.931	7.4658

Table S3: Curve-fit parameters for “observed v_o” versus “expected v_o” plots shown in **Figure S5**.

Compound	Trial	R	R squared	Adjusted R squared	Standard error	F value	P value	Y intercept	Y intercept error	Y intercept t value
1524:E12	1	0.99714	0.99428	0.99415	0.13645	7302.3	9.82E-49	0.049167	0.038967	1.2618
	2	0.997	0.99401	0.9939	0.098299	8799.2	1.37E-60	0.044218	0.02448	1.8063
1524:G12	1	0.99796	0.99592	0.99584	0.09977	12934	5.29E-65	0.02182	0.023229	0.93937
	2	0.9873	0.97476	0.97429	0.27831	2047	4.98E-44	0.040013	0.078095	0.51236
1533:K12	1	0.99615	0.99231	0.99216	0.12587	6835.6	1.05E-57	0.056016	0.03279	1.7083
	2	0.99581	0.99164	0.99139	0.17303	4031.3	6.54E-37	0.08688	0.049186	1.7663
3470:A09	1	0.98142	0.96319	0.96272	0.29903	2041.1	1.08E-57	0.58484	0.059617	9.81
	2	0.99842	0.99684	0.99673	0.11031	9460.5	4.55E-39	0.033082	0.030779	1.0749
3469:N17	1	0.99826	0.99652	0.99642	0.085329	10870	2.56E-48	0.032099	0.024522	1.309
	2	0.99826	0.99651	0.99642	0.086439	10863	2.59E-48	0.010995	0.025028	0.43928
3469:F02	1	0.99718	0.99438	0.99426	0.11058	8486	1.15E-55	0.050728	0.029879	1.6978
	2	0.99427	0.98858	0.98831	0.17688	3722.3	2.11E-43	0.059329	0.041882	1.4166

Compound	Trial	Y intercept p value	Y intercept lower 95%	Y intercept upper 95%	Slope	Slope error	Slope t value	Slope p value	Slope lower 95%	Slope upper 95%
1524:E12	1	2.14E-01	-0.02947	0.12781	0.98601	0.011539	85.454	9.82E-49	0.96273	1.0093
	2	7.65E-02	-0.00488	0.093318	0.98229	0.010472	93.804	1.37E-60	0.96129	1.0033
1524:G12	1	3.52E-01	-0.02477	0.068411	0.99195	0.008722	113.73	5.29E-65	0.97445	1.0094
	2	6.11E-01	-0.11663	0.19665	1.018	0.0225	45.244	4.98E-44	0.97287	1.0631
1533:K12	1	9.34E-02	-0.00975	0.12178	0.98055	0.01186	82.678	1.05E-57	0.95676	1.0043
	2	8.63E-02	-0.01308	0.18684	0.97501	0.015356	63.492	6.54E-37	0.9438	1.0062
3470:A09	1	2.93E-15	0.46616	0.70353	0.99014	0.021916	45.178	1.08E-57	0.94651	1.0338
	2	2.91E-01	-0.02978	0.095941	0.99023	0.010181	97.265	4.55E-39	0.96944	1.011
3469:N17	1	1.98E-01	-0.01754	0.081742	0.98855	0.009482	104.26	2.56E-48	0.96936	1.0077
	2	6.63E-01	-0.03967	0.061662	0.99545	0.009551	104.23	2.59E-48	0.97611	1.0148
3469:F02	1	9.60E-02	-0.00935	0.1108	0.98297	0.010671	92.12	1.15E-55	0.96152	1.0044
	2	1.64E-01	-0.02513	0.14379	0.97776	0.016026	61.011	2.11E-43	0.94544	1.0101

Table S4: Curve-fit parameters for Lineweaver-Burk plots shown in **Figure 5** and **Figure S11**.

Compound	Trial	Inhibitor (μM)	R	R squared	Adjusted R squared	Standard error	F value	P value	Y intercept	Y intercept error
1524:E12	1	0	0.99944	0.99887	0.99875	0.010331	7974.8	1.40E-14	0.14681	0.0036588
		2.5	0.99534	0.99071	0.98968	0.036434	959.68	1.87E-10	0.2279	0.012904
		5	0.99443	0.9889	0.98767	0.069316	801.7	4.16E-10	0.28612	0.02455
		10	0.99634	0.99269	0.99187	0.095544	1221.4	6.35E-11	0.51934	0.033839
	2	0	0.99551	0.99105	0.98993	0.016579	885.55	1.76E-09	0.20854	0.0062936
		2.5	0.99877	0.99754	0.99724	0.010907	3250.3	9.95E-12	0.28923	0.0041406
		5	0.99624	0.99249	0.99155	0.030034	1056.5	8.75E-10	0.38698	0.011401
		7.5	0.99612	0.99225	0.99128	0.043442	1024.4	9.89E-10	0.58732	0.016491
		10	0.99686	0.99373	0.99294	0.1149	1267.7	4.24E-10	0.71841	0.043617
3470:A09	1	0	0.98886	0.97784	0.97507	0.03677	352.95	6.66E-08	0.17076	0.013959
		10	0.99667	0.99335	0.99251	0.025174	1194.1	5.38E-10	0.24338	0.0095564
		20	0.9973	0.99461	0.99393	0.023197	1475.6	2.32E-10	0.35483	0.0088059
		40	0.99735	0.99471	0.99405	0.057311	1505.5	2.14E-10	0.71337	0.021756
	2	0	0.98775	0.97565	0.97294	0.053822	360.59	1.43E-08	0.1317	0.019072
		10	0.99052	0.98113	0.97903	0.066055	467.97	4.54E-09	0.26872	0.023407
		20	0.98583	0.97185	0.96873	0.10011	310.76	2.76E-08	0.43688	0.035473
		30	0.99887	0.99774	0.99748	0.076492	3966.1	3.24E-13	0.72063	0.027105
3469:N17	1	0	0.99745	0.9949	0.99426	0.015238	1559.6	1.86E-10	0.18426	0.0057845
		20	0.9976	0.9952	0.9946	0.016855	1657.6	1.46E-10	0.28269	0.0063986
		40	0.99806	0.99612	0.99563	0.022821	2053.6	6.21E-11	0.45036	0.0086631
		80	0.99693	0.99387	0.9931	0.04051	1296.6	3.88E-10	0.66696	0.015378
	2	0	0.99957	0.99913	0.99903	0.011659	10350	4.35E-15	0.18892	0.0041292
		20	0.9997	0.9994	0.99933	0.009901	14894	8.46E-16	0.28197	0.0035067
		40	0.98769	0.97553	0.97281	0.16855	358.8	1.47E-08	0.32606	0.059695
		80	0.99527	0.99056	0.98951	0.1559	944.68	2.00E-10	0.58958	0.055215

Table S4 (continued)

Compound	Trial	Inhibitor (μM)	Y intercept t value	Y intercept p value	Y intercept lower 95%	Y intercept upper 95%	Slope	Slope error	Slope t value	Slope p value	Slope lower 95%	Slope upper 95%
1524:E12	1	0	40.123	1.85E-11	0.13853	0.15508	7.351	0.082318	89.302	1.40E-14	7.1649	7.5374
		2.5	17.661	2.71E-08	0.19871	0.25709	8.994	0.29032	30.979	1.87E-10	8.337	9.6505
		5	11.655	9.87E-07	0.23058	0.34166	15.64	0.55234	28.314	4.16E-10	14.39	16.888
		10	15.347	9.25E-08	0.44279	0.59589	26.61	0.76133	34.948	6.35E-11	24.885	28.329
	2	0	33.136	7.51E-10	0.19403	0.22306	8.044	0.27031	29.758	1.76E-09	7.4206	8.6673
		2.5	69.851	1.96E-12	0.27968	0.29877	10.14	0.17784	57.011	9.95E-12	9.7288	10.549
		5	33.942	6.20E-10	0.36069	0.41327	15.92	0.48969	32.505	8.75E-10	14.788	17.046
		7.5	35.614	4.23E-10	0.54929	0.62534	22.67	0.7083	32.007	9.89E-10	21.037	24.304
		10	16.471	1.86E-07	0.61783	0.81899	66.7	1.8734	35.604	4.24E-10	62.38	71.02
3470:A09	1	0	12.233	1.85E-06	0.13857	0.20294	11.26	0.59953	18.787	6.66E-08	9.8807	12.646
		10	25.467	6.06E-09	0.22134	0.26541	14.18	0.41045	34.556	5.38E-10	13.237	15.13
		20	40.294	1.58E-10	0.33452	0.37513	14.53	0.37822	38.414	2.32E-10	13.657	15.401
		40	32.789	8.16E-10	0.6632	0.76354	36.26	0.93443	38.801	2.14E-10	34.102	38.411
	2	0	6.9056	7.02E-05	0.088558	0.17485	8.148	0.42908	18.989	1.43E-08	7.1772	9.1185
		10	11.48	1.12E-06	0.21577	0.32167	11.39	0.52661	21.633	4.54E-09	10.201	12.583
		20	12.316	6.17E-07	0.35663	0.51712	14.07	0.79808	17.628	2.76E-08	12.263	15.874
		30	26.587	7.28E-10	0.65932	0.78195	38.4	0.6098	62.977	3.24E-13	37.024	39.783
3469:N17	1	0	31.855	1.03E-09	0.17092	0.1976	9.812	0.24845	39.492	1.86E-10	9.2388	10.385
		20	44.181	7.60E-11	0.26794	0.29745	11.19	0.27482	40.714	1.46E-10	10.555	11.823
		40	51.985	2.08E-11	0.43038	0.47033	16.86	0.37209	45.317	6.21E-11	16.004	17.72
		80	43.37	8.81E-11	0.6315	0.70242	23.78	0.66051	36.009	3.88E-10	22.261	25.307
	2	0	45.753	5.69E-12	0.17958	0.19826	9.451	0.092899	101.74	4.35E-15	9.2409	9.6612
		20	80.408	3.60E-14	0.27404	0.2899	9.629	0.078896	122.04	8.46E-16	9.45	9.807
		40	5.4622	0.00039928	0.19103	0.4611	25.44	1.343	18.942	1.47E-08	22.402	28.478
		80	10.678	2.07E-06	0.46468	0.71449	38.18	1.2422	30.736	2.00E-10	35.371	40.991

Table S4 (continued)

Compound	Trial	Inhibitor (μM)	R	R squared	Adjusted R squared	Standard error	F value	P value	Y intercept	Y intercept error
3469:F02	1	0	0.98843	0.977	0.97372	0.013945	297.4	5.42E-07	0.18971	0.0057452
		10	0.9876	0.97536	0.97184	0.014724	277.06	6.90E-07	0.22086	0.0060661
		20	0.99176	0.98358	0.98124	0.021593	419.33	1.66E-07	0.29711	0.0088963
		40	0.9986	0.9972	0.9968	0.012713	2493.6	3.38E-10	0.38636	0.0052376
		60	0.91273	0.83307	0.79134	0.052537	19.962	1.11E-02	0.57009	0.031715
	2	0	0.99038	0.98085	0.97872	0.039649	460.87	4.86E-09	0.18172	0.01405
		25	0.99383	0.98769	0.98633	0.048845	722.39	6.61E-10	0.20936	0.017308
		50	0.99162	0.9833	0.98145	0.081855	530.08	2.61E-09	0.34964	0.029005
		100	0.99726	0.99453	0.99392	0.20172	1636.7	1.72E-11	0.74606	0.07148
		150	0.98005	0.96049	0.95555	0.15919	194.49	6.77E-07	1.1287	0.060499
1524:G12	1	0	0.99587	0.99176	0.99058	0.010584	842.64	1.48E-08	0.17641	0.0043605
		0.625	0.99617	0.99236	0.99126	0.014805	908.72	1.14E-08	0.25058	0.0060998
		1.25	0.99744	0.99489	0.99416	0.012913	1362.2	2.79E-09	0.29043	0.0053203
		2.5	0.99327	0.98659	0.98468	0.04208	515.03	8.17E-08	0.54632	0.017337
		3.75	0.98748	0.97512	0.97156	0.086555	274.31	7.14E-07	0.81955	0.035661
	2	0	0.98197	0.96426	0.95915	0.012955	188.85	2.55E-06	0.14688	0.0053515
		0.625	0.98284	0.96597	0.9603	0.0189	170.31	1.25E-05	0.20359	0.0085318
		1.25	0.9905	0.98109	0.97839	0.013524	363.19	2.73E-07	0.24398	0.0055866
		1.5	0.98094	0.96224	0.95685	0.024971	178.39	3.09E-06	0.33123	0.010315
		2	0.98534	0.97089	0.96673	0.029952	233.48	1.24E-06	0.3916	0.012373
1533:K12	1	0	0.9989	0.99781	0.99749	0.018108	3186.3	1.44E-10	0.17361	0.0073977
		5	0.99913	0.99827	0.99802	0.017587	4031.1	6.32E-11	0.23039	0.007185
		15	0.99908	0.99817	0.99791	0.023467	3814.6	7.66E-11	0.29665	0.0095873
		25	0.99625	0.99251	0.99144	0.047308	928.06	1.06E-08	0.50864	0.019327
		30	0.95196	0.90624	0.8828	0.050385	38.66	0.003406	0.63822	0.029335
	2	0	0.99869	0.99738	0.99694	0.008821	2283.2	5.63E-09	0.14649	0.0039535
		10	0.99712	0.99425	0.99329	0.015721	1036.8	5.96E-08	0.18002	0.0070458
		30	0.98546	0.97114	0.96633	0.058512	201.87	7.60E-06	0.38619	0.026224
		40	0.97363	0.94795	0.93928	0.16283	109.28	4.49E-05	0.65463	0.072979

Table S4 (continued)

Compound	Trial	Inhibitor (µM)	Y intercept t value	Y intercept p value	Y intercept lower 95%	Y intercept upper 95%	Slope	Slope error	Slope t value	Slope p value	Slope lower 95%	Slope upper 95%
3469:F02	1	0	33.021	6.05E-09	0.17613	0.2033	8.048	0.4667	17.245	5.42E-07	6.9448	9.1519
		10	36.408	3.06E-09	0.20651	0.2352	8.202	0.49277	16.645	6.90E-07	7.037	9.3674
		20	33.397	5.59E-09	0.27608	0.31815	14.8	0.72268	20.478	1.66E-07	13.09	16.508
		40	73.766	2.21E-11	0.37397	0.39874	21.25	0.42547	49.936	3.38E-10	20.24	22.252
		60	17.975	5.63E-05	0.48204	0.65815	37.21	8.3276	4.4679	1.11E-02	14.086	60.327
	2	0	12.934	4.06E-07	0.14994	0.21351	6.786	0.31609	21.468	4.86E-09	6.0707	7.5008
		25	12.096	7.19E-07	0.17021	0.24852	10.47	0.38941	26.877	6.61E-10	9.5853	11.347
		50	12.055	7.41E-07	0.28403	0.41526	15.02	0.65256	23.024	2.61E-09	13.548	16.5
		100	10.437	2.50E-06	0.58436	0.90776	65.06	1.6082	40.456	1.72E-11	61.421	68.697
		150	18.656	7.03E-08	0.98917	1.2682	36.24	2.5984	13.946	6.77E-07	30.245	42.229
1524:G12	1	0	40.456	1.47E-09	0.1661	0.18672	10.28	0.35421	29.028	1.48E-08	9.4447	11.12
		0.625	41.079	1.32E-09	0.23615	0.265	14.94	0.49551	30.145	1.14E-08	13.766	16.109
		1.25	54.59	1.81E-10	0.27785	0.30301	15.95	0.43218	36.908	2.79E-09	14.929	16.973
		2.5	31.512	8.37E-09	0.50533	0.58732	31.96	1.4083	22.694	8.17E-08	28.631	35.292
		3.75	22.982	7.49E-08	0.73523	0.90388	47.98	2.8969	16.562	7.14E-07	41.129	54.829
	2	0	27.447	2.19E-08	0.13423	0.15954	5.973	0.43467	13.742	2.55E-06	4.9455	7.0011
		0.625	23.862	3.56E-07	0.18271	0.22446	8.527	0.65341	13.05	1.25E-05	6.9282	10.126
		1.25	43.672	8.62E-10	0.23077	0.25719	8.648	0.45376	19.058	2.73E-07	7.5746	9.7205
		1.5	32.111	7.35E-09	0.30684	0.35562	11.19	0.83784	13.356	3.09E-06	9.2092	13.172
		2	31.649	8.12E-09	0.36234	0.42086	15.36	1.005	15.28	1.24E-06	12.98	17.733
1533:K12	1	0	23.468	6.48E-08	0.15611	0.1911	8.498	0.15055	56.448	1.44E-10	8.1421	8.8541
		5	32.065	7.42E-09	0.2134	0.24738	9.284	0.14622	63.491	6.32E-11	8.9378	9.6293
		15	30.942	9.51E-09	0.27398	0.31932	12.05	0.19511	61.762	7.66E-11	11.589	12.512
		25	26.317	2.93E-08	0.46294	0.55434	11.98	0.39332	30.464	1.06E-08	11.052	12.912
		30	21.756	2.64E-05	0.55677	0.71967	24.13	3.8811	6.2177	0.003406	13.356	34.907
	2	0	37.052	2.58E-08	0.13681	0.15616	7.258	0.15188	47.783	5.63E-09	6.8858	7.6291
		10	25.55	2.37E-07	0.16278	0.19727	8.716	0.27068	32.2	5.96E-08	8.0536	9.3783
		30	14.727	6.16E-06	0.32202	0.45036	14.31	1.0074	14.208	7.60E-06	11.849	16.779
		40	8.9701	0.00010726	0.47605	0.8332	29.31	2.8036	10.454	4.49E-05	22.449	36.169

Table S5: Curve-fit parameters for plots of α' versus [I] shown in **Figure 5** and **Figure S11**.

Compound	Trial	Kinetic parameter	R	R squared	Adjusted R squared	Standard error	F value	P value	Y intercept	Y intercept error	Y intercept t value
1524:E12	1	k_{cat}	0.99347	0.98698	0.98046	0.16068	151.57	6.53E-03	0.8439	0.12447	6.7802
		K_m	0.6809	0.46362	0.19544	0.067131	1.7287	3.19E-01	0.96809	0.052	18.617
	2	k_{cat}	0.98604	0.97227	0.96302	0.20521	105.17	1.98E-03	0.79336	0.15895	4.9912
		K_m	0.78798	0.62092	0.49455	0.14383	4.9138	1.13E-01	1.0373	0.11141	9.3106
1524:G12	1	k_{cat}	0.9877	0.97555	0.9674	0.27889	119.71	1.63E-03	0.7565	0.1955	3.8696
		K_m	0.58383	0.34086	0.12115	0.066174	1.5514	3.01E-01	0.96605	0.046387	20.826
	2	k_{cat}	0.96212	0.92568	0.90091	0.22644	37.366	8.80E-03	0.89019	0.18625	4.7795
		K_m	0.83258	0.69318	0.59091	0.044048	6.7779	8.01E-02	1.0071	0.036231	27.798
1533:K12	1	k_{cat}	0.95399	0.91009	0.86513	0.62786	20.244	4.60E-02	0.64073	0.5062	1.2658
		K_m	0.94726	0.89731	0.84596	0.1261	17.476	5.27E-02	0.98228	0.10167	9.6616
	2	k_{cat}	0.96964	0.94021	0.92027	0.28384	47.172	6.32E-03	0.82201	0.20977	3.9187
		K_m	0.097222	0.009452	-0.32073	0.3413	0.028627	8.76E-01	0.99686	0.25222	3.9523
3470:A09	1	k_{cat}	0.98643	0.97304	0.95956	0.35615	72.177	1.36E-02	0.76358	0.29798	2.5625
		K_m	0.18081	0.032693	-0.45096	0.27921	0.067596	8.19E-01	1.1508	0.2336	4.9265
	2	k_{cat}	0.97913	0.9587	0.93805	0.31868	46.424	2.09E-02	0.74111	0.24685	3.0023
		K_m	0.11156	0.012446	-0.48133	0.21487	0.025205	8.88E-01	1.0205	0.16644	6.1316
3469:N17	1	k_{cat}	0.99542	0.99087	0.9863	0.12683	217	4.58E-03	0.97368	0.098243	9.9109
		K_m	0.25462	0.06483	-0.40275	0.15521	0.13865	7.45E-01	1.0919	0.12023	9.0816
	2	k_{cat}	0.99852	0.99705	0.99558	0.077044	676.38	1.48E-03	0.93611	0.059678	15.686
		K_m	0.21404	0.045813	-0.43128	0.28914	0.096025	7.86E-01	1.2582	0.22397	5.6178
3469:F02	1	k_{cat}	0.98895	0.97802	0.9707	0.12988	133.5	1.39E-03	0.91402	0.09104	10.04
		K_m	0.86141	0.74202	0.65603	0.11268	8.6289	6.06E-02	1.0263	0.078987	12.993
	2	k_{cat}	0.98926	0.97864	0.97152	0.41613	137.46	1.33E-03	0.51438	0.2917	1.7634
		K_m	0.038563	0.001487	-0.33135	0.38426	0.004468	9.51E-01	0.99884	0.26936	3.7081

Table S5 (continued)

Compound	Trial	Kinetic parameter	Y intercept p value	Y intercept lower 95%	Y intercept upper 95%	Slope	Slope error	Slope t value	Slope p value	Slope lower 95%	Slope upper 95%
1524:E12	1	k_{cat}	2.11E-02	0.30836	1.3794	0.26751	0.021728	12.311	6.53E-03	0.17402	0.361
		K_m	2.87E-03	0.74435	1.1918	0.011936	0.0090778	1.3148	3.19E-01	-0.027123	0.050994
	2	k_{cat}	1.55E-02	0.28751	1.2992	0.26619	0.025957	10.255	1.98E-03	0.18359	0.3488
		K_m	2.62E-03	0.68272	1.3918	-0.040328	0.018193	-2.2167	1.13E-01	-0.098225	0.017569
1524:G12	1	k_{cat}	3.05E-02	0.13434	1.3787	1.0136	0.092642	10.941	1.63E-03	0.71879	1.3084
		K_m	2.42E-04	0.81842	1.1137	0.02738	0.021982	1.2455	3.01E-01	-0.042577	0.097336
	2	k_{cat}	1.74E-02	0.29746	1.4829	0.88886	0.14541	6.1127	8.80E-03	0.42609	1.3516
		K_m	1.02E-04	0.89182	1.1224	0.073641	0.028286	2.6034	8.01E-02	-0.016378	0.16366
1533:K12	1	k_{cat}	3.33E-01	-1.5373	2.8187	0.089333	0.019855	4.4993	4.60E-02	0.003905	0.17476
		K_m	1.05E-02	0.54483	1.4197	0.016671	0.0039878	4.1804	5.27E-02	-0.000487	0.033829
	2	k_{cat}	2.96E-02	0.15444	1.4896	0.076465	0.011133	6.8682	6.32E-03	0.041034	0.1119
		K_m	2.89E-02	0.19417	1.7996	0.002265	0.013387	0.16919	8.76E-01	-0.040338	0.044867
3470:A09	1	k_{cat}	1.24E-01	-0.51852	2.0457	0.13532	0.015928	8.4957	1.36E-02	0.066786	0.20385
		K_m	3.88E-02	0.14574	2.1559	-0.0032464	0.012486	-0.25999	8.19E-01	-0.056971	0.050478
	2	k_{cat}	9.53E-02	-0.32099	1.8032	0.073405	0.010773	6.8136	2.09E-02	0.027051	0.11976
		K_m	2.56E-02	0.3044	1.7367	-0.0011532	0.0072641	-0.15876	8.88E-01	-0.032408	0.030102
3469:N17	1	k_{cat}	1.00E-02	0.55097	1.3964	0.031581	0.0021438	14.731	4.58E-03	0.022357	0.040805
		K_m	1.19E-02	0.57456	1.6092	0.0009769	0.0026236	0.37236	7.45E-01	-0.010311	0.012265
	2	k_{cat}	4.04E-03	0.67934	1.1929	0.033869	0.0013023	26.007	1.48E-03	0.028266	0.039472
		K_m	3.03E-02	0.29455	2.2219	-0.0015145	0.0048874	-0.30988	7.86E-01	-0.022543	0.019514
3469:F02	1	k_{cat}	2.10E-03	0.62429	1.2038	0.031155	0.0026964	11.554	1.39E-03	0.022574	0.039736
		K_m	9.84E-04	0.77493	1.2777	-0.0068719	0.0023394	-2.9375	6.06E-02	-0.014317	0.000573
	2	k_{cat}	0.17603	-0.41394	1.4427	0.040517	0.0034557	11.724	1.33E-03	0.029519	0.051515
		K_m	3.41E-02	0.1416	1.8561	0.0002133	0.0031911	0.066843	9.51E-01	-0.009942	0.010369

Table S6: Curve-fit parameters for IC_{50} versus [S]/K_m plots shown in **Figure 5** and **Figure S11**.

		6-P-F-Asp (μM)	S/K_m	R	R squared	Chi Squared	Y max	Y max error	IC_{50}	IC_{50} error
1524:E12	Trial 2	7.38	0.175714	0.98978	0.97966	111.74	100.39	7.3969	4.2249	0.55407
		15.7	0.37381	0.99327	0.98658	61.446	99.096	5.4311	5.1146	0.50442
		31.3	0.745238	0.99393	0.9879	51.75	99.095	5.0368	4.8438	0.48764
		62.5	1.488095	0.99311	0.98627	54.009	99.088	5.1459	5.1187	0.54125
		125	2.97619	0.99772	0.99544	17.389	99.496	2.9256	5.0178	0.30988
		250	5.952381	0.99892	0.99785	8.227	99.582	2.0051	5.2509	0.21173
		500	11.90476	0.99784	0.99569	15.779	99.643	2.7924	4.9663	0.30748
		750	17.85714	0.99748	0.99497	17.43	99.633	2.9405	4.9173	0.34549
		1000	23.80952	0.99762	0.99524	16.856	99.7	2.8862	5.0782	0.32814
		2000	47.61905	0.99797	0.99595	14.125	99.662	2.641	5.2035	0.30692
		3000	71.42857	0.9999	0.9998	0.6312	100.02	0.55673	5.7824	0.069793
	Trial 1	7.38	0.175714	0.99304	0.98613	44.328	100.99	6.5844	5.182	0.73474
		15.7	0.37381	0.99993	0.99986	0.41881	100.05	0.64552	4.4596	0.076114
		31.3	0.745238	0.99221	0.98448	36.826	99.321	6.0303	6.2505	1.0134
		62.5	1.488095	0.99989	0.99978	0.58118	99.942	0.76013	5.0417	0.10529
		125	2.97619	0.9987	0.99741	7.3962	99.847	2.7153	4.4301	0.35335
		250	5.952381	0.99992	0.99984	0.46896	100.04	0.68343	4.497	0.085863
		500	11.90476	0.99978	0.99956	1.337	99.912	1.1529	4.5819	0.13774
		750	17.85714	0.99941	0.99882	3.4609	99.897	1.8575	4.2959	0.23201
		1000	23.80952	0.99985	0.9997	0.85139	99.934	0.92032	4.7019	0.11741
		2000	47.61905	0.99982	0.99964	0.99057	100.07	0.99263	4.852	0.13193
		3000	71.42857	0.99997	0.99993	0.17585	99.955	0.41689	5.5036	0.057891

Table S6 (continued)

		6-P-F-Asp (μM)	S/K_m	R	R squared	Chi Squared	Y max	Y max error	IC_{50}	IC_{50} error
3470:A09	Trial 1	7.38	0.175714	0.99304	0.98613	44.328	100.99	6.5844	5.182	0.73474
		15.7	0.37381	0.98855	0.97723	59.458	97.217	7.1911	29.282	4.2453
		31.3	0.745238	0.97013	0.94116	166.02	99.341	12.868	18.847	7.6794
		62.5	1.488095	0.99414	0.98832	34.442	99.39	5.8369	20.534	2.9648
		125	2.97619	0.99623	0.99246	23.772	99.404	4.8391	20.217	2.2577
		250	5.952381	0.99995	0.9999	0.31521	99.928	0.55677	20.55	0.26044
		500	11.90476	0.99935	0.9987	4.0687	99.787	2.0058	19.392	0.91963
		750	17.85714	1	1	0.012443	100.01	0.11092	19.525	0.051238
		1000	23.80952	0.99878	0.99756	7.4434	99.616	2.7011	21.324	1.3023
		2000	47.61905	0.99971	0.99942	1.5447	100.15	1.2333	22.728	0.68378
		3000	71.42857	0.99999	0.99998	0.057417	99.96	0.23618	23.223	0.12204
	Trial 2	7.38	0.175714	0.96273	0.92684	226.35	98.552	14.975	19.025	5.7862
		15.7	0.37381	0.99881	0.99762	9.3338	99.851	3.0515	13.641	0.8514
		31.3	0.745238	0.99034	0.98078	69.408	99.768	8.3279	12.397	2.8368
		62.5	1.488095	0.99667	0.99335	24.727	99.84	4.97	12.575	1.5547
		125	2.97619	0.99895	0.99791	8.089	99.927	2.8432	11.429	0.87459
		250	5.952381	0.99942	0.99884	4.4576	99.943	2.1105	11.638	0.64659
		500	11.90476	0.99954	0.99907	3.5105	99.959	1.8732	10.962	0.6132
		750	17.85714	0.99896	0.99792	7.6422	99.944	2.7639	10.862	0.96566
		1000	23.80952	0.99813	0.99626	13.203	99.925	3.6329	11.293	1.3251
		2000	47.61905	0.99644	0.99289	24.897	99.905	4.9888	11.072	1.898
		3000		0.97803	0.95654	117.28	99.749	10.827	15.337	5.5682

Table S6: Continued.

		6-P-F-Asp (μM)	S/K_m	R	R squared	Chi Squared	Y max	Y max error	IC_{50}	IC_{50} error
3469:N17	Trial 1	7.38	0.175714	0.99047	0.98104	77.642	102.16	5.9681	47.91	4.751
		15.7	0.37381	0.97772	0.95593	147.66	101.09	8.5185	45.364	8.2925
		31.3	0.745238	0.98819	0.97651	88.045	100.76	6.5811	40.868	5.6142
		62.5	1.488095	0.99354	0.98711	45.838	100.11	4.7776	34.873	4.3552
		125	2.97619	0.99462	0.98926	40.19	100.18	4.4747	32.673	3.8262
		250	5.952381	0.99193	0.98392	61.9	100.4	5.5482	34.063	4.5576
		500	11.90476	0.99362	0.98728	47.291	100.13	4.8576	30.087	4.2813
		750	17.85714	0.99227	0.9846	59.683	100.44	5.4491	33.432	4.4329
		1000	23.80952	0.99139	0.98285	70.173	100.53	5.9068	32.658	4.4961
		2000	47.61905	0.99492	0.98986	37.301	100.3	4.3117	32.454	3.7589
		3000	71.42857	0.99124	0.98256	56.767	100.44	5.3131	39.278	5.3307
	Trial 2	7.38	0.175714	0.96727	0.93562	281.3	103.35	16.434	36.615	10.199
		15.7	0.37381	0.98211	0.96455	120.62	102.46	10.704	44.803	9.3176
		31.3	0.745238	0.99755	0.99511	13.368	100.63	3.6017	48.233	3.8955
		62.5	1.488095	0.99999	0.99998	0.059751	99.962	0.24145	48.453	0.27352
		125	2.97619	0.99938	0.99875	3.6423	100.15	1.9025	37.726	1.8655
		250	5.952381	0.99997	0.99993	0.21591	99.968	0.46356	34.044	0.41162
		500	11.90476	1	1	0.007587	99.995	0.086985	32.883	0.083706
		750	17.85714	0.99989	0.99979	0.61743	100.05	0.7842	35.938	0.7882
		1000	23.80952	0.99944	0.99889	3.3656	100.12	1.8305	34.853	1.7168
		2000	47.61905	0.99573	0.99147	21.761	100.26	4.6577	39.186	5.6255
		3000	71.42857	0.99788	0.99576	10.049	100.18	3.1651	42.245	4.2703

Table S6 (continued)

		6-P-F-Asp (μM)	S/K_m	R	R squared	Chi Squared	Y max	Y max error	IC_{50}	IC_{50} error
3469:F02	Trial 1	7.38	0.175714	0.96064	0.92283	311.37	101.17	12.443	18.917	7.0456
		15.7	0.37381	0.96663	0.93438	302.7	104.19	11.914	27.611	7.0103
		31.3	0.745238	0.97296	0.94665	175.7	102.55	9.1977	33.493	7.7241
		62.5	1.488095	0.983	0.96628	116.5	101.41	7.5588	27.9	5.6546
		125	2.97619	0.99304	0.98614	47.822	101.29	4.7369	34.002	3.7642
		250	5.952381	0.99145	0.98297	63.323	101.29	5.4597	31.8	4.0159
		500	11.90476	0.99726	0.99452	18.006	100.04	2.9385	32.678	2.4071
		750	17.85714	0.9918	0.98367	50.207	100.61	4.9302	33.709	4.3734
		1000	23.80952	0.99278	0.98562	42.266	100.85	4.5365	33.935	4.1927
		2000	47.61905	0.99147	0.983	47.724	101.08	4.8123	36.144	4.7269
		3000	71.42857	0.99624	0.9925	13.561	100.51	2.5781	52.581	4.84E+00
	Trial 2	7.38	0.175714	0.99373	0.98749	51.609	99.229	7.1424	41.219	6.0875
		15.7	0.37381	0.98987	0.97984	108.63	99.584	7.3254	41.346	6.916
		31.3	0.745238	0.98527	0.97076	175.93	100.86	9.0588	51.675	8.7522
		62.5	1.488095	0.99716	0.99432	33.209	100.17	3.9601	51.259	3.9349
		125	2.97619	0.99836	0.99672	17.855	100.32	2.9379	49.245	3.0012
		250	5.952381	0.99748	0.99497	27.085	100.58	3.6292	47.765	3.6441
		500	11.90476	0.99921	0.99842	8.0996	100.37	1.9938	46.144	2.061
		750	17.85714	0.99615	0.99232	42.004	100.97	4.5313	45.449	4.3285
		1000	23.80952	0.99967	0.99934	3.3403	100.26	1.2776	48.946	1.3965
		2000	47.61905	0.99461	0.98925	53.368	101.53	5.027	59.209	6.2678
		3000	71.42857	0.98957	0.97926	105.09	103.08	6.9004	67.239	9.2828

Table S6 (continued)

		6-P-F-Asp (µM)	S/K_m	R	R squared	Chi Squared	Y max	Y max error	IC_{50}	IC_{50} error
1524:G12	Trial 1	7.38	0.175714	0.98431	0.96886	168.78	98.575	9.1297	1.0829	0.23617
		15.7	0.37381	0.96454	0.93033	323.85	98.162	12.582	1.3944	0.43261
		31.3	0.745238	0.98653	0.97324	106.12	98.633	7.2089	1.5429	0.28941
		62.5	1.488095	0.9904	0.98089	72.363	99.152	5.9806	1.4374	0.24208
		125	2.97619	0.99377	0.98759	47.12	99.24	4.8087	1.5511	0.19702
		250	5.952381	0.99102	0.98212	69.758	99.071	5.8605	1.4693	0.23128
		500	11.90476	0.99505	0.99013	40.16	99.027	4.4118	1.5918	0.17164
		750	17.85714	0.9945	0.98904	46.188	98.969	4.7402	1.511	0.17533
		1000	23.80952	0.99376	0.98756	51.172	99.186	5.0234	1.3635	0.18186
		2000	47.61905	0.98952	0.97916	81.502	99.021	6.3409	1.4365	0.24909
		3000	71.42857	0.98247	0.96525	121.87	98.726	7.7502	1.6468	0.36354
	Trial 2	7.38	0.175714	0.97113	0.9431	102.78	101.07	7.08	1.9142	0.31068
		15.7	0.37381	0.97175	0.9443	130.39	99.461	8.0548	1.4903	0.30306
		31.3	0.745238	0.97299	0.9467	120.57	99.318	7.7431	1.541	0.30269
		62.5	1.488095	0.98714	0.97445	52.672	99.453	5.1131	1.6437	0.21136
		125	2.97619	0.98387	0.96799	82.055	99.631	6.3881	1.3641	0.20395
		250	5.952381	0.98566	0.97152	78.725	99.583	6.2595	1.2686	0.18486
		500	11.90476	0.98763	0.97542	60.544	99.673	5.4854	1.4103	0.18012
		750	17.85714	0.98388	0.96803	78.587	99.569	6.2504	1.4181	0.20918
		1000	23.80952	0.98325	0.96678	78.866	99.576	6.265	1.4392	0.22436
		2000	47.61905	0.97929	0.95901	97.308	99.704	6.9682	1.3705	0.27477
		3000	71.42857	0.96394	0.92918	140.58	99.744	8.3817	1.6571	0.62258

Table S6 (continued)

		6-P-F-Asp (μM)	S/K_m	R	R squared	Chi Squared	Y max	Y max error	IC_{50}	IC_{50} error
1533:K12	Trial 1	7.38	0.175714	0.95111	0.90462	512.85	86.687	9.2461	26.238	1.425
		15.7	0.37381	0.91445	0.83621	642.96	89.941	13.208	24.067	4.9975
		31.3	0.745238	0.97326	0.94723	201.34	96.964	9.5425	18.531	4.1221
		62.5	1.488095	0.97985	0.96011	135.71	97.625	8.0509	18.319	3.8049
		125	2.97619	0.98929	0.9787	65.492	98.876	5.6609	18.071	2.9175
		250	5.952381	0.98768	0.9755	81.116	97.463	6.1102	19.644	2.854
		500	11.90476	0.99503	0.99009	35.293	98.287	4.0161	18.688	1.751
		750	17.85714	0.99433	0.98869	39.24	97.665	4.1094	20.08	1.7934
		1000	23.80952	0.9933	0.98665	49.485	98.539	4.8649	16.689	2.0704
		2000	47.61905	0.98188	0.96409	135.78	96.243	7.7494	18.576	3.2916
		3000	71.42857	0.9737	0.94809	197.56	92.243	7.8675	21.23	3.0373
	Trial 2	7.38	0.175714	0.95963	0.92089	267.51	98.502	16.287	24.947	9.8435
		15.7	0.37381	0.99188	0.98383	58.996	98.954	7.6093	24.666	4.1599
		31.3	0.745238	0.99505	0.99012	32.743	99.098	5.6506	26.495	3.2391
		62.5	1.488095	0.99592	0.99186	27.451	99.237	5.1834	25.898	2.9399
		125	2.97619	0.99734	0.99469	19.047	99.493	4.3347	23.987	2.3461
		250	5.952381	0.99745	0.9949	20.634	99.306	4.4891	23.759	2.3191
		500	11.90476	0.99775	0.9955	17.228	99.641	4.1351	21.772	2.0937
		750	17.85714	0.99884	0.99769	9.5835	99.751	3.0857	20.513	1.4541
		1000	23.80952	0.9988	0.9976	10.438	99.678	3.2146	21.057	1.5099
		2000	47.61905	0.99773	0.99546	20.329	99.557	4.487	20.772	2.0752
		3000	71.42857	0.99977	0.99955	2.1525	100.16	1.4579	20.712	0.6564

Table S7: Curve-fit parameters for $1/IC_{50}$ versus v_o/V_{max} plots featured in **Figure S12**.

Compound	Trial	R	R squared	Adjusted R squared	Standard error	F value	P value	Y intercept	Y intercept error	Y intercept t value
1524:E12	1	0.16767	0.028115	-0.11073	0.0055045	0.20249	0.66632	0.20031	0.0057155	35.047
	2	0.45183	0.20415	0.10467	0.020282	2.0521	0.18989	0.1869	0.016933	11.037
1524:G12	1	0.052448	0.0027508	-0.13971	0.037728	0.019309	0.8934	0.68306	0.035597	19.189
	2	0.64118	0.41111	0.32699	0.042755	4.8868	0.06274	0.60038	0.046711	12.853
1533:K12	1	0.79225	0.62765	0.58111	0.0042657	13.485	0.0062891	0.039226	0.0035168	11.154
	2	0.72983	0.53264	0.46588	0.0028766	7.9779	0.025607	0.036753	0.0022647	16.228
3470:A09	1	0.47187	0.22266	0.11161	0.0053601	2.0051	0.19969	0.040383	0.0053502	7.5479
	2	0.90447	0.81806	0.78774	0.0059312	26.978	0.0020266	0.05872	0.004662	12.595
3469:N17	1	0.9512	0.90479	0.89288	0.0013523	76.022	2.34E-05	0.019963	0.0010225	19.524
	2	0.52064	0.27107	0.19008	0.0030949	3.3469	0.10058	0.021875	0.0023034	9.4966
3469:F02	1	0.76197	0.5806	0.52817	0.0050941	11.075	0.010417	0.045698	0.0040578	11.262
	2	0.52236	0.27286	0.16899	0.0016593	2.6268	0.1491	0.02354	0.0014281	16.483

Table S7 (continued)

Compound	Trial	Y intercept p value	Y intercept lower 95%	Y intercept upper 95%	Slope	Slope error	Slope t value	Slope p value	Slope lower 95%	Slope upper 95%
1524:E12	1	4.00E-09	0.1868	0.21383	-0.0031492	0.0069984	-0.44999	0.66632	-0.019698	0.013399
	2	4.04E-06	0.14785	0.22594	0.031172	0.02176	1.4325	0.18989	-0.019007	0.081351
1524:G12	1	2.60E-07	0.59888	0.76723	-0.0063508	0.045703	-0.13896	0.8934	-0.11442	0.10172
	2	4.01E-06	0.48992	0.71083	0.1237	0.055957	2.2106	0.06274	-0.0086178	0.25602
1533:K12	1	3.74E-06	0.031116	0.047335	0.016104	0.0043854	3.6722	0.0062891	0.0059915	0.026217
	2	8.21E-07	0.031398	0.042108	0.0083571	0.0029588	2.8245	0.025607	0.0013607	0.015354
3470:A09	1	0.0001319	0.027731	0.053034	0.0094245	0.0066557	1.416	0.19969	-0.0063137	0.025163
	2	1.53E-05	0.047312	0.070127	0.033896	0.006526	5.194	0.0020266	0.017928	0.049865
3469:N17	1	4.92E-08	0.017605	0.022321	0.011533	0.0013228	8.719	2.34E-05	0.0084831	0.014584
	2	5.49E-06	0.016664	0.027085	0.00536	0.0029298	1.8294	0.10058	-0.0012678	0.011988
3469:F02	1	3.47E-06	0.036341	0.055056	-0.017219	0.0051742	-3.3279	0.010417	-0.029151	-0.0052874
	2	7.38E-07	0.020163	0.026917	-0.0029679	0.0018312	-1.6207	0.1491	-0.0072981	0.0013622

Table S8: List of commercially purchased compounds that are shaded red if they inhibit FraB-G6PD by more than 30% using either 0.2 mM substrate and 0.25 mM inhibitor (A), 0.2 mM substrate and 0.025 mM inhibitor (B), or 1 mM substrate and 0.025 mM inhibitor (C) or inhibit G6PD by more than 20%, using 0.05 mM G-6-P and 0.25 mM inhibitor (D), as depicted in **Figure 4**.

PubChem CID	A	B	C	D
9112296				
4016579				
5027930				
8107428				
2554464				
8026948				
2369676				
16286851				
4833218				
737312				
46863682				
1630760				
16295900				
3812421				
2419775				
2480372				
2398038				
9115650				
598190				
2418771				
19989				
20910782				
43988681				
4114336				
805159				
4552189				
3900860				
697338				
1652998				
482604				
2386913				
50760510				
20931114				
44116999				
16421618				
2412550				
243573				
2513350				
2392334				
42570666				
17598362				
20995141				
5153171				
78304				
3157968				
4900670				
4596023				
2952567				
2988604				
1167097				
746333				
4373469				
3722427				
3311049				
5129475				
583717				
248003				
739239				
135477731				
2987165				

PubChem CID	A	B	C	D
1160380				
3153602				
712812				
795351				
856396				
2831814				
672225				
571273				
672972				
5173955				
2835294				
687336				
83700				
9905811				
5908				
673890				
5455				
5353432				
757102				
5368832				
708768				
305201				
673246				
5015146				
725388				
1272327				
141175				
2431970				
101777				
2745697				
2366666				
2732190				
2743226				
2743927				
2743973				
2813609				
2813608				
2802539				
2807908				
747450				
2807955				
2743239				
2805686				
2801788				
2810370				
2810371				
1240105				
17590187				
3156098				
5488995				
41905307				
17597502				
16764760				
16424047				
16418211				
441373				
759209				
3194				
5349289				
806000				
6496899				

Table S9: Theoretical and experimental masses of FraB, FraB-inhibitor complexes, and FraB-inhibitor-substrate complexes measured by nMS.

a. Determination of the mass of FraB bound to different triazolothiadiazole inhibitors.

Species	Theoretical mass (Da)	Vendor: Ambinter		Vendor: ChemDiv	
		Experimental mass (Da)	Difference (Da)	Experimental mass (Da)	Difference (Da)
E_2	76,278	76,278	0	76,278	0
E_2I (A09)	76,637	76,637	0	76,636	-1
E_2I_2 (A09)	76,996	76,997	+1	76,996	0
E_2I (F02)	76,615	76,614	-1	76,615	0
E_2I_2 (F02)	76,953	76,952	-1	76,953	0
E_2I (N17)	76,575	76,576	+1	76,576	+1
E_2I_2 (N17)	76,873	76,873	0	76,873	0

b. Determination of the mass of FraB bound to 6-P-F-Asp and 3469:N17.

Species	Theoretical mass (Da)	Vendor: Ambinter		Vendor: ChemDiv	
		Experimental mass (Da)	Difference (Da)	Experimental mass (Da)	Difference (Da)
E_2	76,278	76,278	0	76,278	0
E_2I	76,575	76,576	+1	76,576	+1
E_2I_2	76,873	76,874	+1	76,873	0
E_2P	76,538	76,538	0	76,538	0
E_2S	76,653	76,655	+2	76,654	+1
E_2P_2	76,798	76,798	0	76,797	-1
E_2IP	76,835	76,836	+1	76,835	0
E_2SP	76,913	76,915	+2	76,914	+1
E_2IS	76,950	76,953	+3	76,952	+2
E_2IP_2	77,095	77,096	+1	77,096	+1
E_2I_2P	77,132	77,131	-1	77,130	-2
E_2ISP	77,210	77,212	+2	77,212	+2
$E_2I_2P_2$	77,392	77,389	+4	77,392	0

Abbreviations used: E_2 - FraB dimer; I - inhibitor (3469:N17); S - substrate (6-P-F-Asp); P - product (Glc-6-P), one of the two products generated by FraB.

SUPPLEMENTARY FIGURES

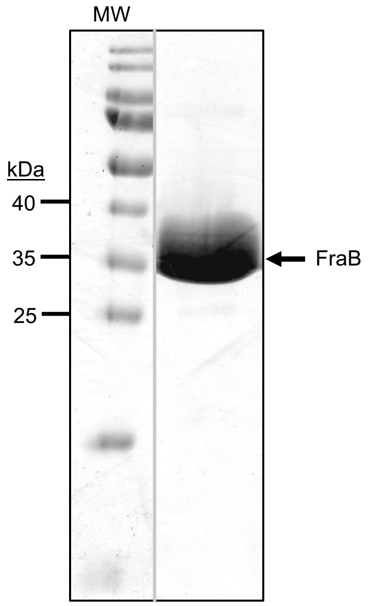

Figure S1 SDS-PAGE analysis of purified His_6-FraB wild-type using a 15% (w/v) polyacrylamide gel. The expected size of His_6-FraB is ~38 kDa. PageRuler Prestained Protein Ladder (Thermo Scientific, product #26616) was used as the molecular weight (MW) reference markers.

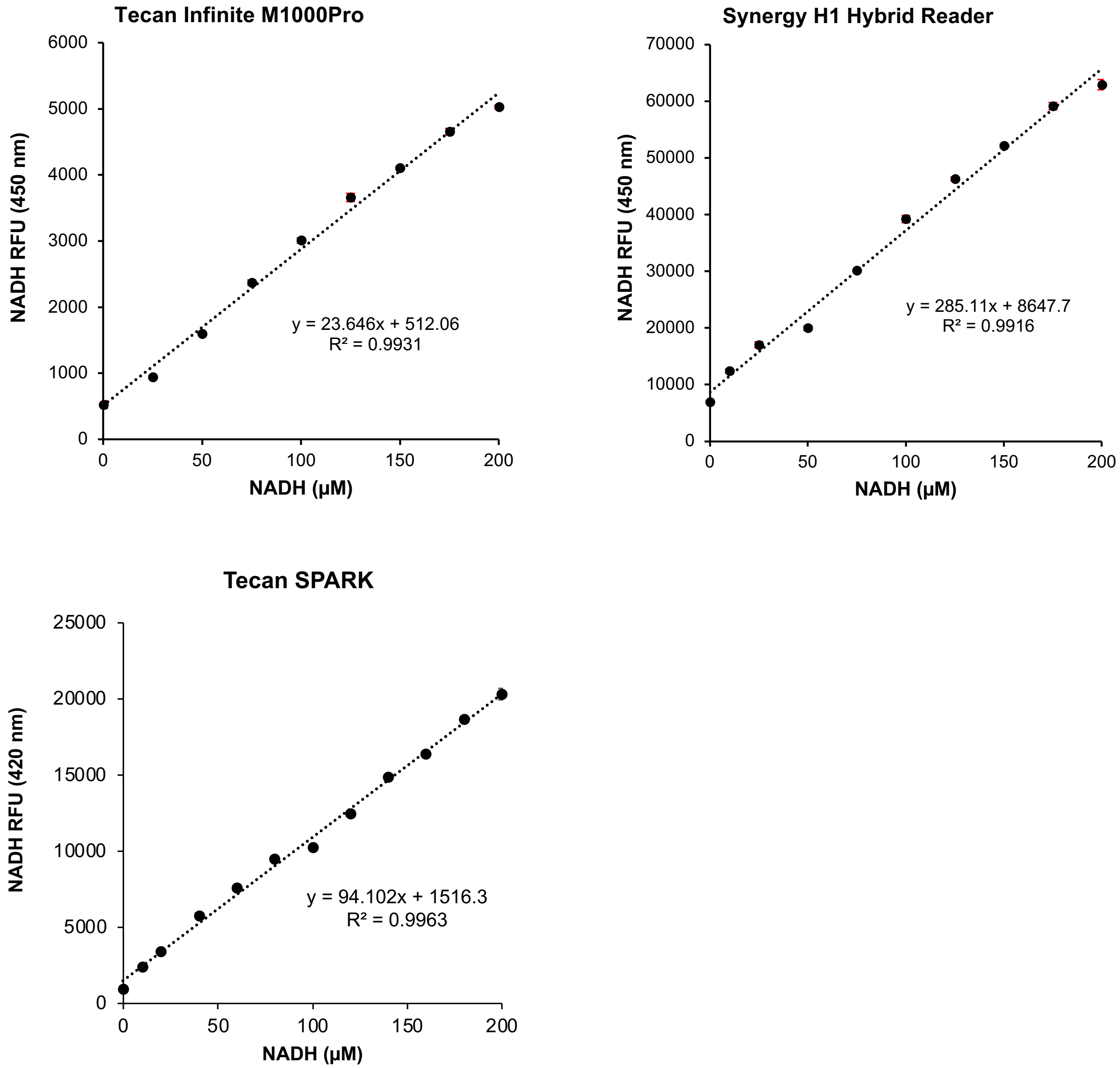

Figure S2 Standard curves for NADH relative florescence units (RFUs) versus concentration (µM) using either the Tecan Infinite M1000Pro, Tecan SPARK, or Synergy H1 Hybrid Reader instrument. Standard deviation bars are colored red but are not visible here given the minimal variation in the measured fluorescence values.

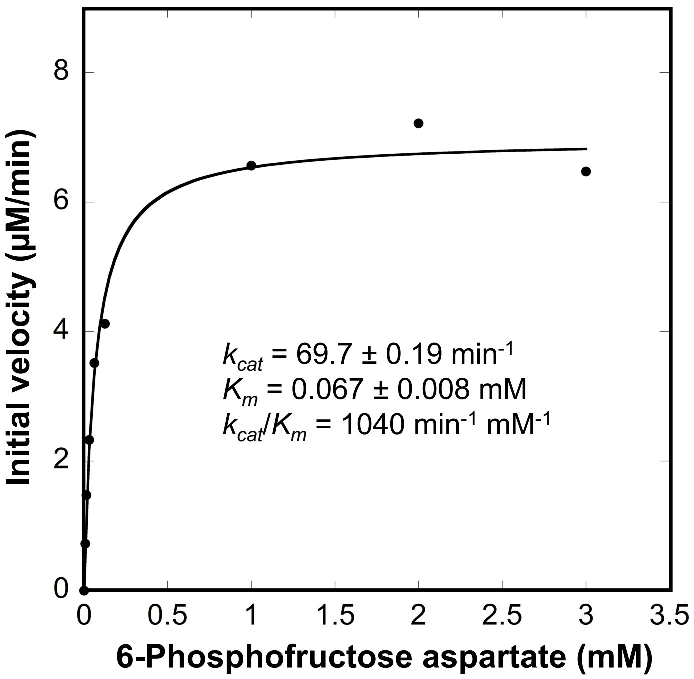

Figure S3 Representative Michaelis-Menten plot showing the change in initial velocity of FraB-G6PD as a function of 6-P-F-Asp concentration with curve-fit errors. Kaleidagraph was used to fit the data to formula $v_o = (V_{max} \times [S])/(K_m + [S])$. Curve-fit errors were 12% and 0.3% for the K_m and k_{cat} values, respectively, and goodness of fit was 0.99.

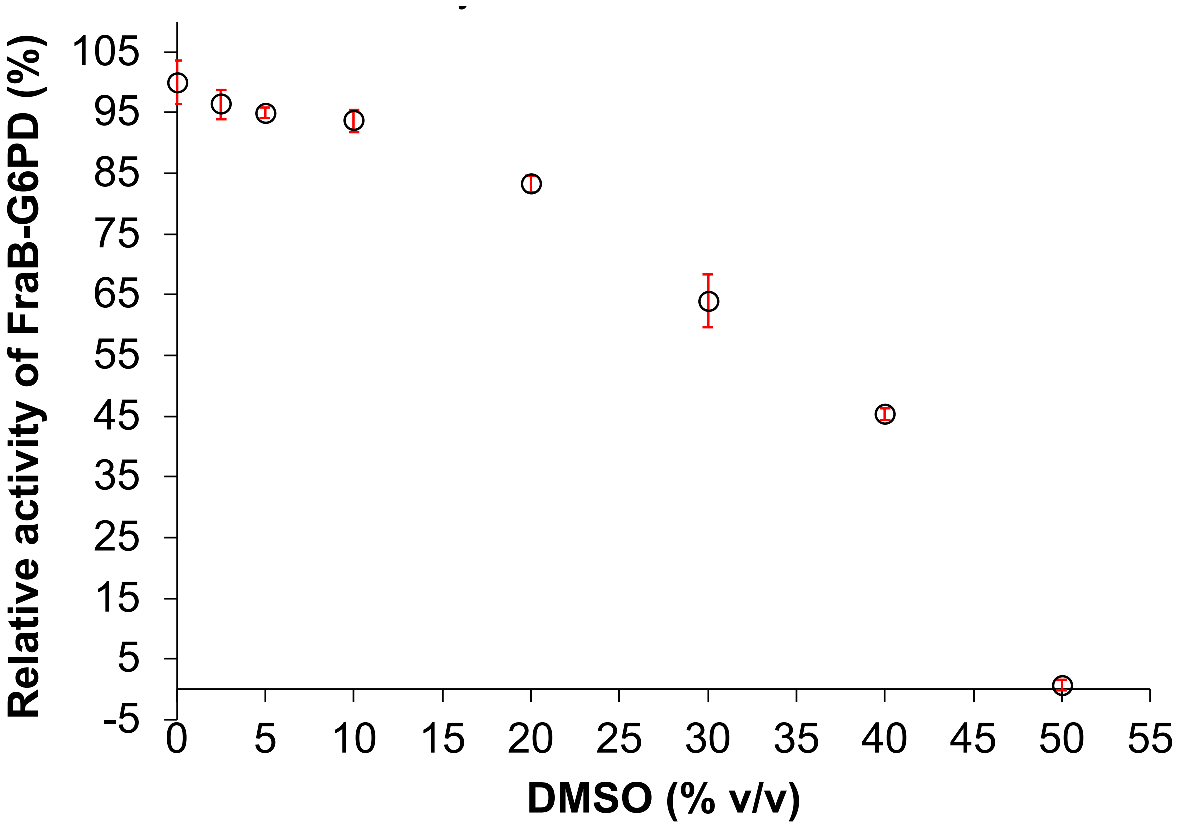

Figure S4 Relative activity of FraB-G6PD in the presence of 0-50% (v/v) DMSO. Error bars for 40% (v/v) DMSO reflects standard error whereas all others reflect standard deviation.

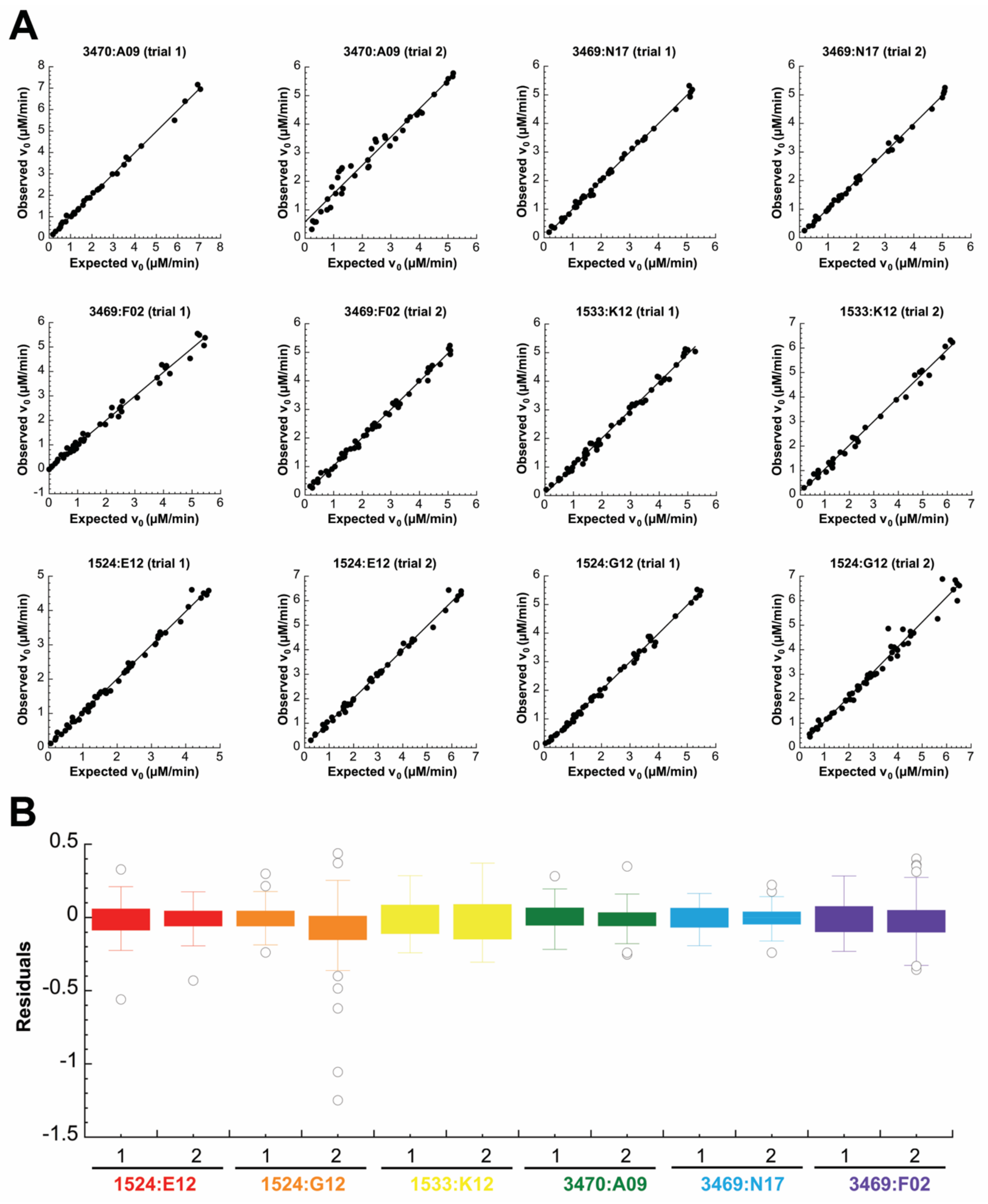

Figure S5 Correlation plots (*A*) and residuals (*B*) between expected and observed initial velocities of FraB from two trials. Curve- fit data for these plots are listed in **Table S6**.

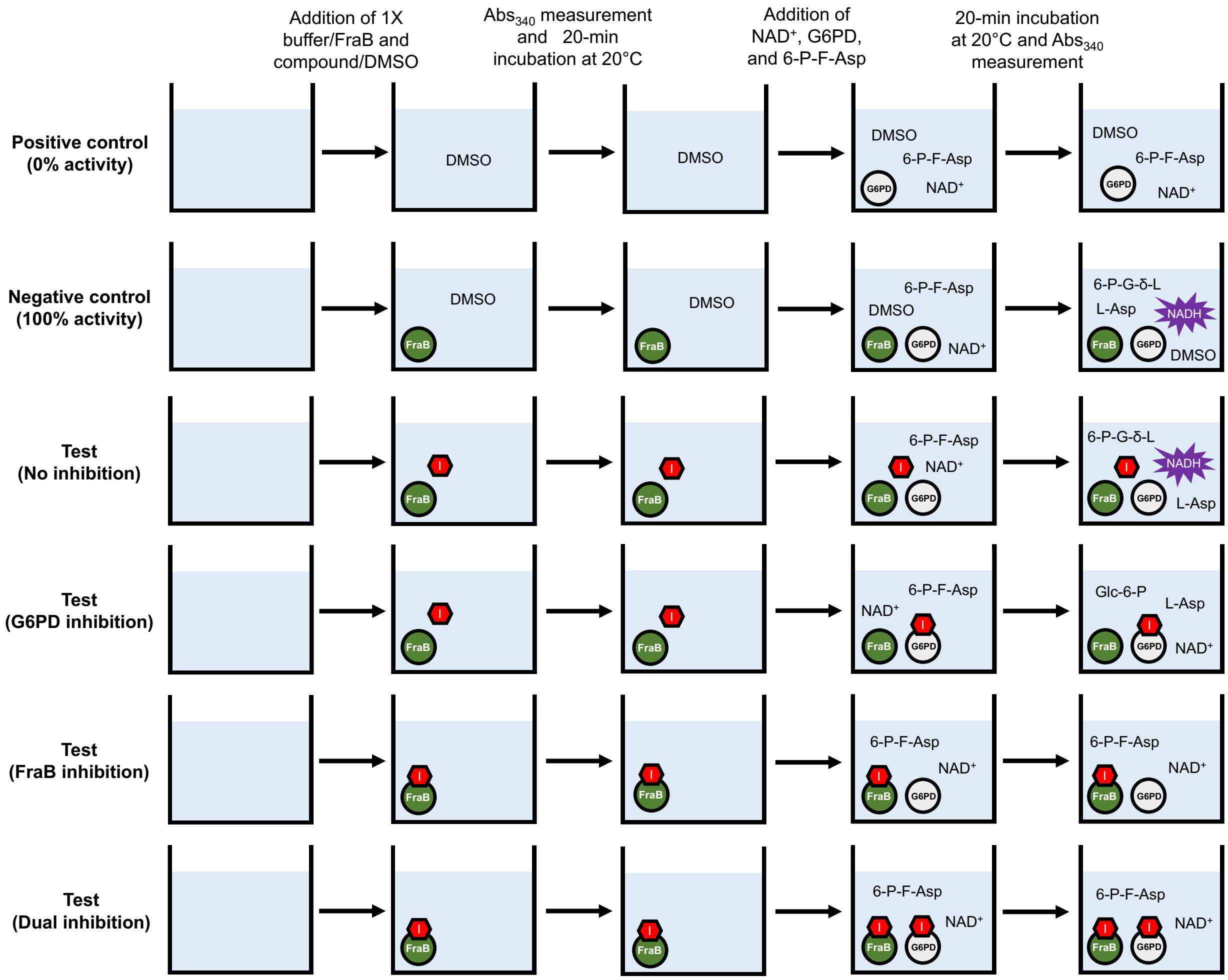

Figure S6 Workflow for HTS of the small-molecule libraries with a depiction of expected outcomes. HTS was performed at the ICCB-Longwood Screening Facility (Harvard). In the presence of G6PD (gray circle) and 6-P-F-Asp, no activity is expected. In the presence of FraB (green circle), G6PD, and 6-P-F-Asp, full activity is expected where Glc-6-P and NAD^+ and converted to 6-phosphoglucono-δ-lactone (6-P-G-δ-L) and NADH. In the presence of FraB, G6PD, 6-P-F-Asp, and an inhibitor (red hexagon) dissolved in 100% DMSO, variable activity is expected. Inhibition of G6PD, FraB, or both enzymes (dual inhibition) is shown to be complete in the above examples for simplicity.

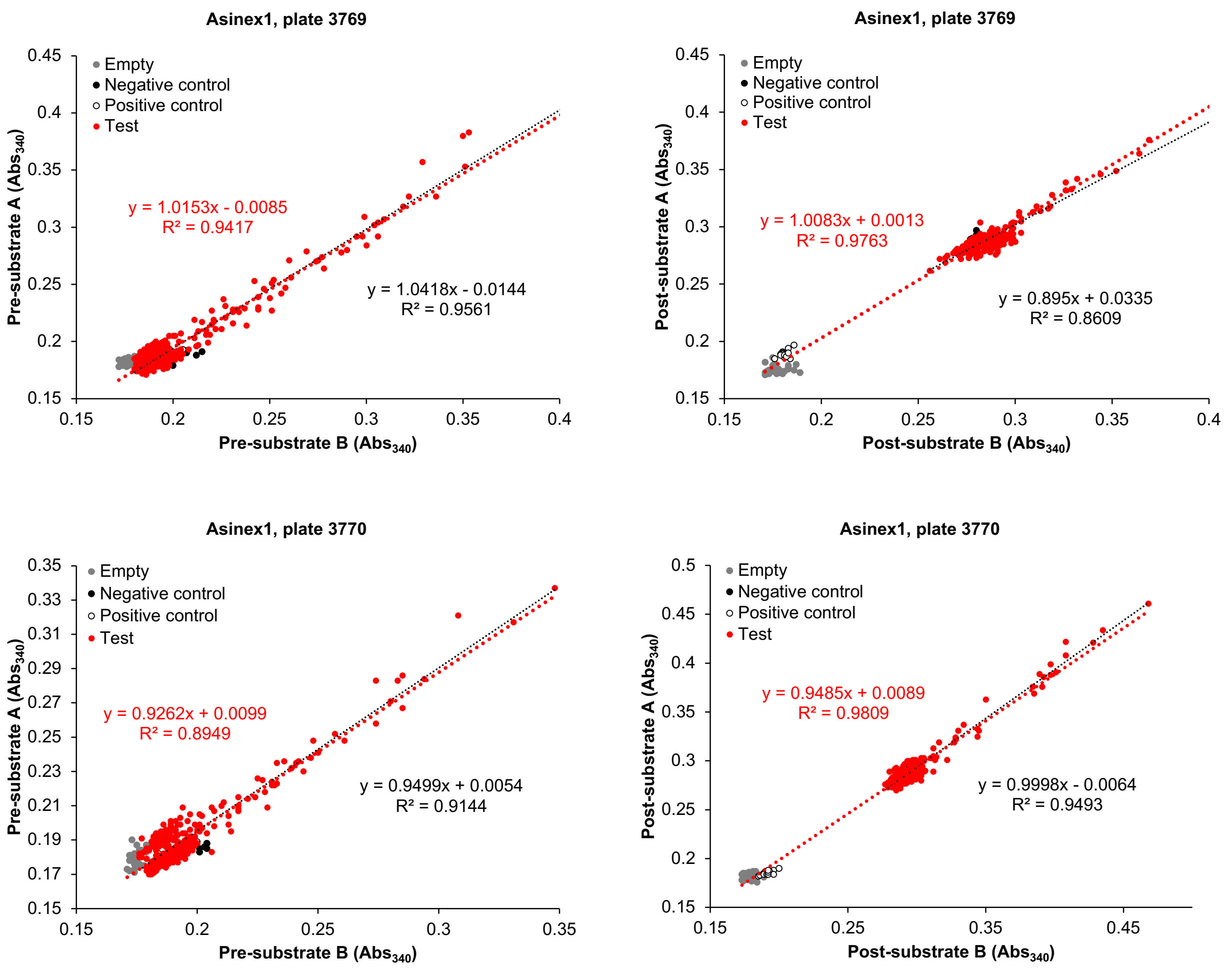

Figure S7 (continued on the next page)

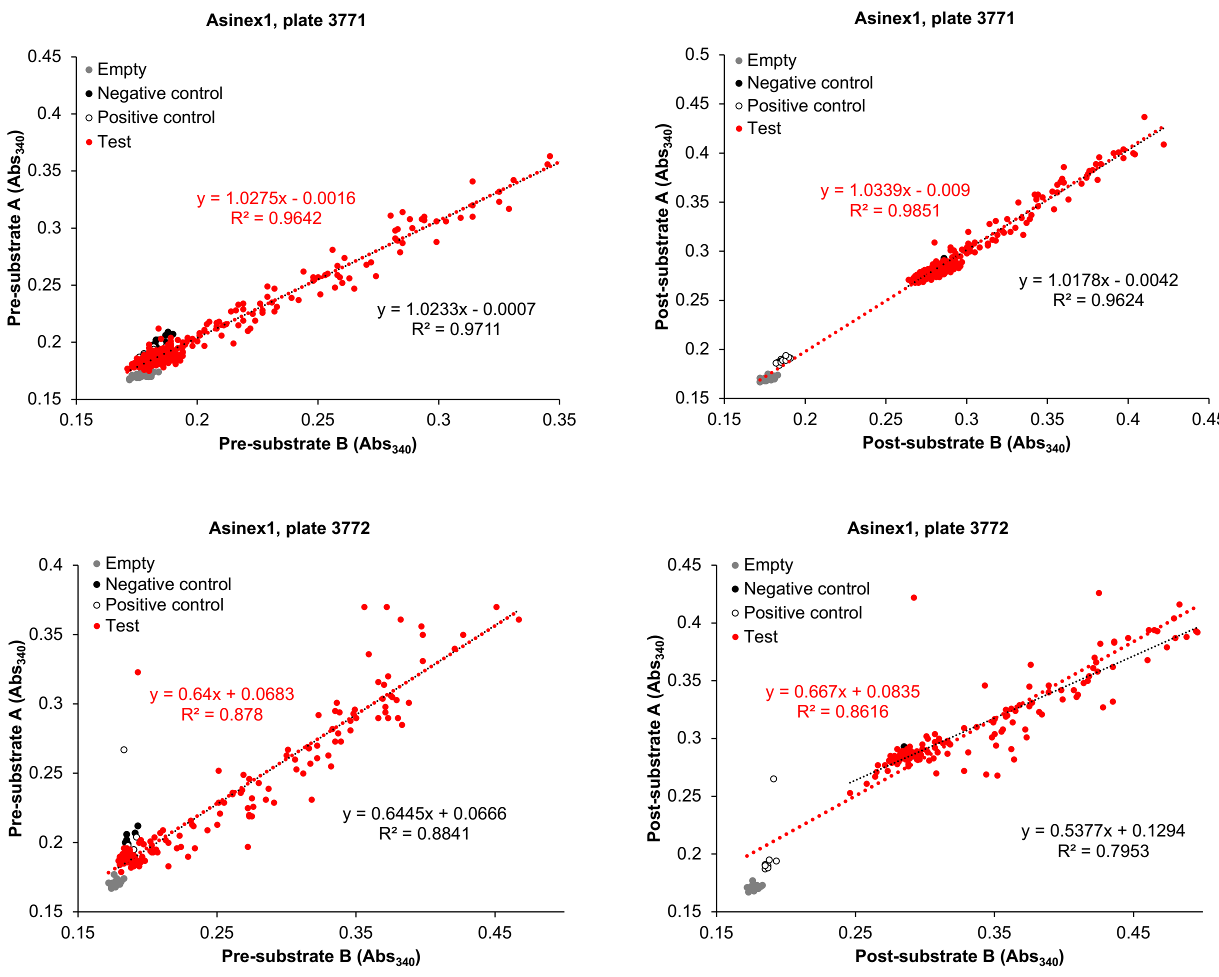

Figure S7 Correlation plots showing the absorbances (from two independent trials) of wells containing either nothing (empty), G6PD (positive control), FraB and G6PD (negative control), or FraB and G6PD plus compounds from plates 3769-3772 of the Asinex1 library (test) before (left graphs) and after (right graphs) substrate addition. The black-colored dotted linear regression trendline only includes test samples whereas the red-colored dotted trendline includes all samples.

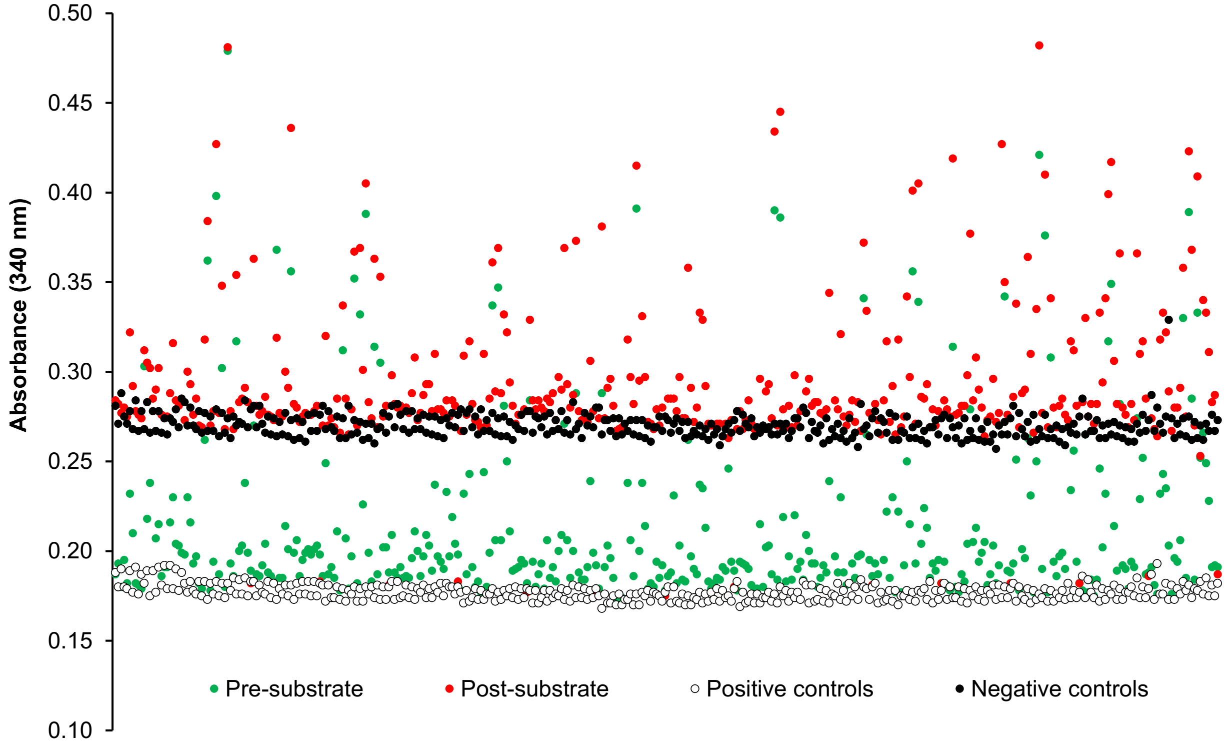

Figure S8 Representative HTS data for a subset of compounds screened at the ICCB-Longwood Screening Facility showing the absorbance of test wells either before (green filled circles) or after (red filled circles) addition of substrate. Control reactions were performed in either the absence (positive) or presence (negative) of FraB and demarcate the lower and upper limits for the assay. Test wells that show absorbance greater than the absorbance of the controls, both before and after substrate addition, arise from inherent absorbances of compounds that are independent of FraB-G6PD activity.

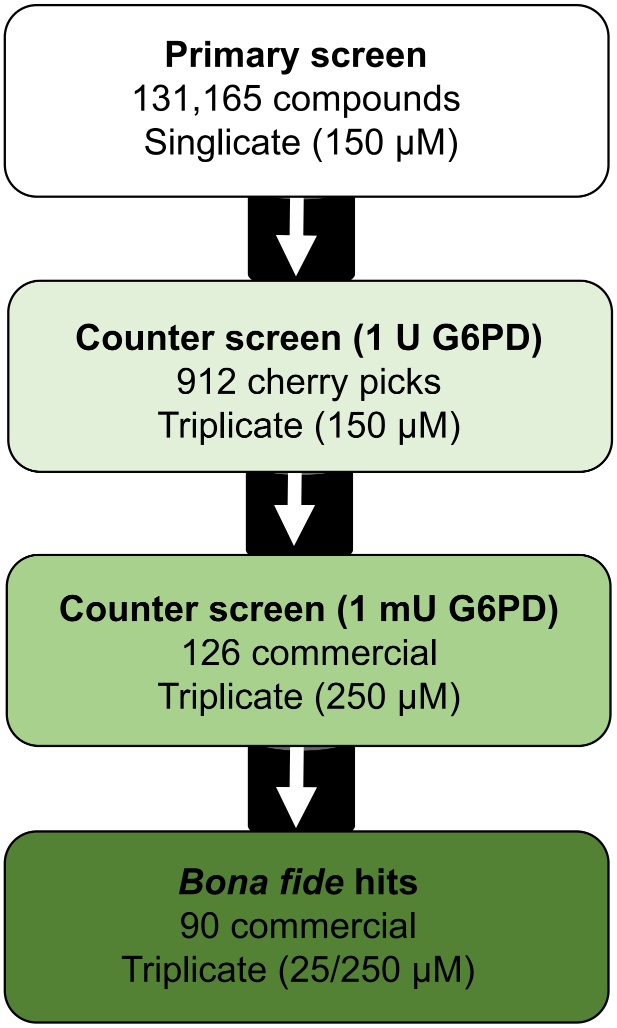

Figure S9 Summary of outcomes at different stages of our HTS campaign.

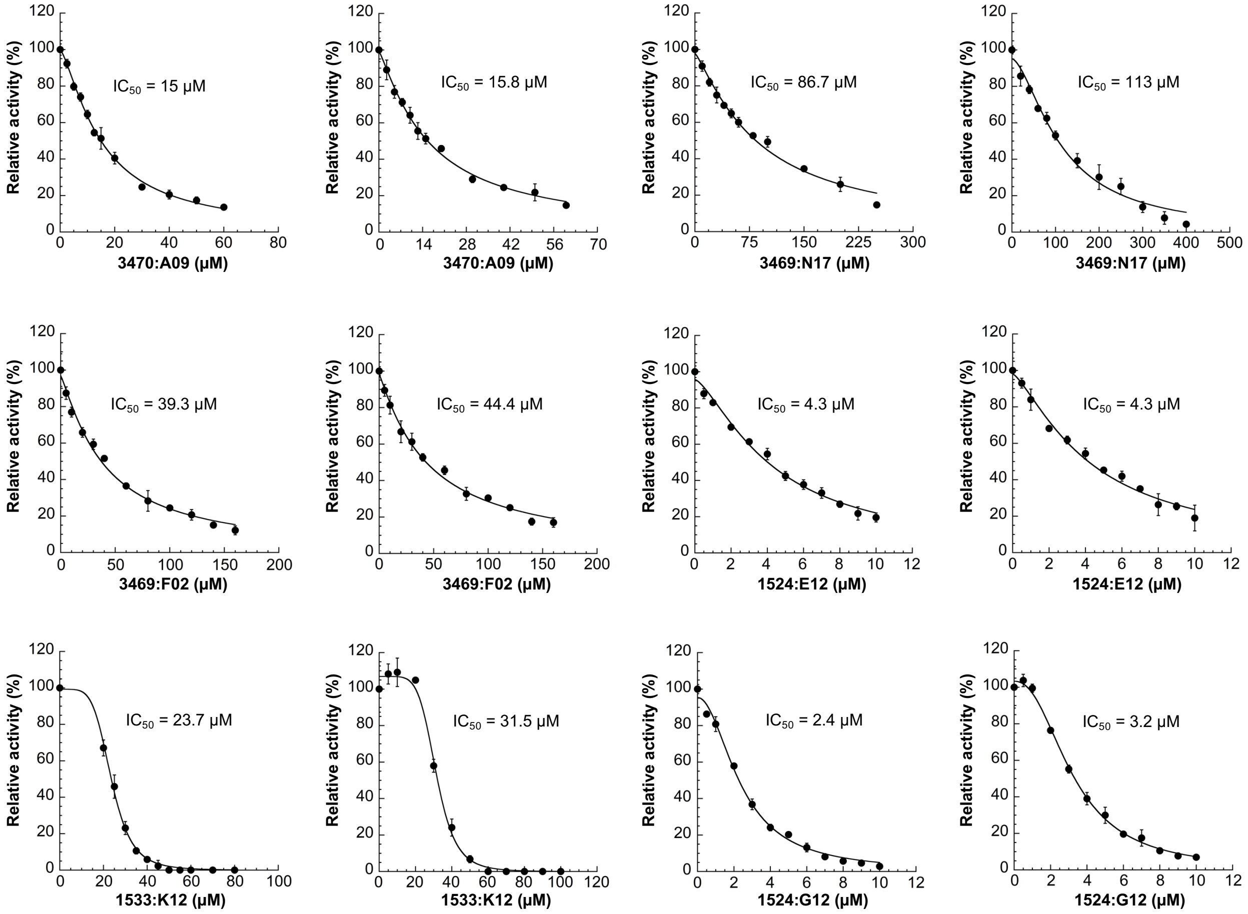

Figure S10 Determination of the IC_{50} values for six different inhibitors. Data from two independent trials for each inhibitor are shown; each plot depicts the average and standard deviation from triplicate or quadruplicate measurements at the indicated inhibitor concentration. Curve-fit data are listed in **Table S1**.

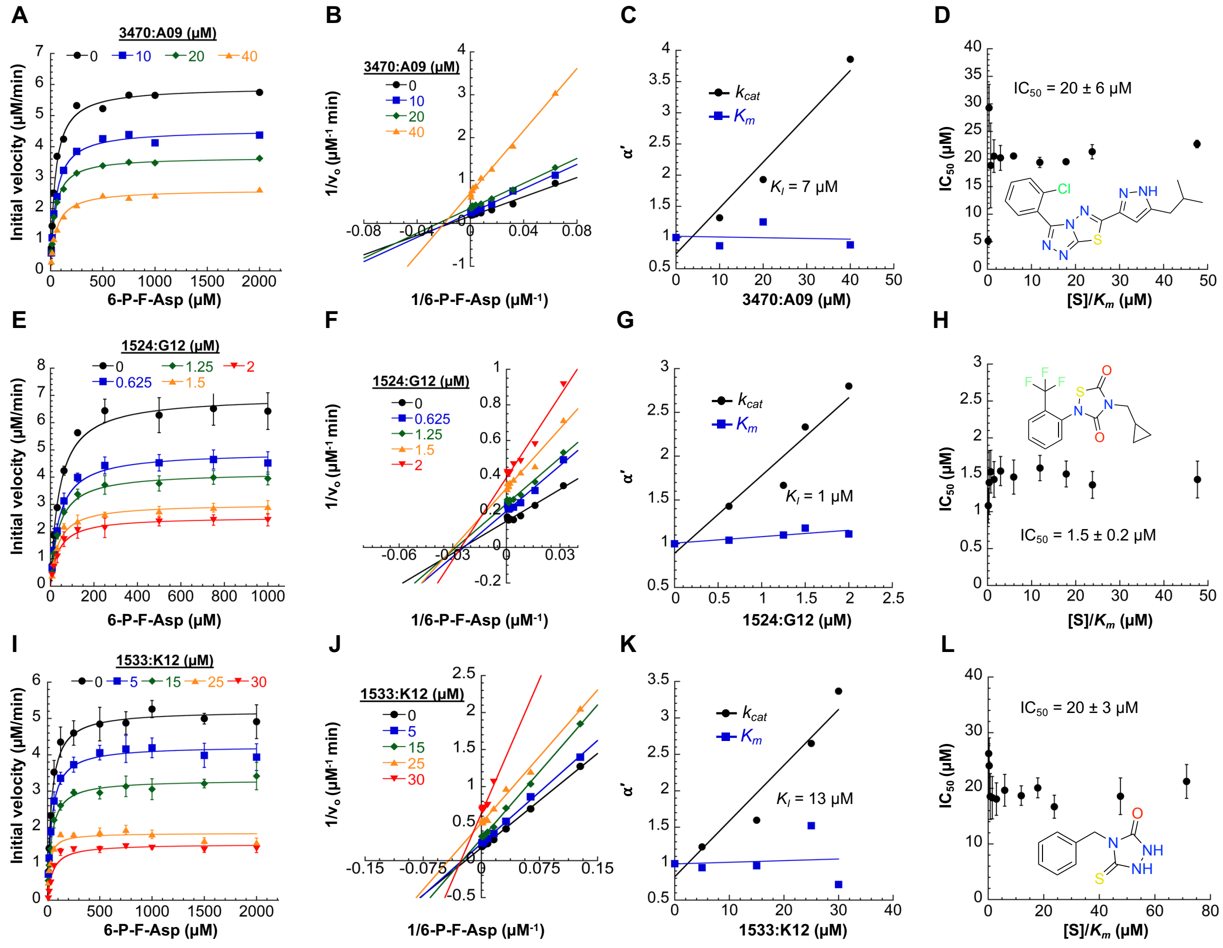

Figure S11 (continued on the next page)

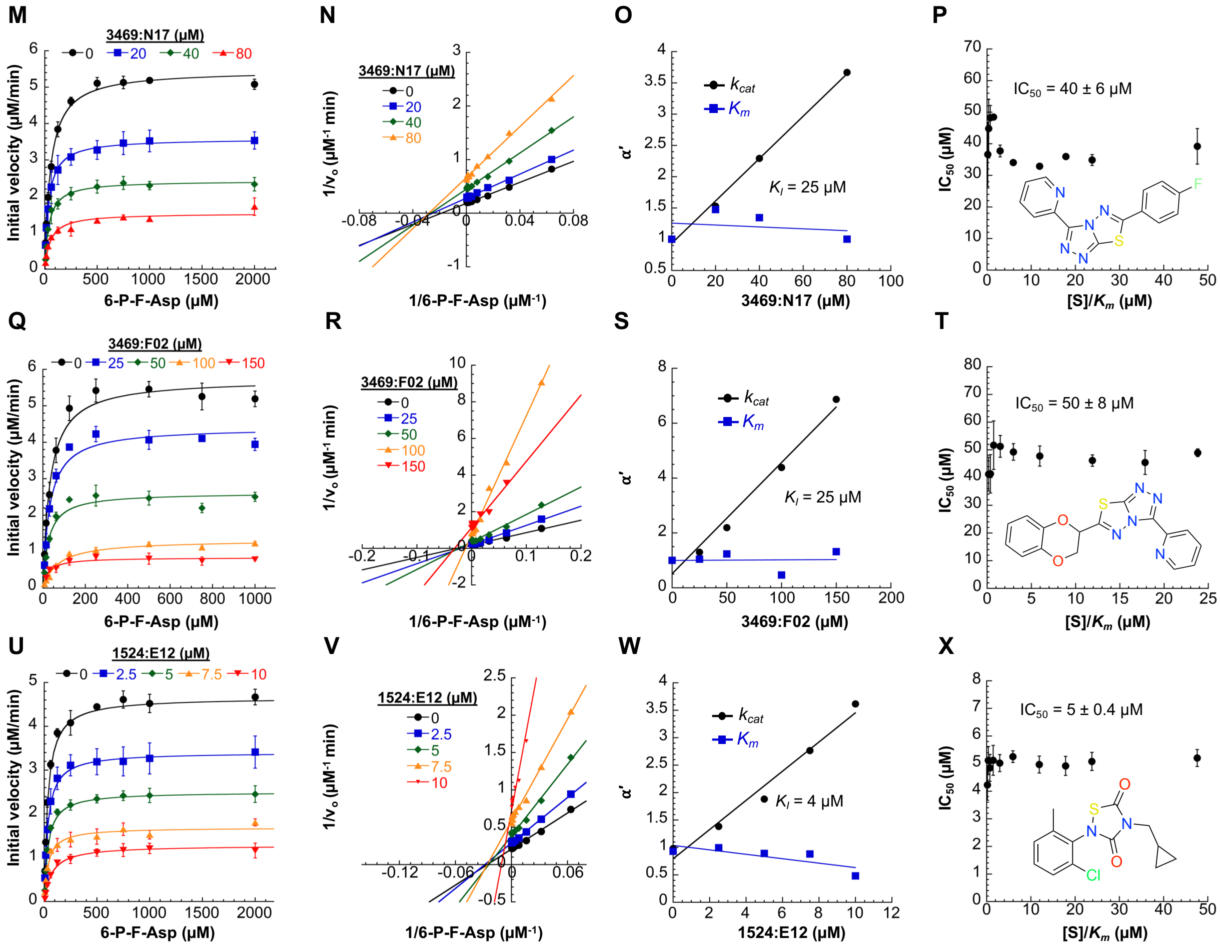

Figure S11 (continued on the next page)

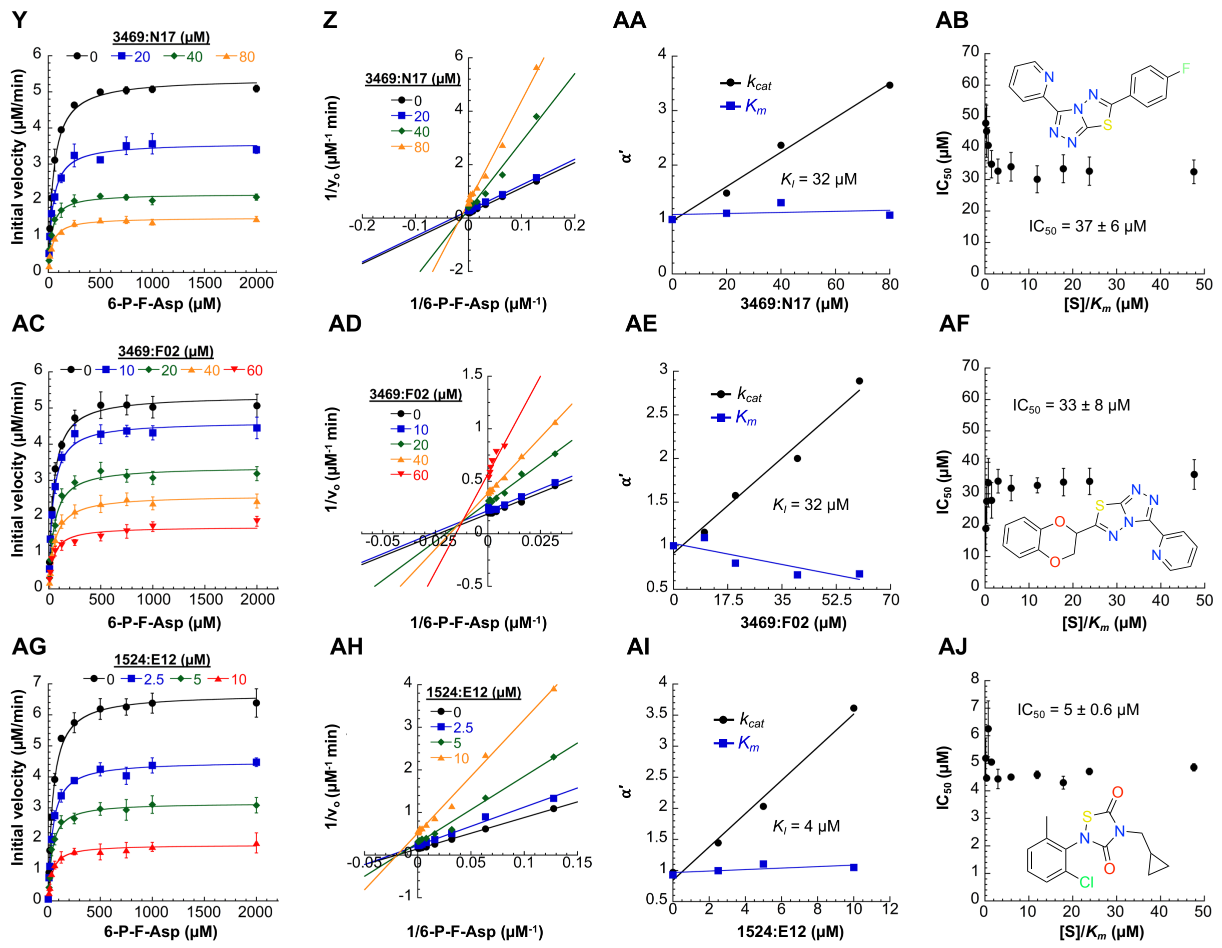

Figure S11 Characterization of the inhibition of *Salmonella* FraB by 3470:A09 (*A-D*), 1524:G12 (*F-H*), 1533:K12 (*I-L*), 3469:N17 (*M-P* and *Y-AB*), 3469:F02 (*Q-T* and *AC-AF*), and 1524:E12 (*U-X* and *AG-AJ*). Michaelis-Menten (*A*, *E*, *I, M, Q, U, Y, AC, and AG*), Lineweaver-Burk (*B, F, J, N, R, V, Z, AD, and AH*), α' vs [I] (*C, G, K, O, S, W, AA, AE, and AI*), and IC_{50} vs [S]/K_m (*D, H, L, P, T, X, AB, AF, and AJ*) plots are shown here. Data for the second trial for 3470:A09, 1524:G12, and 1533:K12 are shown in **Figure 5**. Data from two independent trials for each inhibitor are shown; each Michaelis-Menten plot depicts the average and standard deviation from a triplicate or quadruplicate measurement at the indicated substrate concentration. Averages and standard errors for

the K_I values are listed in **Table 1**. In panels depicting α' versus [I], $\alpha' = 1 + [I]/K_I'$. In the IC_{50} vs [S]/K_m plots, the mean and standard deviation values determined by taking all the IC_{50} values are listed. Note that the IC_{50} vs [S]/K_m plot data were derived by calculating the IC_{50} values from the Michaelis-Menten plots; there is good agreement between these values and those determined from the typical IC_{50} calculation (**Figure S10).** Curve-fit data for the different plots are listed in **Tables S2-S5**.

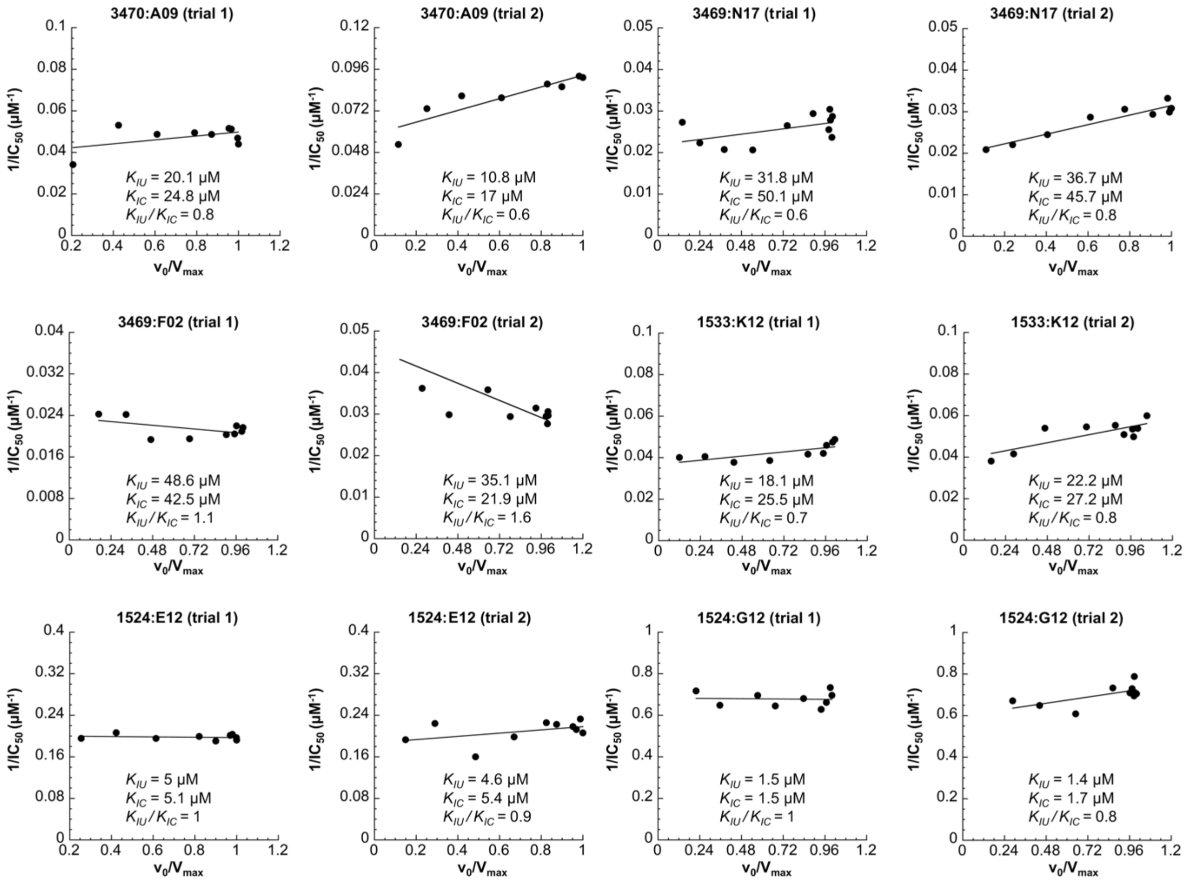

Figure S12 Determination of the K_{IU}, K_{IC}, and K_{IU}/K_{IC} values for six different inhibitors. Data from two independent trials for each inhibitor are shown; each plot depicts the average and standard deviation from triplicate or quadruplicate measurements at the indicated inhibitor concentration. Curve-fit data are listed in **Table S7**. The nomenclature used here for the inhibition constants follows those used by Cortés et al. (2001) *Biochem. J.* 357: 263-268. K_{IU} and K_{IC} are the uncompetitive and competitive inhibition constants, with these two being equal in the case of non-competitive inhibition (please see text for more details).

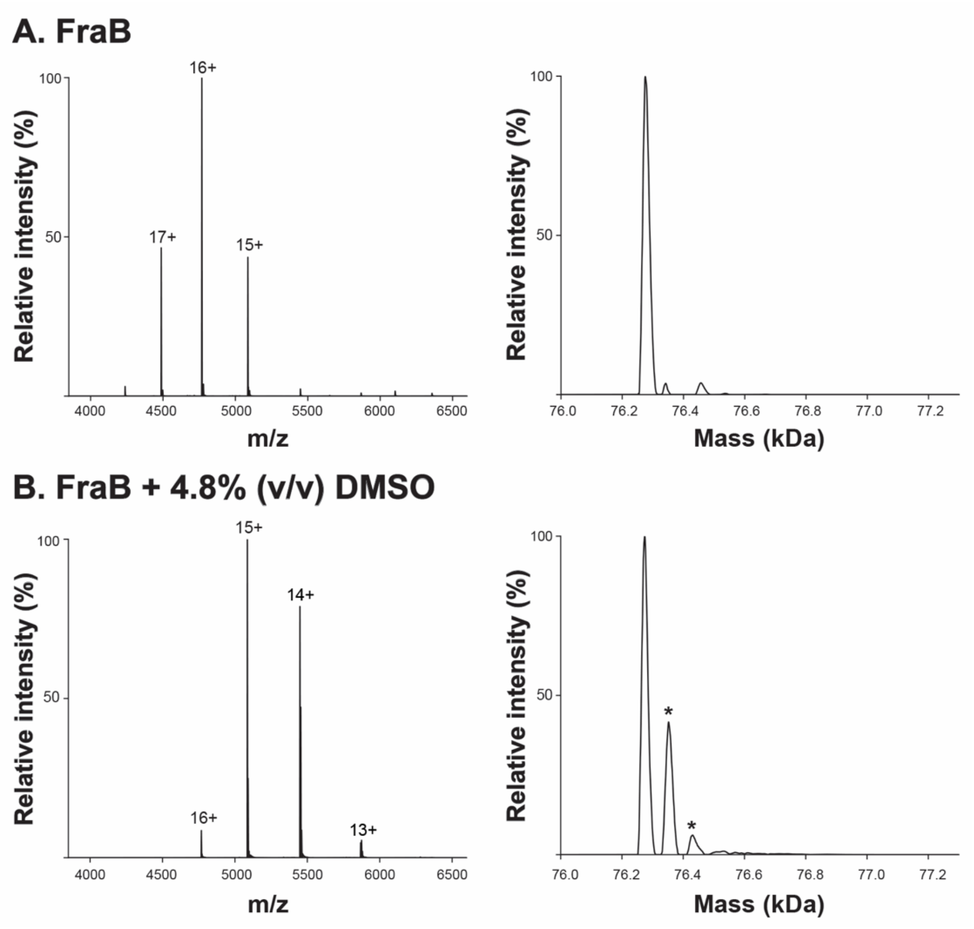

Figure S13 Primary (left) and deconvolved (right) native MS spectra for FraB in the absence (*A*) or presence of 4.8% (v/v) DMSO (*B*). The concentration of DMSO used is the same as those used in all native MS studies of FraB-inhibitor complexes (prepared with stocks prepared from ChemDiv; **Figures 6, 7**, **S14**, and **S17**). Asterisks (*) denote adducts from DMSO. The top right panel reveals a very small amount of other adducts, presumably nickel. Addition of small amounts of DMSO (as in panel *B*) results in lowering of charge, as is evident from the altered charge-state distribution (also, see **Figure S14**).

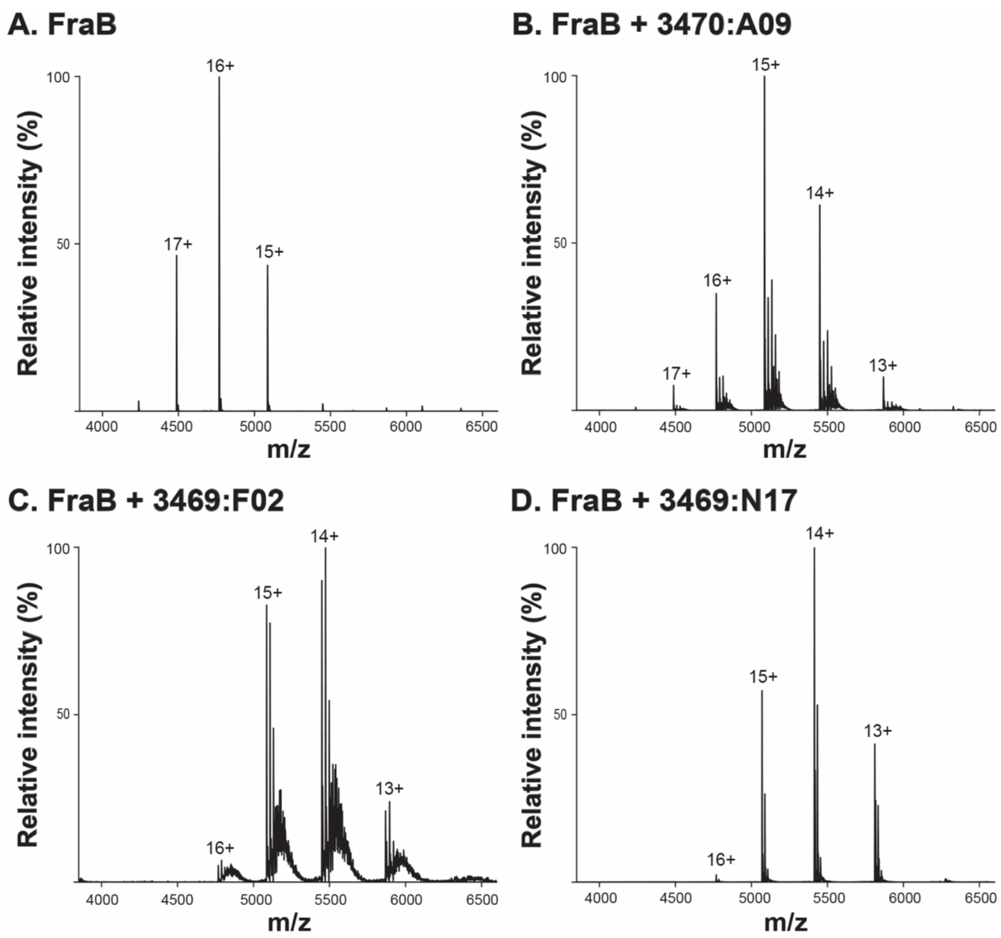

Figure S14 Primary native MS spectra corresponding to the deconvolved spectra shown in **Figure 6** for FraB either alone (*A*) or bound to inhibitor 3470:A09 (*B*), 3469:F02 (*C*), and 3469:N17 (*D*). Data were collected under IST 50 V. Note: These compounds were obtained from ChemDiv (also, see **Figure S15-S17** for MS data obtained with compounds purchased from Ambinter).

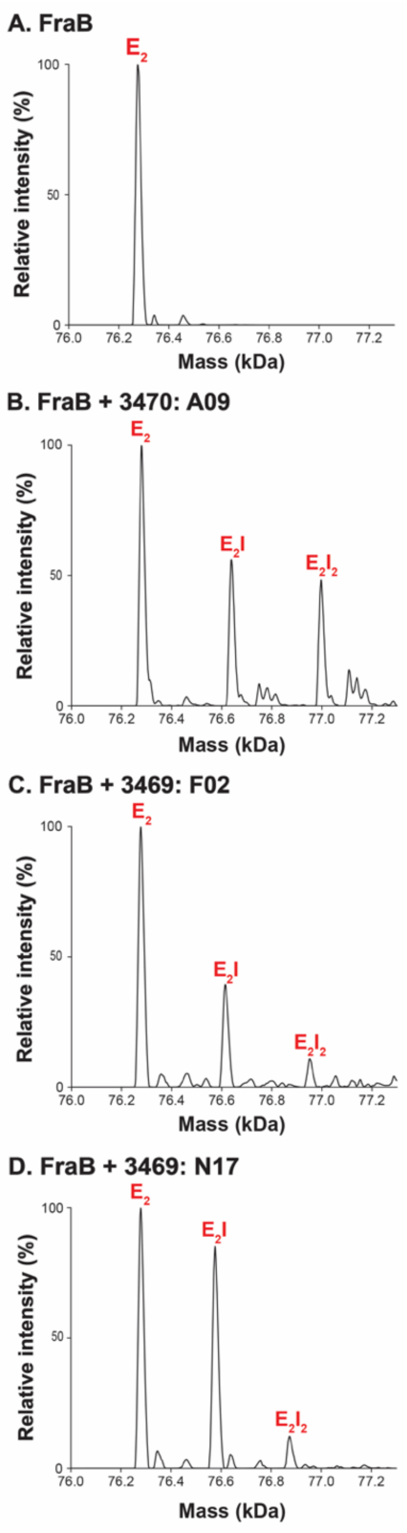

Figure S15 Deconvolved native mass spectra for FraB without (*A*) and with inhibitor 3470:A09 (*B*), 3469:F02 (*C*), and 3469:N17 (*D*) bound. E_2, FraB dimer alone; E_2I or E_2I_2,

FraB dimer bound to one or two inhibitor copies, respectively. FraB was diluted into 200 mM ammonium acetate and then with mixed with inhibitors (source, Ambinter) to yield final concentrations of 3 μM FraB, either 50 μM (A09) or 100 μM (FO2, N17) inhibitor, and 1% (v/v) DMSO. Inhibitor concentrations were chosen to be ~3-5-X their respective inhibition constant (Table 1). Data were collected under IST 50 V. Note: These compounds were obtained from Ambinter (see **Figure 6** for similar data obtained using compounds from ChemDiv).

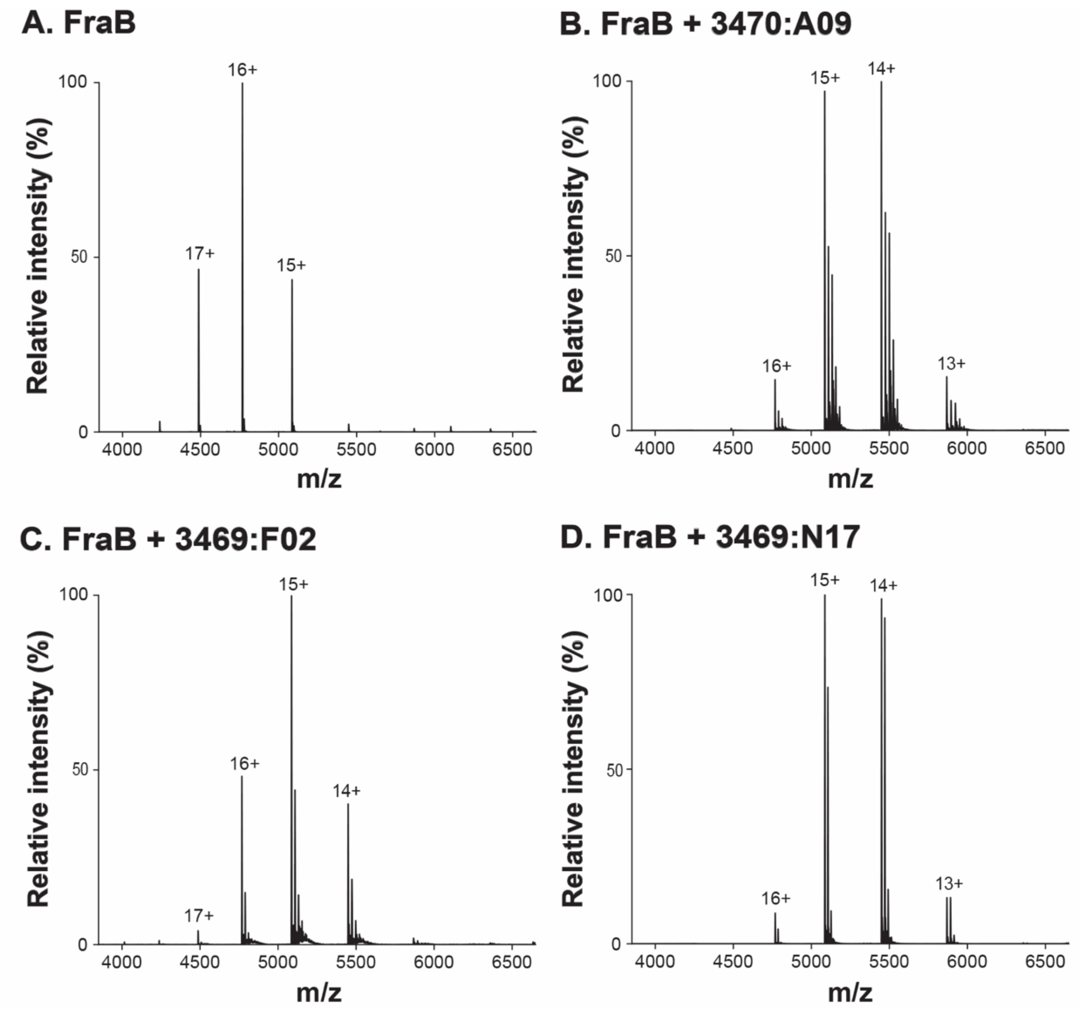

Figure S16 Primary native MS spectra corresponding to the deconvolved spectra shown in **Figure S15** for FraB either alone (*A*) or bound to inhibitor 3470:A09 (*B*), 3469:F02 (*C*), and 3469:N17 (*D*). Data were collected under IST 50 V. <u>Note</u>: These compounds were obtained from Ambinter (see **Figure S14** for similar data obtained using compounds from ChemDiv).

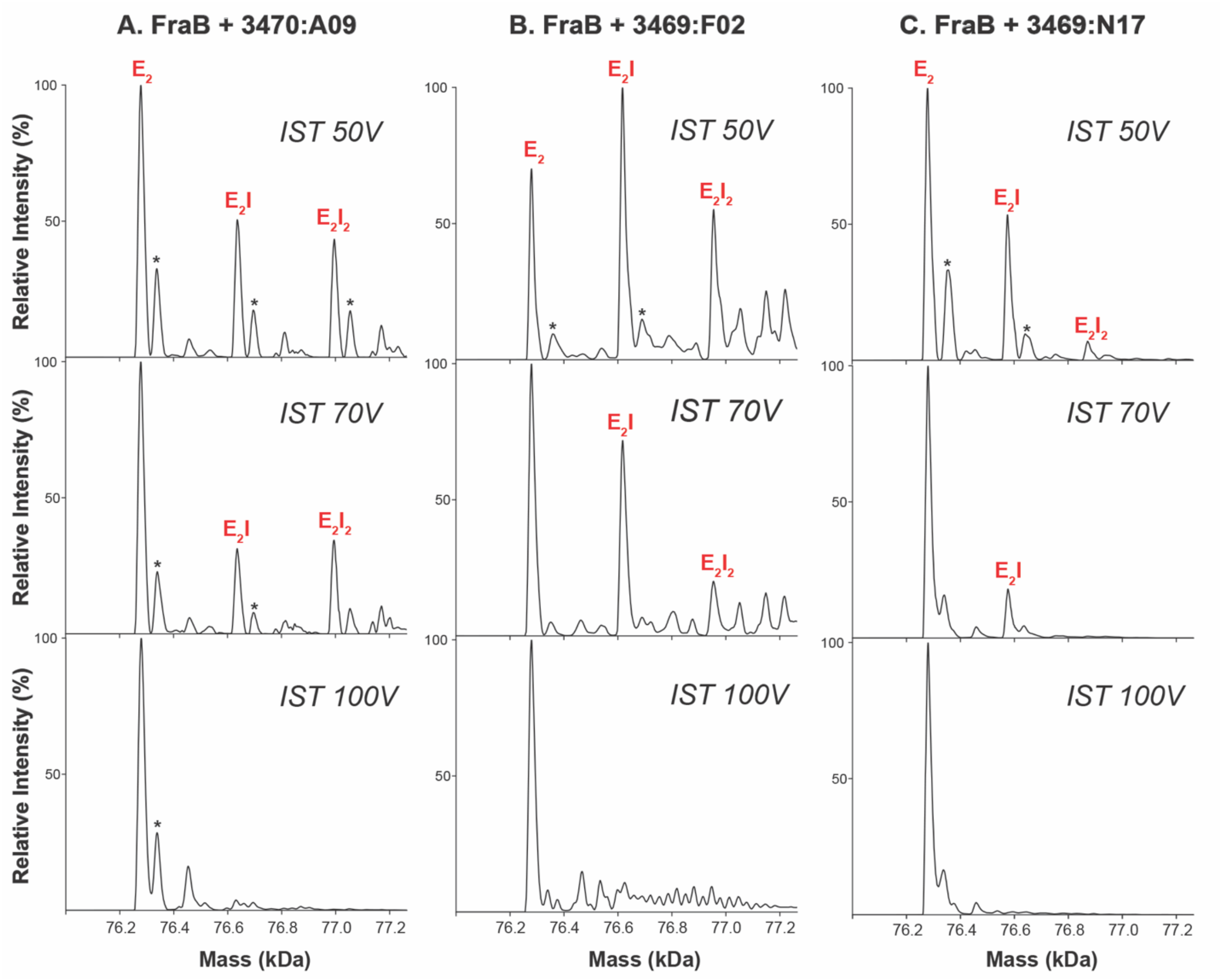

Figure S17 Deconvolved native mass spectra for FraB with the bound to 3470:A09 (*A*), 3469:F02 (*B*), and 3469:N17 (*C*) using in-source trapping (IST) voltages of 50, 70 and 100 V. E_2, FraB dimer; E_2I or E_2I_2, FraB bound to one or two inhibitor copies, respectively. The decrease in inhibitor-bound species with increasing IST voltages suggests non-covalent interactions between FraB and these three inhibitors. Note: These compounds were obtained from ChemDiv.

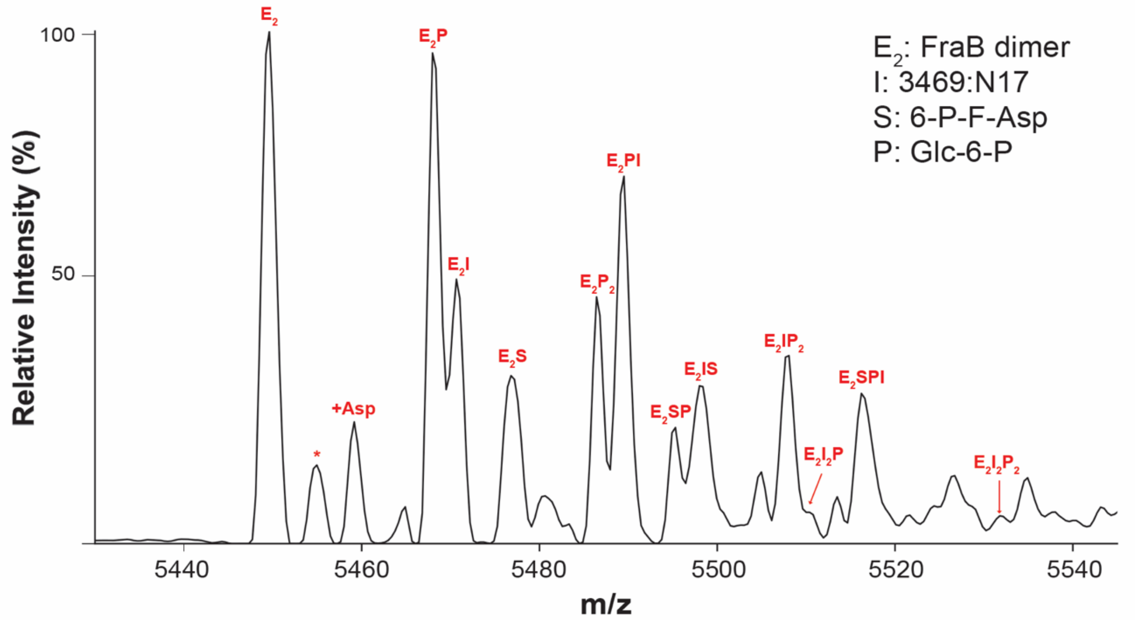

Figure S18 Native mass spectra for FraB bound to 6-P-F-Asp and the 3469:N17 inhibitor. FraB, 6-P-F-Asp, and 3469:N17 were used at final concentrations of 3 μM (monomer concentration), 600 μM, and 100 μM, respectively. Spectra were zoomed into the 14+ charge state. Asterisks (*) denote DMSO adducts (see **Figure S13**). Abbreviations used: S- substrate (6-P-F-Asp); P- product (Glc-6-P), one of the two products generated by FraB. <u>Note</u>: N17 used in this experiment was obtained from Ambinter (see **Figure 7** for similar data obtained using N17 from ChemDiv).
